# Breastfeeding, prenatal depression and children’s IQ and behaviour: a test of a moderation model

**DOI:** 10.1186/s12884-020-03520-8

**Published:** 2021-01-18

**Authors:** Rita Amiel Castro, Vivette Glover, Ulrike Ehlert, Thomas G. O’Connor

**Affiliations:** 1 Department of Clinical Psychology and Psychotherapy, Institute of Psychology, University of Zurich, Zurich, Switzerland; 2 Department of Metabolism, Digestion and Reproduction, Institute of Reproductive and Developmental Biology, Imperial College London, London, UK; 3 Departments of Psychiatry, Psychology, Neuroscience, and Obstetrics and Gynecology, University of Rochester, Rochester, USA

**Keywords:** Breastfeeding, Prenatal depression, Prenatal anxiety, Prenatal exposure effects, Child, Intelligence tests, Behaviour, ALSPAC

## Abstract

**Background:**

We aimed to determine the associations between breastfeeding and children’s neurodevelopment indexed by intelligence quotient (IQ) and emotional and behavioural problems through mid-childhood adjusting for prenatal and postnatal depression and multiple confounders; and to test the novel hypothesis that breastfeeding may moderate the effects of prenatal depression and anxiety on children’s neurodevelopment.

**Methods:**

The study is based on women and their children from the longitudinal Avon Longitudinal Study of Parents and Children (*n*=11,096). Children’s IQ was derived from standardized in-person testing; behaviour problems were assessed according to parent-report; information on breastfeeding, prenatal depression and anxiety and multiple confounders were derived from self-report questionnaires. We conducted hierarchical multiple regression adjusting for several covariates.

**Results:**

43% women were exclusively breastfeeding at 1 month and an additional 16.8% were engaged in mixed or partial breastfeeding. Both exclusive breastfeeding (B = 2.19; SD = 0.36, *p* =.00) and mixed feeding (B = 1.59; SD= 0.52; *p*=.00) were positively associated with IQ at 8 years of age, after adjusting for covariates. Exclusive breastfeeding was negatively associated with hyperactivity/attention deficit at 4 years (B = −.30, SD = .05; *p* < .01); mixed feeding was related to hyperactivity/attention deficit at age 9 (B = .20; SD = .08; *p* = .03) after adjustments. There was no association between breastfeeding and emotional or conduct problems. Breastfeeding did not moderate the association between prenatal depression and anxiety and children’s neurodevelopment.

**Conclusions:**

The selective association between breastfeeding and neurodevelopmental measures suggests a nutritional rather than broader beneficial psychological effect on child neurodevelopment. Breastfeeding did not moderate the associations between prenatal depression and anxiety and child neurodevelopment, suggesting separate mechanisms of action.

**Supplementary Information:**

The online version contains supplementary material available at 10.1186/s12884-020-03520-8.

## Highlights

- In a large cohort controlled for multiple confounders, breastfeeding (exclusive and mixed) was positively associated with IQ in mid-childhood.
- Exclusive breastfeeding was negatively associated with hyperactivity/attention deficit in children at age 4.
- Breastfeeding did not moderate or mediate the effects of prenatal depression or anxiety on children’s IQ and behavioural and emotional problems.

## Background

Considerable research links breastfeeding and positive child neurodevelopmental outcomes, including positive effects on intelligence quotient (IQ) [1–3]. However, the strength of the causal association continues to be challenged because of, e.g., wide variability in research studies and adjustment for confounders [4–6]. Breastfeeding benefits on cognition may derive from nutritional contents. Long-chain fatty acids such as docosahexaenoic acid (DHA) and arachidonic acid (AA) are involved in modulation of cell growth and membrane lipid biosynthesis and myelination [7]; sialic acid, is a vital component for brain ganglioside [8, 9] whereas zinc, choline, and vitamin B12 are important nutrients for myelin synthesis [10]. Breastfeeding also seem to improve maternal sensitivity, which in turn positively predicts infant development [11]. In contrast, whereas infant formulas may be fortified with vitamins, minerals, supplemental protein concentrates, nucleic factors, and omega 3 fatty acids [12], this form of feeding involves less emotional and physical contact. Compared to findings for neurodevelopment, the evidence concerning breastfeeding benefits for emotional development and behaviour is less clear [13–15].

The current study adds to this literature in a large cohort study using repeated measures of neurodevelopment and behavioral outcomes. The study contributes to the literature in a second way by testing the novel hypothesis that breastfeeding may moderate the impact of a well-documented risk for neurodevelopmental problems associated with prenatal maternal distress. Significant maternal stress and psychiatric symptoms, most notably depression and anxiety, during pregnancy and postpartum can increase the risk for long-term neurodevelopmental problems for the child [16–20]. For example, prenatal maternal symptoms are associated with children’s decreased mental and motor scores [21], increased odds of developmental delay [22], lower IQ [23] and behavioural and neurological maladjustment [21, 24, 25]. Given the robust associations between prenatal maternal distress and child neurodevelopment, there is now considerable interest in effect modifiers, and particularly factors that may modulate the impact of prenatal maternal distress on child neurodevelopment. One strong candidate is breastfeeding.

One rationale for considering breastfeeding as an effect modifier is that such an analysis may allow for a more precise description of the mechanisms involved. The moderation hypothesis is that breastfeeding modulates the magnitude of the effect of prenatal maternal distress on children’s outcomes, reducing its effects and conferring neurodevelopmental protection to breastfed children [26]. We test that novel hypothesis, which would provide practical and clinical information and widen the study of the mechanisms involved.

The current paper extends the breastfeeding literature a) by testing the associations between breastfeeding and children’s neurodevelopment and emotional/behavioural child symptoms on multiple occasions, adjusting for pre- and postnatal depression and multiple confounders, and b) by testing the novel hypothesis that breastfeeding may moderate the effects of prenatal depression on these child neurodevelopmental problems. The significance of this question is high, given the growing evidence that prenatal maternal distress and breastfeeding may have opposing effects on child neurodevelopmental outcomes and the potential for clinical application. Our analyses are based on the large Avon Longitudinal Study of Parents and Children (ALSPAC) cohort, which includes extensive data on possible confounders and prospective longitudinal data from pregnancy.

## Methods

### Sample

Our sample is based on the ALSPAC, a longitudinal birth cohort investigating women, their partners and an index child [27–29]. Pregnant women living in the former county of Avon, England who had an expected date of delivery between April 1st, 1991 and December 31st, 1992 were eligible to participate. From the initial 14,541 participants included, *N* = 13,988 had the child alive at 1 year old and *N* = 11,096 had available data on type of infant feeding provided, IQ and emotional and behavioural symptoms. We excluded women with premature babies and/or low-birth weight babies and selected women who have provided information on type of infant feeding at 1 month postpartum. Data for the entire ALSPAC sample was gathered from maternal and paternal questionnaires administered on multiple occasions throughout pregnancy and childhood. From age 8 years, in-person testing was included for the complete sample (from which we included in-person IQ testing; see below). Prior to age 8 years, in-person testing was conducted only on a subset of ALSPAC participants, the “Children in Focus (CIF)” group. This subset was randomly chosen from the last 6 months of ALSPAC births (from June 6th until December 11th, 1992). From the CIF subset, we used data for the children’s intelligence measure Wechsler Preschool and Primary Scale of Intelligence [30] at age four (*N* = 728) [28, 29]. The ALSPAC study website contains details of all available data through a fully searchable data dictionary and variable search tool (http://www.bristol.ac.uk/alspac/researchers/our-data/). Ethical approval for the study was obtained from the ALSPAC Law and Ethics Committee and local research ethics committees before commencement of the study. Written informed consent from parents or legal guardians on behalf of their children was obtained for clinic data. Questionnaire data consent was assumed by the completion of parental and children questionnaires. Participants were informed that they could withdraw from the study at any time (which includes use of their data). The use of data collected via questionnaires and clinics followed the recommendations of the ALSPAC Ethics and Law Committee at the time.

### Measures and procedure

**Fig. 1:**
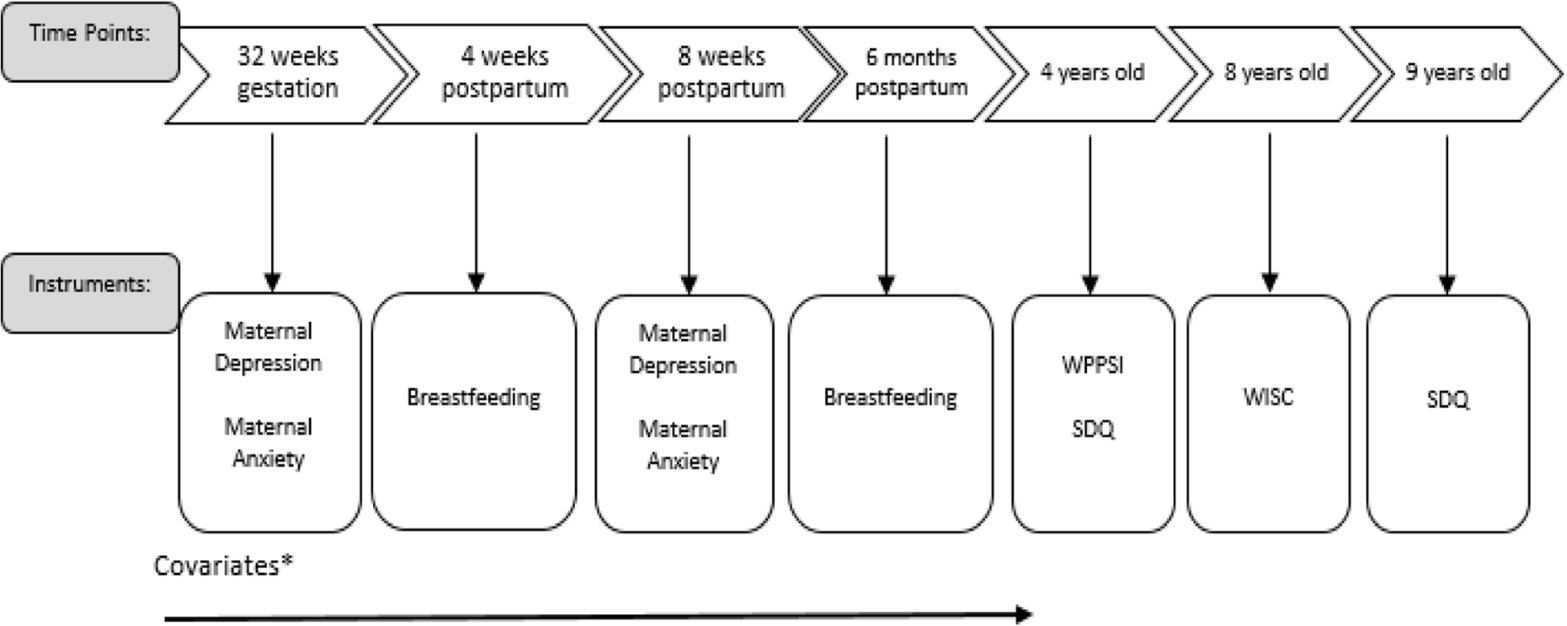
Study timeline – Time points and instruments used. Note: * Covariates were collected from 18 weeks gestation up to 18 months postnatal

The ALSPAC study website provides details of all questionnaires used through a questionnaire search tool (http://www.bristol.ac.uk/alspac/researchers/our-data/questionnaires/). Questionnaires used in this study assessed children’s IQ and emotional and behaviour problems, infant feeding information, maternal prenatal and postnatal depression and anxiety and multiple mother-child confounders. Figure 1 describes in detail the study timeline. Instruments used in this study can be found in the supplementary files.

#### Maternal depressive symptoms

The Edinburgh Postnatal Depression Scale [31] (EPDS) was used to measure maternal depressive symptoms. This is an internationally recommended self-report screening for perinatal depressive symptoms validated for use during and outside of the postnatal period [32]. Each of its 10 items is rated on a 4-point Likert scale (0–3), producing a summative score ranging from 0 to 30. Maternal depressive symptoms were measured at 32 weeks gestation and at 8 weeks postpartum with scores ranging from 0 to 29.

#### Maternal anxiety symptoms

Maternal anxiety symptoms were assessed at 32 gestational weeks and 8 weeks after birth using the anxiety items from the Crown-Crisp Experiential Index (CCEI), a validated self-rating inventory [33]. Example items include “worry a lot” and “feeling strung up inside”.

#### Breastfeeding

Breastfeeding data were collected when the child was approximately 1 and 6 months old. At 1 month, several questions about infant feeding and breastfeeding exclusivity were administered (e.g. “How have you fed your baby since s/he was born?”, “Is your baby fed in a regular schedule – e.g. every 4 hours?”); maternal responses were scored to create 3 feeding conditions: exclusive breastfeeding (ingestion of breast milk only), mixed feeding (ingestion of both breast milk and formula feeding), and exclusive formula feeding (ingestion of formula feeding only). At 6 months, mothers were asked if they were currently providing any breastfeeding to their babies, which generated one variable indicating both mixed and exclusive breastfeeding.

#### Wechsler preschool and primary scale of intelligence (WPPSI)

The WPPSI is an intelligence test designed for children from 2 years 6 months to 7 years 7 months of age [30]. It consists of 14 subtests, divided into three types: core, supplemental or optional. The core subtests are required for computing the verbal, performance and full scale IQ. We use scale scores in the analyses, which are standardized to a mean of 100 and a SD of 15; values below 70 are extremely low and values > 130 may be considered superior [34]. In our study, we report WPPSI results from the full-scale IQ assessed in children of 49 months of age (4 years and 1 month) from the CIF subsample (*N* = 728).

#### Wechsler intelligence scale for children (WISC-III)

The WISC-III is an intelligence tool for children and adolescents from age 6 to age 16 [35]. It consists of 13 individual subtests, 10 standard and 3 supplementary, that combine to develop three composites: Verbal (VIQ), Performance (PIQ), and Full Scale (FSIQ). An abbreviated form of the test was applied and only alternate items were used for all subtests to derive an overall intelligence quotient. We report WISC results from the full-scale IQ assessed in children from the entire sample at 8 years old.

#### The strengths and difficulties questionnaire (SDQ)

The SDQ is a brief behavioural screening questionnaire used with children from 3 to 16 years old and was administered by their parents [36]. The scale refers to 25 positive and negative attributes divided between 5 scales, namely: emotional symptoms, conduct problems, hyperactivity/attention deficit, peer relationship problems and prosocial behaviour. The items from all scales compose the total difficulties score [36]. For our analysis, we used the sub-scales hyperactivity/attention deficit, emotional problems, conduct problems and total difficulties score. The SDQ measure was assessed at 57 months postnatal (4 years and 8 months) and at 9 years and 7 months of age.

#### Covariates

Potential covariates were based on prior review of breastfeeding, infant cognition and infant behaviour literature and from variables related to our research questions and available in our ALPSAC dataset. Covariates included: self-reported maternal smoking, reported in the first gestational trimester, coded as 0= no or 1= yes (including cigarettes or other smoking, e.g., cigars); self-reported partner’s daily cigarette consumption, defined as number of cigarettes smoked per day and collected at 8 months postpartum; self-reported maternal smoking after birth, defined as tobacco smoked after the baby’s birth, collected at 8 weeks postpartum and scored as 1= yes or 0= no; maternal education, documented on 5 categories, reported at 32 gestational weeks and coded as 1 = CSE (Certificate of Secondary Education), 2 = Vocational education, 3 = O-level (Ordinary level, qualification conferred as part of the General Certificate of Education), 4 = A-level (General Certificate of Education - Advanced level) and 5=University degree; partner’s education, documented on 5 categories, reported at 32 gestational weeks and coded as 1 = CSE, 2 = Vocational education, 3 = O-level, 4 = A-level and 5=University degree; self-reported quality and extent of stimulation available to a child in the home environment [Home Observation for Measurement of the Environment (HOME)] [37], assessed at 18 months postnatal and scaled from 1 to 12 points; mother’s age at delivery, asked in years and ranging from 16 to 43 years old; primipara, reported on the second gestational trimester, coded as 1 = yes, 0 = no; gestational age, collected after birth, reported in weeks; crowding, collected at 8 gestational weeks, based on the number of persons in the household divided by the number of rooms, using a 4-point scale 1= ≤ 0,5, 2= > 0,5-0,75, 3= > 0,75–1, 4= > 1; mother’s return to work, coded as 1= yes or 0 = no and reported at 8 months postpartum; baby’s sex – male or female -, baby’s weight, scaled in kilograms. Approximately 97% of the mothers and fathers were white/British or Caucasian (consistent with local demographics at the time), and so we were unable to examine race or ethnicity as a main or modifying effect in our analyses.

#### Statistical analysis

Statistical analyses were performed using the IBM Statistical Package for the Social Sciences (SPSS Version 24 for Windows). Continuous variables were normally/quasi-normally distributed whereas categorical variables were non-normal. We conducted analyses using parametric statistics. Pearson correlations were conducted between depressive and anxiety symptoms during pregnancy and postpartum, breastfeeding variables, covariates, and child outcomes, i.e., IQ at 4 and 8 years, and the SDQ subscales at 4 and 9 years. Analyses of IQ using the WPPSI at age 4 are available on the Children in Focus subsample (*N*=728); IQ at age 8 years and parent-reported symptoms at both assessments were available on the whole sample. Hierarchical linear regression analysis was used to examine interactions between breastfeeding and prenatal depression and anxiety for predicting child outcomes; we also considered interactions terms between postnatal depression and anxiety for predicting child outcomes. While modelling, diagnostics were undertaken to improve model specification, including testing for multicollinearity between the predictors and checking for normality of the unstandardized residuals. In the first regression model, we added all covariates together with prenatal depression or anxiety as main effects; in the second model, we added breastfeeding as a main effect; in the third model we included the interactions terms together with postnatal depression. In addition to testing moderation, we also conducted, for exploratory purposes, analyses to consider the degree to which the association between prenatal depression or anxiety and child outcomes was mediated through its impact on (reduced) breastfeeding. A formal analysis of mediation was based on the Sobel test [38]; we favoured this method, given that is appropriate to large datasets, which are normally or quasi normally distributed like ours.

We dealt with missing data through multiple imputation. Multiple imputation with 5 replicates was used to impute missing data for all SDQ sub-scales (*N*=11,096 at ages 4 and 9) and for all covariates as well as prenatal and postnatal depression and anxiety. Missing IQ values were imputed only for age 9 because data from IQ at 4 years derived from the Children in Focus subsample (*N*=728). Missing data ranged from 15% (crowding index) to 48% (SDQ scores). We used the automatic imputation method (SPSS Version 24 for Windows), which after scanning the data uses the monotone method if the data show a monotone pattern of missing values; otherwise, fully conditional specification is used (Markov chain Monte Carlo). Results were combined across imputations (pooled results) based on Rubin’s combination rules [39].

## Results

A detailed overview of the socio-demographic attributes of the sample grouped by 1-month breastfeeding can be seen in Table 1. Data for each separate outcome had approximately normally distributed residuals. Multicollinearity was measured by variance inflation factors (VIF) and tolerance, and reached levels lower than 10 for all variables included in the regression models.

**Table 1:** Sociodemographic characteristics of the sample according to feeding status (1 month)

Variables	Excl. Breastfeeding			Mixed Feeding			Excl. Formula Feeding			*F (df) / X*^*2*^*(df)*
	*N*	*%*	*Mean (SD)*	*N*	*%*	*Mean (SD)*	*N*	*%*	*Mean (SD)*	
Mother and Partner’s characteristics										
**Mother’s Age**	4772	–	29.46^A^ (4.49)	1861	–	28.95^B^ (4.58)	4463	–	26.73^C^ (4.85)	420.75 (2)**
**Primipara (Y)**	4617	42.7^A^	–	1791	46.5^B^	–	4183	39.7^C^	–	18.55 (2)**
**Self-reported smoking in pregnancy (Y)**	4686	15.8^A^	–	1825	21.0^B^	–	4268	31.1^C^	–	109.92 (2)**
**Self-reported partner’s daily cigarette consumption**	4242	–	3.06^A^ (7.09)	1617	–	4.02^B^ (8.08)	3476	–	5.25^C^ (8.47)	75.13 (2)**
**Mother’s Education**	4487^A^		–	1721^B^		–	3789^C^		–	1203.61 (8)**
CSE		7.9			9.5			22.6		
Vocational		6.9			8.5			14.6		
O-Level		32.6			37.7			42.5		
A-Level		29.9			28.8			16.3		
University Degree		22.7			15.6			3.9		
**Partner’s Education**	4256^A^		–	1623^B^		–	3398^C^		–	830.94 (8)**
CSE		10.9			14.7			25.6		
Vocational		7.2			8.6			12.2		
O-Level		21.1			23.8			26.4		
A-Level		29.4			32.5			28		
University Degree		31.4			20.3			7.9		
**Crowding Index**	4580^A^		–	1795^A^		–	4163^B^		–	345.88 (6)**
≤ 0,5 person per room		49			49.1			33.7		
> 0,5–0,75 person per room		30.9			32.2			32.8		
> 0,75–1 person per room		16			14			25		
> 1 person per room		4.1			4.7			8.6		
**HOME Score**	4363	–	10.41^A^ (1.55)	1643	–	10.37^A^ (1.59)	3661	–	10.13^B^ (1.64)	30.80 (2)**
**Maternal return to work (Y)**	4432	34.3^A^	–	1695	40^B^	–	3789	28.8^C^	–	70.71 (2)**
**Self-reported maternal smoking since birth (Y)**	4594	14.3^A^	–	1778	19.3^B^	–	4057	31.3^C^	–	370.46 (2)**
**Maternal depressive symptoms (32 gestational weeks)**	4503	–	6.47^A^ (4.81)	1740	–	6.68^A^ (4.91)	4008	–	7.45^B^ (5.16)	42.80 (2)**
**Maternal depressive symptoms (8 weeks postpartum)**	4590	–	5.61^A^ (4.40)	1776	–	6.08^B^ (4.80)	4054	–	6.30^C^ (4.98)	23.64 (2)**
Infant characteristics										
**Baby’s sex (M)**	4772	50.2^A^	–	1861	53.1^B^	–	4463	52.6^C^	–	7.08 (2)*
**Baby’s Birthweight**	4772	–	3498.61^A^ (445.40)	1861	–	3488.09^A^ (465.16)	4463	–	3461.16^B^ (469.63)	7.88 (2)**
**Gestational Age**	4772	–	39.67^A^ (1.39)	1861	–	39.67^A^ (1.44)	4463	–	39.66^A^ (1.48)	0.89 (2)
**IQ (Total – 4y)**	440	–	107.05^A^ (14.46)	181	–	106.73^A^ (12.68)	298	–	100.53^B^ (13.58)	21.70 (2) **
**IQ (Total - 8y)**	3052	–	107.47^A^ (16.16)	1134	–	105.61^B^ (16.21)	1989	–	99.98^C^ (15.58)	134.25 (2) **
**Hyperactivity (SDQ – 4y)**	3786	–	3.62^A^ (2.28)	1393	–	3.94^B^ (2.26)	3037	–	4.29^C^ (2.31)	72.07 (2)**
**Emotional Symptoms (SDQ – 4y)**	3821	–	1.40^A^ (1.49)	1412	–	1.45^A^ (1.49)	3060	–	1.46^A^ (1.50)	0.24 (2)
**Conduct Problems (SDQ – 4y)**	3816	–	1.84^A^ (1.36)	1408	–	1.98^B^ (1.48)	3064	–	2.02^C^ (1.41)	14.61 (2)**
**Total Behavior Score (SDQ – 4y)**	3718	–	8.27^A^ (4.40)	1370	–	8.85^B^ (4.50)	2970	–	9.37^C^ (4.61)	48.95 (2)**
**Hyperactivity (SDQ – 9y)**	3273	–	2.71^A^ (2.12)	1229	–	3.01^B^ (2.34)	2336	–	3.05^C^ (2.22)	20.96 (2)**
**Emotional Symptoms (SDQ - 9y)**	3269	–	1.44^A^ (1.63)	1224	–	1.51^A^ (1.72)	2338	–	1.58^A^ (1.79)	1.75 (2)
**Conduct Problems (SDQ – 9y)**	3279	–	1.21^A^ (1.34)	1224	–	1.26^A^ (1.31)	2339	–	1.33^B^ (1.41)	4.33 (2)**
**Total Behavior Score (SDQ – 9y)**	3245	–	6.41^A^ (4.65)	1219	–	6.90^B^ (4.81)	2318	–	7.02^C^ (5.01)	12.99 (2)**

*Note*: *SD* standard deviation, *F* F statistic, *df* degrees of freedom, *X*^*2*^ chi-square, *M* male, *m* months, *Y* yes, *y* years, *IQ* Intelligence Quotient, *SDQ* Strengths and Difficulties Questionnaire, *A-B-C* groups not sharing the same superscript are different from each other at *p* <.05; **Significant at the *p* =.01 level; *Significant at the *p* =.05 level

There were marked differences in most variables studied between the three infant feeding groups [exclusive breastfeeding (*N*= 4772), mixed feeding (*N*= 1861) and exclusive formula (*N*= 4463)]. Between-group differences in child IQ and behavioural and emotional symptoms are also reported in Table 1. Large differences were observed between the exclusive breastfeeding group and the exclusive formula group in relation to full scale IQ (F(2, 726)= 21.70, *p* < .01) at 4 years (in the Children in Focus subsample) and full scale IQ (F(2, 6172)= 134.25, *p* < .01) at 8 years; differences were also observed in early and late childhood, respectively, for SDQ: hyperactivity/attention deficit (F(2,8213)= 72.07, *p* < .01; F(2,6835)= 20.96, *p* < .01); conduct problems (F(2, 8285)= 14.61, *p* < .01; F(2,6839)= 4.33, *p*= .01) and total behaviour difficulties (F(2, 8055)= 48.95, *p* < .01; F(2, 6779)= 12.99, *p* < .01) respectively at 4 and 9 years. Somewhat parallel differences were found between the exclusive breastfeeding and the mixed feeding groups in relation to full scale IQ (F(2, 6172)= 134.25, *p* < .01) at 8 years old, hyperactivity/attention deficit (F(2,8213)= 72.07, *p* < .01; (F(2,6835)= 20.96, *p* < .01), and total difficulties (F(2,8055)= 48.95, *p* < .01; (F(2,6779)= 12.99, *p* < .01) at four and 9 y and conduct problems (F(2,6839)= 14.61, *p* < .01) at 4 y. Notably, there were no differences in emotional problems between the three groups at either time point.

Pearson correlations indicated that prenatal depressive symptoms were significantly but weakly associated with exclusive breastfeeding (r = −.09, *p* ≤.01) and full scale IQ at 4 (r = −.12, *p* ≤.05) and 8 years (r = −.12, *p* ≤.01) as well as positively associated with all SDQ scores at both ages (ranging from r =.14–.22, *p*≤.01). Prenatal anxiety symptoms were also negatively correlated with IQ at 4 and 8 years (r = −.11, *p* ≤.05; r = −.09, *p* ≤.01), but positively associated with all SDQ scores at both ages (ranging from r = .13–.21, *p* ≤.01). Exclusive breastfeeding was significantly associated with IQ at 4 (*r* =.18, *p*≤.01) and 8 years old (r =.18, *p*≤.01) and with all SDQ scores at both ages (ranging from *r* = −.01 − −.12, *p*≤.05). Full scale IQ at age 4 positively correlated with full scale IQ at 8 years (*r* = .63, *p* ≤.01), but showed a negative relation with hyperactivity/attention deficit (r = −.21, *p* ≤.01; r = −.23, p ≤.01) and all other SDQ sub-scales (ranging from r = −.02 − −.21, *p* ≤.01; ranging from r = −.10 − −.25, p ≤.01) at ages 4 and 9 respectively. Similarly, full scale IQ at age 8 was negatively correlated with hyperactivity/attention deficit at 4 (*r* = −.18, *p* ≤.01) and 9 years (r = −.22, *p* ≤.01) and all other SDQ sub-scales (ranging from r = −.06 − −.18, *p* ≤.01; ranging from r = −.10 − −.22, *p* ≤.01) at both ages.

Table 2 reports the regression model predicting IQ from depressive symptoms, breastfeeding (mixed and exclusive) and covariates. In our dataset, the last hierarchical model including all predictors showed that exclusive breastfeeding and mixed feeding at 1 month were positively associated with full IQ at age 8, but not at 4 years. Depressive symptoms during pregnancy and postpartum were not significantly associated with IQ at either age. Compared to those infants who were exclusively formula fed, results showed a 2.1 point difference in IQ at age 8 for children exclusively breastfed and an increase of 1.5 IQ points in children who received mixed feeding. At age 4, results revealed significant associations between higher birthweight, maternal and paternal education and less household crowding with increased IQ scores. Findings at age 8 indicate positive associations between IQ scores and home environment, maternal age, birthweight, being primipara and maternal and paternal education. The interaction terms between exclusive breastfeeding and prenatal depression (B = −.10, SD = .20, *p* = .62; B = .10, SD = .10, *p* = .36) and exclusive breastfeeding and postnatal depression (B = .06, SD = .20, *p* = .74; B = −.07, SD = .08, *p* = .36) at 4 and 8 years, respectively, did not show a moderation effect. Interaction terms estimated for mixed feeding and prenatal depression and mixed feeding and postnatal depression were also not significant (*p* >.05). Similarly, at 6 months, mixed feeding yielded a positive association with IQ only at age 8 (B = 1.54, SD = .65, *p* = .01) with no significant effects resulting from the interaction term (prenatal and postnatal depression). Analysis of non-imputed data revealed comparable results, indicating robust effects.

**Table 2:** Hierarchical regression analysis of predictors of children’s IQ at 4 and 8 years old

Variable	IQ 4 years (***N***=728)			IQ 8 years (***N***=10,748)		
	*B*	*SE*	*Sig.*	*B*	*SE*	*Sig.*
**Maternal age**	.18	.11	n.s.	.20	.05	**
**Primipara**	.56	1.04	n.s.	1.14	.43	*
**Gestational age**	−.47	.31	n.s.	−.20	.12	n.s.
**Self-reported smoking in pregnancy**	−.81	1.26	n.s.	.64	.60	n.s.
**Self-reported partner’s daily cigarette consumption**	−.11	.06	n.s.	.02	.02	n.s.
**Self-reported maternal smoking since birth**	−2.22	1.64	n.s.	.68	.64	n.s.
**Crowding**	−1.53	.56	**	−1.36	.29	**
**HOME score**	1.43	.28	**	.56	.13	**
**Maternal education**	2.51	.45	**	2.85	.29	**
**Paternal education**	1.96	.40	**	2.40	.18	**
**Maternal return to work**	.57	.91	n.s.	−.41	.34	n.s.
**Baby’s sex**	2.91	.84	**	−.04	.39	.n.s.
**Baby’s birthweight**	.00	.00	**	.00	.00	**
**Maternal depressive symptoms (32 weeks gestation)**	−.14	.11	n.s.	−.14	.04	**
**Mixed feeding at 1 month**	1.82	1.30	n.s.	1.60	.53	**
**Exclusive breastfeeding at 1 month**	2.17	1.17	n.s.	2.2	.36	**
**Maternal depressive symptoms (8 weeks postnatal)**	.02	.11	n.s.	.02	.05	n.s.

Note. *p* ≤ .05*; *p* < .01**

Analysis of maternal prenatal anxiety symptoms yielded effects that were parallel to those for depression reported above, for both imputed and non-imputed data. For full scale IQ at 8 years, significant prediction was found for exclusive breastfeeding (B = 2.07, SD = .42, *p* < .01) and mixed feeding (B = 1.50, SD = .51, *p* < .01); the breastfeeding prediction was not significant for full scale IQ at 4 years. On the other hand, mixed feeding at 6 months was marginally associated with IQ at 8 years (B = 1.41, SD = .71, *p* = .09). Prenatal anxiety was not significantly associated with IQ at either age after adjusting for confounders. As with the analyses of prenatal depression, we did not find a significant moderation between breastfeeding at 1 and 6 months and prenatal anxiety at 4 or 8 years (*p* >.05).

The regression model predicting hyperactivity/attention deficit symptoms is reported in Table 3. Exclusive breastfeeding at 1 month was negatively associated with hyperactivity/attention deficit only at 4 years; mixed feeding was associated with hyperactivity/attention deficit only at 9 years old. Likewise, mixed feeding at 6 months presented a significantly negative association at age 9 (B = −.21, SD = .08, *p* = .01). Prenatal maternal depression was positively associated with hyperactivity/attention deficit at both ages. The interaction terms between exclusive breastfeeding and prenatal depression (B = .01, SD = .01, *p* = .33; B = −.01, SD = .01, *p* = .12) as well as mixed feeding at 1 and 6 months and prenatal depression (B= .00, SD = .01, *p* = .90; B = .01, SD = .01, *p* = .40; B = .01, SD = .00, *p* = .06; B = .01, SD = .00, *p* = .06) at 4 and 9 years, respectively, did not show significant moderation effect. Interaction terms estimated for exclusive breastfeeding and postnatal depression and mixed feeding at 1 and 6 months and postnatal depression were also not significant (*p* >.05).

**Table 3:** Hierarchical regression analysis of predictors of children’s hyperactivity at 4 and 9 years old

Variable	Hyperactivity 4y (***N***=10,326)			Hyperactivity 9y (***N***=10,350)		
	*B*	*SE*	*Sig.*	*B*	*SE*	*Sig.*
**Maternal age**	−.02	.01	**	−.01	.01	n.s.
**Primipara**	−.05	.04	n.s.	−.09	.04	*
**Gestational age**	.02	.02	n.s.	.02	.02	n.s.
**Self-reported smoking in pregnancy**	.10	.08	n.s.	−.10	.09	n.s.
**Self-reported partner’s daily cigarette consumption**	.01	.00	n.s.	.01	.00	n.s.
**Self-reported maternal smoking since birth**	.04	.09	n.s.	−.18	.12	n.s.
**Crowding**	.01	.02	n.s.	.03	.03	n.s.
**HOME score**	−.13	.02	**	−.13	.02	**
**Maternal education**	−.09	.03	**	−.07	.03	n.s.
**Paternal education**	−.11	.03	**	−.05	.03	n.s.
**Maternal return to work**	−.05	.05	n.s.	.04	.06	n.s.
**Baby’s sex**	−.56	.05	**	−.73	.06	**
**Baby’s birthweight**	.00	.00	**	.00	.00	**
**Maternal depressive symptoms (32 gestational weeks)**	.02	.01	**	.03	.01	**
**Mixed feeding at 1 month**	−.10	.07	n.s.	.20	.09	*
**Exclusive breastfeeding at 1 month**	−.31	.05	**	−.05	.06	n.s.
**Maternal depressive symptoms (8 weeks postpartum)**	.04	.01	**	.04	.01	**

*Note. p* ≤ .05*; *p* < .01**

The pattern of results for prenatal anxiety was very similar to that for prenatal depression. Prenatal (B = .04, SD = .00, *p* < .01; B = .03, SD = .01, *p* < .01) and postnatal anxiety (B = .04, SD = .00, *p* < .01; B = .05, SD = .01, *p* < .01) were positively associated with hyperactivity/attention disorder at 4 years and late childhood. However, there was no evidence at either time point that the association between prenatal anxiety and child hyperactivity/inattention was moderated by breastfeeding at 1 or 6 months (*p* >.05). Results from the non-imputed dataset were similar to the imputed data.

No statistically significant relationship was found between breastfeeding (mixed at 1 and 6 months and exclusive) and emotional problems, total difficulties and conduct behaviour at either age (*p* >.05). Although prenatal depression and anxiety were both significantly associated with emotional problems and conduct behaviour at 4 and 9 years, there was no evidence that this prediction was moderated by breastfeeding.

### Supplementary analyses

Additional analyses indicated that the results reported above did not differ by child sex; in particular, the lack of moderation effect of prenatal distress on child outcomes was found in both boys and girls. A second set of analyses considered potential moderation effects of breastfeeding on the associations between postnatal depression and anxiety and child outcomes. Results were consistent with the analyses of prenatal maternal symptoms: postnatal depression and anxiety were associated with IQ and behavioural problems, but there was no reliable evidence that these associations were moderated by breastfeeding (details available form the first author). A third set of analyses considered prenatal and postnatal depression as binary variables, using a clinical cut-off for depression (EPDS > 12). The results did not differ from the analysis conducted with depression as a continuous variable, including the absence of moderation of prenatal and postnatal depression effects on child outcomes. A final set of supplementary analyses considered a mediation model, that is, the association between prenatal depression or anxiety on child neurodevelopmental outcomes was explained by the impact of prenatal distress on breastfeeding. As the bivariate analyses indicated, the associations between prenatal maternal distress and breastfeeding were significant (given the large sample size) but small in magnitude (e.g., r = −.09 between prenatal depressive symptom and exclusive breastfeeding); in every case, Sobel test results failed to identify any significant evidence of mediation, for prenatal depression or anxiety, for any of the child neurodevelopmental outcomes.

## Discussion

Our results show a clear association between breastfeeding, children’s IQ and hyperactivity/attention deficit symptoms, but not with emotional problems or symptoms of conduct disorder after allowing for confounders. The selective association with neurodevelopmental measures suggests a specific [40] rather than broader (e.g., psychological) effect of breastfeeding on child outcomes. A novel aim of the study was to examine the hypothesis that the prediction of prenatal maternal distress – depression and anxiety – on child neurodevelopment was moderated by breastfeeding. No reliable evidence of moderation was found; instead, main effects were the rule, even in this large sample. Breastfeeding was positively associated with full IQ at 8 years and negatively associated with hyperactivity/attention deficit at 4 (exclusively breastfeeding) and 9 years (mixed feeding), with effects largely separate from the effect of prenatal maternal distress.

Breastfeeding confers many health and emotional benefits to mothers and babies [41, 42] and is reliably associated with higher cognitive ability in children [43–45]. Interestingly, contrary effects have been linked to maternal prenatal anxiety, stress and depression, which are negatively associated with infant cognitive development [46, 47], and other domains, including emotional development [48]. A meta-analysis revealed a small negative association between prenatal maternal illness and infant cognitive development [49], which is consistent with our findings. We examined both of these early influences on infant development. The extent to which they may be confounded is unclear, with some reports suggesting a bidirectional relationship between breastfeeding and depression may exist [50], but other reports finding weak or non-significant associations between perinatal distress and breastfeeding [51]. Whatever their degree of overlap in terms of exposure, our analyses of their impact on child neurodevelopment suggests is essentially independent and separate effects. Potential venues for this difference may be hormone related, as in-utero exposure to the stress hormone cortisol in maternal prenatal anxiety can contribute to adverse effects on fetal brain development and interfere with synaptogenesis and neurotransmitter function [52]. In contrast, increased physical and skin-to-skin contact between mothers and babies promoted by breastfeeding seem to contribute to infant neurodevelopment [53]. Future studies are warranted to elucidate specific mechanisms of each important exposure variable for child neurodevelopment.

Our finding that breastfeeding at both 1 and 6 months was associated with IQ at 8 years old after controlling for several confounders is consistent with other studies. Since the first publication from Hoefer and Hardy [54], various large-scale studies have reported that breastfed infants present higher scores in cognitive and intelligence tests from childhood to adolescence and more pronounced results are associated with increased duration of breastfeeding [3, 43, 55, 56] . Children who received mixed feeding at 1 and 6 months displayed similar increase in IQ. Of note, a graded association between breastfeeding duration and improved cognitive scores in childhood has been previously described in literature, and may be more likely with longer exclusive breastfeeding [57]. Our analyses also corroborate earlier studies reporting a difference in IQ points in exclusively breastfed children [58]. A meta-analytic review including 18 studies controlling for home environment indicated a similar magnitude of effect [1]. That is, breastfeeding is positively associated with performance in intelligence tests in childhood in such as subjects who had been breastfed had an average gain of 3.44 IQ points [1]. This IQ gain seem to have a long-term impact in which breastfed children have improved performance in school tests [59] and higher education in adolescence and adulthood [60]. In a cross-country study comparison, an increase of 1 IQ point in the cognitive ability of the 95th percentile of the population raised the average gross domestic product by $468 U.S. [61]. On an individual level, siblings comparison revealed that an increase in 1 IQ point yielded an extra $810 U.S. per year by age 35 [62]. This demonstrates that although apparently small, the effect may have meaningful and substantial impact on subjects’ life functioning.

A secondary finding was that exclusive breastfeeding was negatively associated with hyperactivity/attention deficit at age 4. These results partially agree with another large cohort study, which found that at age 5 children (*n*=9525) who were born full term and breastfed up to 3.9 months had lower risk of hyperactivity (OR= 0.65, 95% CI, 0.43–1.00) upon comparison with never breastfed children [13]. A meta-analytic study assessing breastfeeding and infant ADHD (diagnosis based on the DSM criteria) concluded that children with ADHD have a significantly lower duration – less than 3 months - of exclusive breastfeeding compared to non-ADHD controls [63]. On the other hand, not all studies report comparable results [3, 14, 64]. Notably, in this study breastfeeding was not significantly associated with emotional problems in the child. The absence of an emotional behavioural benefit does not support the hypothesis that higher maternal sensitivity and a closer early mother-infant bond, as consequences of extensive interactions through lactation, would contribute to lower odds of emotional problems. An advantage in our analyses is that we adjusted for several covariates. In line with other studies [14, 65], our findings suggest no beneficial impact of early breastfeeding on emotional development in mid-childhood, as assessed by the SDQ. Reducing subsequent emotional problems does not appear to be one of the reasons for advocating for breastfeeding initiation, continuation, and exclusivity.

There are several limitations of the study. We cannot rule out shared method variance as a confounder for the association between breastfeeding and attention problems, although such an effect might have also led to associations with all behavioural scales, which we did not find. Second, we did not have data on nutritional content of breastmilk, and so are unable to provide direct evidence of nutritional benefits of breastfeeding. It should also be noted that this study was based on data collected in the 1990s, and the nutritional content of formula milk may have changed since then. Residual confounding might also be considered due to lack of data on important factors such as maternal IQ, quality of schooling, and child’s medical history. Third, the lack of moderation of the prenatal prediction by breastfeeding may not extend to other child health outcomes, such as immune health [66]. Fourth, data here reported were collected in the 1990s and we acknowledge increases in breastfeeding and prenatal depression prevalence since then. This should not affect the associations found with infant neurodevelopment, but we are limited in our ability to estimate what these differences might be. Fifth, we did not have information in mothers who changed their status across the study. Finally, the ALSPAC sample is not racially/ethnically diverse, and the findings obtained here may not generalize to certain minority groups. These limitations are offset, to a considerable degree, by several strengths of the paper, including a large community sample, multiple occasions of measurement, and in-person testing for IQ.

## Conclusions

In sum, our results add new information to the research on breastfeeding and child neurodevelopment: in a large cohort controlled for several mother and child confounders, breastfeeding (mixed and exclusive) is associated with increased IQ in mid-childhood and negatively associated with hyperactivity/attention deficit; furthermore, breastfeeding neither mediated nor moderated the prediction of child behavioural and emotional symptoms from prenatal anxiety or depression, at either age or for any dimension of symptoms assessed. Our findings imply that breastfeeding and prenatal depression and anxiety effects operate largely independently from one another. Further research examining these relationships in a more ethnically diverse population is warranted. It also remains to be determined which are the critical components in breast milk, which are associated with child’s cognitive development, and hyperactivity/attention deficit. Such an understanding will be of clinical importance in the manufacture of infant formula, for babies who it is not possible to breastfeed.

## Supplementary Information

**Additional file 1:.** ques-m05-me-and-my-baby. Questionnaire data collected at 8 weeks postpartum including maternal anxiety and depression and socio-demographic variables used in this study (e.g. smoking and crowding).

**Additional file 2:.** ques-m04-your-pregnancy. Questionnaire data collected at 32 gestational weeks including maternal anxiety and depression and socio-demographic variables such as maternal and paternal education used in this study.

**Additional file 3:.** ques-m03-having a baby. Questionnaire data collected at 18 gestational weeks including sociodemographic variables used in this study such as smoking, quality of home environment (HOME) and maternal age.

**Additional file 4:.** ques-cb19-your-son-at-9. Questionnaire data collected when the child was 9 years old including the SDQ sub-scales used in this study.

**Additional file 5:.** ques-cb10-development-and-health-of-my-son. Questionnaire data collected when the child was 57 months old including the SDQ sub-scales used in this study.

**Additional file 6:.** ques-cb01-my-young-baby-girl. Questionnaire data collected at 4 weeks postpartum including infant feeding information used in this study.

## Data Availability

The data that support the findings of this study are available from the ALSPAC Executive but restrictions apply to the availability of these data, which were used under license for the current study, and so are not publicly available. Data are however available from the authors upon reasonable request and with permission of the ALSPAC Executive.

## Abbreviations

IQ: Intelligence Quotient
ALSPAC: Avon Longitudinal Study of Parents and Children
CIF: Children in Focus
WPPSI: Wechsler Preschool and Primary Scale of Intelligence
EPDS: Edinburgh Postnatal Depression Scale
CCEI: Crown-Crisp Experiential Index
WISC III: Wechsler Intelligence Scale for Children
VIQ: Verbal Intelligence Quotient
PIQ: Performance Intelligence Quotient
FSIQ: Full Scale Intelligence Quotient
SDQ: The Strengths and Difficulties Questionnaire
CSE: Certificate of Secondary Education
O-level: Ordinary level
A-level: Advanced level
HOME: Home Observation for Measurement of the Environment
SPSS: Statistical Package for the Social Sciences
ADHD: Attention Deficit Hyperactivity Disorder
