# Supplementary material for: Breastfeeding, prenatal depression and children’s IQ and behaviour: a test of a moderation model
Source: BMC Pregnancy Childbirth. 2021 Jan 18;21:62. doi: 10.1186/s12884-020-03520-8 (PMC7814604; doi:10.1186/s12884-020-03520-8)
Supplement: Supplementary file 1 — Additional file 1:. ques-m05-me-and-my-baby. Questionnaire data collected at 8 weeks postpartum including maternal anxiety and depression and socio-demographic variables used in this study (e.g. smoking and crowding). [file 12884_2020_3520_MOESM1_ESM.pdf]

© University of Bristol

M E A N D M Y B A B Y

This questionnaire asks you how you are feeling, whether you are getting enough sleep and how you reacted to the actual birth of your baby.

All the answers you give are confidential. Your name and address will not be on the questionnaire.

We would be grateful if you would help us by answering as many of these questions as possible but if there is any question you do not want to answer that is fine. Just leave it blank.

THANK YOU VERY MUCH FOR YOUR HELP

06/02/92

SECTION A:LABOUR AND DELIVERY

A1. Where did you have your baby?

At home 1 Southmead 2

Weston General 3 BMH 4

Other 5
(please describe)

....

A2. How did you feel when you first went into labour (or to have your caesarean section)?

		Not at all	A little	Moderately	Very much so
a)	afraid	1	2	3	4
b)	uncertain	1	2	3	4
c)	calm	1	2	3	4
d)	excited	1	2	3	4
e)	happy	1	2	3	4

A3. How did you feel while you were having the baby:

neglected 1

Okay 2

warmly supported 3

other (please describe) 4

....

×´ Please make sure you answer the opposite page

A4. In general, did you feel in control of what the doctors and midwives were doing to you during labour?

yes, always 1

yes, most of the time 2

only some of the time 3 didn't have any labour 7

no, hardly at all 4

did not have doctor or midwife 5

A5. During labour, when you needed assistance did you:

feel unable to ask 1

feel you could ask, but didn't 2

ask for help 3

didn't have any labour 7

A6. Who delivered your baby?

not sure 1

Doctor 2

midwife 3

medical student 4

student midwife 5

other (please describe) 6

....

A7. How did the equipment used on you during labour make you feel:

very confident 1

did not effect me 2

upset me 3

no equipment was used 4

I was unaware of equipment used 5

something else (please describe) 6 ....

didn't have any labour 7

A8. a) Did you have any form of pain relief in labour?

Yes 1 No 2 Did not have any labour 7

b) Who decided whether or not you had any pain relief?

	**Yes**	**No**	**Don't know**
i) doctors	1	2	9
ii) midwives	1	2	9
iii) me	1	2	9
iv) my partner	1	2	9
v) other (please describe)	1	2	9

....

c) Were you happy with this decision?

Yes 1 No 2 Unsure 9

d) Were any of the following types of pain relief used?

	Yes	No	Don't know
i) general anaesthetic	1	2	9
ii) epidural anaesthetic	1	2	9
iii) pethidine injection	1	2	9
iv) gas and air	1	2	9
v) other (please describe)	1	2	9

....

A8. e) Did you have a caesarean section?

Yes after being in labour 1	No 2	Yes and never had any labour 3	If yes to this go to A16 on page 7

A9. How was the pain?

	in labour	during delivery
worse than I expected	1	1
what I had expected	2	2
better than I expected	3	3
did not feel any pain	4	4
I did not know what to expect	5	5
other (please describe)	6	6

....

A10. Were you able to get into the positions that were most comfortab le for you during labour and delivery?

	in labour	during delivery
no, hardly at all	1	1
yes, some of the time	2	2
yes, all of the time	3	3

A11. In the first stage of labour what was your position?

	All the time	Most of time	Sometimes	Never
a) lying	1	2	3	4
b) sitting	1	2	3	4
c) standing/walking	1	2	3	4
d) other (please describe)	1	2	3	

....

A12. What position were you in at delivery?

lying on back	1		
lying on side	2		
standing	3		
kneeling	4	not known	9

Crouching	5
other position (please describe)	6

....

A13. Who did you have with you?

		in labour		during delivery	
		**Yes**	**No**	**Yes**	**No**
a)	my husband/partner	1	2	1	2
b)	my mother	1	2	1	2
c)	other friend or relative	1	2	1	2

A14. a) Were there lots of different staff coming in and out of the room while you were in labour?

yes a lot	1	
yes, quite a few	2	
no, hardly any	3	**If** **<u>no</u>**, go to A15
other, please describe	4	

....

If <u>yes</u>,

b) how did you feel about this?

distressed/annoyed	1
not bothered by it	2
Pleased	3
other (please describe)	4

....

A15. Did you feel that you lost control of the way you behaved during labour and delivery?

	**in labour**	**during delivery**
yes, most of the time	1	1
yes, for some of the time	2	2
no, not at all	3	3
not applicable (unconscious)	7	7

A16. Was the birth a wonderful experience for you?

Yes 1 No 2 Not sure 3

A17. Space for any comments you might like to make about the delivery of your baby:

....

....

....

....

DENTAL CARE

A18. Did you go to the dentist during this pregnancy?

Yes 1 No 2

If yes

i) how many fillings did you have?
(If none put 00)

ii) how many months pregnant were you when you had the first one? months

SECTION B:YOUR HEALTH AND LIFESTYLE IN PREGNANCY

B1. During the last months of pregnancy (from 7 months onwards) did you experience any of the following:-

		Yes, in last months of pregnancy	No, not in last months of pregnancy	Don't know
a)	nausea/feeling sick	1	2	9
b)	vomiting	1	2	9
c)	diarrhoea	1	2	9
d)	vaginal bleeding	1	2	9
e)	jaundice	1	2	9
f)	urinary infection	1	2	9
g)	influenza	1	2	9
h)	rubella (german measles)	1	2	9
j)	thrush (candida)	1	2	9
k)	genital herpes	1	2	9
l)	other infection (please describe)	1	2	9
m)	injury or shock to you (please describe)	1	2	9
n)	sugar in urine	1	2	9
o)	x-ray	1	2	9
p)	ultrasound scan	1	2	9
q)	something else (please describe)	1	2	9

B2. During pregnancy, before you went into labour, were you admitted to hospital?

Yes 1 No 2 **If no**, go to B3

If yes, give for each admission:

	REASON	DATE ADMITTED	NO.DAYS STAYED
i)	....	..../..../....	....
ii)	....	..../..../....	....
iii)	....	..../..../....	....
iv)	....	..../..../....	....
v)	....	..../..../....	....

B3. How would you describe your health during the last 4 weeks of pregnancy:

always fit and well 1

mostly felt well and healthy 2

often felt unwell 3

hardly ever felt really well 4

B3. a) On a normal week nowadays how many cans do you have:

i) of decaffeinated cola cans

ii) of ordinary colacans

B3. b) On a normal day, how many cups do you drink:

i) of decaffeinated tea cups

ii) of ordinary tea cups

iii) of decaffeinated instant coffee cups

iv) of ordinary instant coffee cups

v) of decaffeinated real coffee (not instant) cups

vi) of ordinary real coffee cups

B4. Did you smoke regularly in the last 2 months of preg nancy and since having the baby?

	**(a) Last 2 months of pregnancy**		**(b) Since having the baby**	
	**Yes**	**No**	**Yes**	**No**
i) cigarettes	1	2	1	2
ii) pipe	1	2	1	2
iii) cigar	1	2	1	2
iv) other	1	2	1	2

c) How many cigarettes (pipes or cigars) per day did you smoke -

i) **in the last 2 months of pregnancy?**
per day:

30+ 30 25-29 25 20-24 20 15-19 15

10-14 10 5-9 05 1-4 01 not at 00

ii) **in the past week?**
per day:

30+ 30 25-29 25 20-24 20 15-19 15

10-14 10 5-9 05 1-4 01 not at 00

B4. d) If you smoke cigarettes what brand and type of cigarette do you usuallysmoke?

i) brand (give full name): ....

B4.d)
ii) type: filtered 1 Unfiltered 2 roll-your-own 3

iii) please give tar content and colour of your packet

....

Please send us an empty packet/carton with your questionnaire.

B5. a) How many cigarettes (pipes or cigars) per day did your partner smoke,

i) **in the last 2 months of your pregnancy?**

per day

30+ 30	25-29 25	20-24 20	15-19 15	
10-14 10	5-9 05	1-4 01	not at all 00	don't know 99

ii) **in the past week?**

per day

30+ 30	25-29 25	20-24 20	15-19 15
10-14 10	5-9 05	1-4 01	not at all 00

B6. Did you smoke at all when you were in labour?

Yes 1 No 2 Did not go into labour 7

B7. Please indicate how often you smoked marijuana/grass/cannabis/ ganja -

		**Every day**	**2-4 times a week**	**Once a week**	**Less than once a week**	**Not at all**
a)	In the last 2 months of pregnancy	1	2	3	4	5
b)	Since you had the baby	1	2	3	4	5

B8. How often did you use the following in the last 2 months of pregnancy?

		**Nearly every day**	**At least once a week**	**At least once a month**	**Not at all**
a)	amphetamines	1	2	3	4
b)	barbiturates	1	2	3	4
c)	crack	1	2	3	4
d)	cocaine	1	2	3	4
e)	heroin	1	2	3	4
f)	methadone	1	2	3	4
g)	ecstasy	1	2	3	4
h)	other (please describe)	1	2	3	4

....

B9. How often have you used the following since having the baby?

		**Nearly every day**	**At least once a week**	**At least once a all month**	**Not at**
a)	amphetamines	1	2	3	4
b)	barbiturates	1	2	3	4
c)	crack	1	2	3	4
d)	cocaine	1	2	3	4
e)	heroin	1	2	3	4
f)	methadone	1	2	3	4
g)	ecstasy	1	2	3	4

h)	other (please describe)	1	2	3	4

....

B10. How often have you drunk alcoholic drinks? Please indicate for each of the following times:

	**Not at all**	**Less than once a week**	**At least once a week**	**1-2 glasses every day**	**At least 3-9 glasses every day**	**At least 10 glasses every day**
a) Last 2 months of pregnancy	1	2	3	4	5	6
b) Since you had the baby	1	2	3	4	5	6

[By glass we mean a pub measure of spirits, half a pint of lager or cider, a wine glass of wine, etc]

c) How many days in the past month have you drunk the equivalent of 2 pints of beer, 4 glasses of wine or 4 pub measures of spirit?

everyday	5	more than 10 days	4
5-10 days	3	3-4 days	2
1-2 days	1	none.	0

B11. a) Did you attend antenatal or parentcraft classes during your pregnancy?

Yes 1 No 2 **If no, go to Section C**

If yes,

b) were they run by the:

	**Yes**	**No**
i) hospital	1	2
ii) health centre or local antenatal clinic	1	2
iii) NCT (National Childbirth Trust)	1	2
iv) other (please describe)	1	2

....

c) how many times did you go? times

d) did your partner ever go with you?

Yes 1 No 2

SECTION C:YOUR HEALTH NOW

C1. Since having the baby have the following occurred?

**Since having the baby:**		**Almost all the time**	**Sometimes**	**Not at all**
a)	painful stitches	1	2	3
b)	backache	1	2	3
c)	headaches or migraines	1	2	3
d)	urinary infection	1	2	3
e)	nausea	1	2	3
f)	vomiting	1	2	3
g)	diarrhoea	1	2	3
h)	haemorrhoids or piles	1	2	3
i)	infected nipple(s)	1	2	3
j)	other breast problem	1	2	3
k)	feeling weepy/tearful	1	2	3
l)	feeling irritable	1	2	3
m)	feeling exhausted	1	2	3
n)	varicose veins	1	2	3
o)	passing urine very often	1	2	3
p)	problem holding urine when you jump, sneeze etc	1	2	3
q)	indigestion	1	2	3
r)	feeling dizzy/fainting	1	2	3
s)	flashing lights/spots before eyes	1	2	3
t)	shoulder ache	1	2	3
u)	tingling in hands/fingers	1	2	3
v)	tingling in feet/toes	1	2	3
w)	neck ache	1	2	3
x)	feeling depressed	1	2	3
y)	other problem (please describe)	1	2	3

....

....

C2. a) Since having the baby, have you had to stay in hospital again for any reason?

Yes 1 No 2 **If <u>no</u>**, go to C3

If <u>yes</u>,

b) What was the reason for admission ....

....

c) How old was the baby? days

d) Was the baby admitted with you?

Yes 1 No 2

e) If you have had to stay in hospital apart from the birth, how long did you stay?

days

f) What treatment were you given?

....

....

C3. How would you describe your health now?

always fit and well	1
mostly fit and well	2
often unwell	3
hardly ever well	4

C4. Since having the baby how often have you taken any of the following pills, medicines or ointments?

		**Almost every day**	**Sometimes**	**Not at all**
a)	contraceptive pill	1	2	3
b)	iron	1	2	3
c)	vitamins	1	2	3
d)	pills for depression	1	2	3
e)	pain killers	1	2	3
f)	others	1	2	3

C5. Please name all the pills, medicines or ointments you are currently using or have used since the baby was born.

	**What did you take:**	**About how many days did you take or use it?**
1.	....	....
2.	....	....
3.	....	....
4.	....	....
5.	....	....
6.	....	....
7.	....	....
8.	....	....
9.	....	....
10.	....	....

Check: Have you included herbal remedies, sleeping pills, vitamins, cough medicines, pain killers, iro n tablets, homeopathic medicines, the contraceptive pill.

C6. Have you had a postnatal check-up yet?

Yes 1 No 2

C7. How much do you weigh at the moment (write NK if you do not know)
(Please state whether st. lbs. or Kg.)

....

SECTION D: YOUR FEELINGS

The questions in this section ask you about your feelings. You may have already answered questions like this during your pregnancy. Please do so again. This is so that we can see how having a baby may have changed the way you feel.

		Very often	Often often	Not very	Never
D1.	Do you feel upset for no obvious reason?	1	2	3	4
D2.	Do you get troubled by dizziness or shortness of breath?	1	2	3	4
D3.	Have you felt as though you might faint?	1	2	3	4
D4.	Do you feel sick or have indigestion?	1	2	3	4
D5.	Do you feel that life is too much effort?	1	2	3	4
D6.	Do you feel uneasy and restless?	1	2	3	4
D7.	Do you feel tingling or prickling sensations in your body, arms or legs?	1	2	3	4
D8.	Do you regret much of your past behaviour?	1	2	3	4
D9.	Do you sometimes feel panicky?	1	2	3	4
D10.	Do you find that you have little or no appetite?	1	2	3	4
D11.	Do you wake unusually early in the morning	1	2	3	4
D12.	Do you worry a lot?	1	2	3	4
D13.	Do you feel tired or exhausted?	1	2	3	4
D14.	Do you experience long periods of sadness?	1	2	3	4
D15.	Do you feel strung-up inside?	1	2	3	4
D16.	Can you get off to sleep alright?	1	2	3	4
D17.	Do you ever have the feeling you are going to pieces?	1	2	3	4
D18.	Do you often have excessive sweating or fluttering of the heart?	1	2	3	4
D19.	Do you find yourself needing to cry?	1	2	3	4
D20.	Do you have bad dreams which upset you when you wake up?	1	2	3	4

	Very often	Often	Not very often	Never
D21. Do you lose the ability to feel sympathy for others?	1	2	3	4
D22. Can you think quickly?	1	2	3	4
D23. Do you have to make a special effort to face up to a crisis or difficulty?	1	2	3	4

Your feelings in the past week.

D24. I have been able to laugh and see the funny side of things:

As much as I always could	1
Not quite so much now	2
Definitely not so much now	3
Not at all	4

D25. I have looked forward with enjoyment to things:

As much as I ever did	1
Rather less than I used to	2
Definitely less than I used to	3
Hardly at all	4

Your feelings in the past week.

D26. I have blamed myself unnecessarily when things went wrong:

Yes, most of the time	1
Yes, some of the time	2
Not very often	3
No never	4

D27. I have been anxious or worried for no good reason:

No, not at all	1
Hardly ever	2
Yes, sometimes	3
Yes, often	4

D28. I have felt scared or panicky for no very good reason:

Yes, quite a lot	1
Yes, sometimes	2
No, not much	3
No, not at all	4

D29. Things have been getting on top of me:

Yes, most of the time	1
Yes, sometimes	2
No, hardly ever	3
No, not at all	4

D30. I have been so unhappy that I have had difficulty sleeping:

Yes, most of the time	1
Yes, sometimes	2
Not very often	3
No, not at all	4

Your feelings in the past week.

D31. I have felt sad or miserable:

Yes, most of the time	1
Yes, quite often	2
Not very often	3
No, not at all	4

D32. I have been so unhappy that I have been crying:

Yes, most of the time	1
Yes, quite often	2
Only occasionally	3
No, never	4

D33. The thought of harming myself has occurred to me:

Yes, quite often	1
Sometimes	2
Hardly ever	3
Never	4

D34. Have you been feeling at all depressed?

No, not at all	1
Only mildly depressed	2
Yes, quite depressed	3
Yes, very depressed	4

D35. On the whole are there more good days than bad?

Yes, more good days	1
About half and half	2
No, more bad days	3

SECTION E:LIFE EVENTS

Listed below are a number of events which may have brought changes in your life. Have any of these occurred **since the middle of your pregnancy**? If so, please assess how much effect it had on you.

**Since the middle of pregnancy:**	**Yes & affected me a lot**	**Yes, moderately affected**	**Yes, mildly affected**	**Yes, but did not affect me at all**	**No did not happen**
E1.Your partner died	1	2	3	4	5
E2.One of your children died	1	2	3	4	5
E3.A friend or relative died	1	2	3	4	5
E4.One of your children was ill	1	2	3	4	5
E5.Your partner was ill	1	2	3	4	5
E6.A friend or relative was ill	1	2	3	4	5
E7.You were admitted to hospital - including to have your baby	1	2	3	4	5
E8.You were in trouble with the law	1	2	3	4	5
E9.You were divorced	1	2	3	4	5
E10.You found that your partner didn't want your child	1	2	3	4	5
E11.You were very ill	1	2	3	4	5
E12.Your partner lost his job	1	2	3	4	5
E13.Your partner had problems at work	1	2	3	4	5
E14.You had problems at work	1	2	3	4	5
E15.You lost your job	1	2	3	4	5
E16.Your partner went away	1	2	3	4	5
E17.Your partner was in trouble with the law	1	2	3	4	5
E18.You and your partner separated	1	2	3	4	5
E19.Your income was reduced	1	2	3	4	5
E20.You argued with your partner	1	2	3	4	5
E21.You had arguments with your family or friends	1	2	3	4	5
E22.You moved house	1	2	3	4	5

Since the middle of pregnancy:	Yes & affected me a lot	Yes, moderately affected	Yes, mildly affected	Yes, but did not affect me at all	No did not happen
E23. Your partner hurt you physically	1	2	3	4	5
E24. You became homeless	1	2	3	4	5
E25. You had a major financial problem	1	2	3	4	5
E26. You got married	1	2	3	4	5
E27. Your partner hurt your children physically	1	2	3	4	5
E28. You attempted suicide	1	2	3	4	5
E29. You were convicted of an offence	1	2	3	4	5
E30. You were bleeding and thought you might miscarry	1	2	3	4	5
E31. You started a new job	1	2	3	4	5
E32. You had a test to see if your baby was abnormal	1	2	3	4	5
E33. You had a result on a test that suggested your baby might not be normal	1	2	3	4	5
E34. You were told that you were going to have twins	1	2	3	4	5
E35. You heard that something that had happened might be harmful to the baby	1	2	3	4	5
E36. You tried to have an abortion	1	2	3	4	5
E37. You took an examination	1	2	3	4	5
E38. Your partner was emotionally cruel to you	1	2	3	4	5
E39. Your partner was emotionally cruel to your children	1	2	3	4	5
E40. Your house or car was burgled	1	2	3	4	5
E41. You had an accident	1	2	3	4	5

E42. Having a baby is an important event. How much did this affect you?

a lot 1 moderately 2

Mildly 3 not at all 4

E43. a) Is there anything else which is not on the list which has concerned you or required additional effort from you to cope since becoming pregnant?

Yes 1 No 2 **If no, go to Section F**

If yes, b) please describe : ....

c) How did this affect you?

a lot 1 moderately 2

Mildly 3 not at all 4

SECTION F:LOOKING AFTER YOUR BABY

F1. When did you come home from the maternity ward?

days after baby was born

(if same day put 00, if never went into hospital put 77)

F2. Since coming home with my baby I have found it:

easier than expected 1

about as difficult as I expected 2

more difficult than I expected 3

does not apply (baby not home yet) 4

F3. How many hours sleep do you get altogether now?

		0 - 1 hours	2 - 3 hours	4 - 5 hours	6 - 7 hours	more than 7 hours
a)	during an average night	1	2	3	4	5
b)	during an average day	1	2	3	4	5

F4. Do you feel that you are getting enough sleep?

Yes 1 No 2

F5. Do you manage to go out (eg. shopping, visiting friends) now you have the baby?

yes, as much as I always did 1

yes, but a bit less now 2

very much less now 3

no, not at all 4

F6. What is the <u>present</u> job situation of yourself and your partner?

	(i) Yourself	(ii) Your partner
working for an employer full-time (more than 30 hours a week)	01	01
working for an employer part-time (one hour or more a week)	02	02
self-employed, employing other people	03	03
self-employed, not employing other people	04	04
on paid maternity leave	05	

	(i) Yourself	(ii) Your partner
on a government employment or training scheme	06	06
waiting to start a job already accepted	07	07
unemployed and looking for a job	08	08
at school or in other full-time education	09	09
unable to work because of long-term sickness or disability	10	10
retired from paid work	11	11
looking after the home or family	12	12
don't have a partner		77
other (please describe)	13	13

....

If you are not doing paid work at present then go to F9 below.

F7. How many weeks old was your baby when you began to work? weeks

F8. How many hours per week do you work? hours

F9. Who regularly looks after your baby when you are not there?
(Please answer for each person regularly involved.)

		No	Yes	If yes, give hours per week	and	Age of baby when this began (in weeks)
i)	partner	1	2			
ii)	baby's grandparent	1	2			
iii)	other relative	1	2			
iv)	friend/neighbour	1	2			
v)	paid person outside your home (e.g. child minder)	1	2			
vi)	paid person in your home (eg. nanny, baby sitter)	1	2			
vii)	day nursery (creche)	1	2			
viii)	other (please describe)	1	2			

....

If you have had no other children, go to F11 below.

F10. Before you had this baby had you ever used any of the following for child care?

		Yes	No
a)	partner	1	2
b)	baby's grandparent	1	2
c)	other relative	1	2
d)	friend/neighbour	1	2
e)	childminder (outside baby's home)	1	2
f)	babysitter, nanny (in baby's home)	1	2
g)	day nursery	1	2
h)	other (please describe)	1	2

....

If you are currently in paid work go to F12

F11. a) If you are not now in paid work do you think you will start work before your baby is one year old?

yes	1
no	2
don't know	3

If no, or don't know go to F12

If yes,

b) how old do you think your baby will be when you start work? months

F11. c) What arrangements have you made about looking after your baby when you begin work?

		Yes	No	Don't know
i)	partner	1	2	9
ii)	baby's grandparent	1	2	9
iii)	other relative	1	2	9
iv)	friend/neighbour	1	2	9
v)	childminder (outside baby's home)	1	2	9
vi)	babysitter, nanny (in baby's home)	1	2	9
vii)	day nursery	1	2	9
viii)	other (please describe)	1	2	9

....

F12. Whether or not you go back to work, are you planning to use any form of these in the next few months?

		Yes	No	Don't know
a)	paid help in your home (nanny, baby sitter)	1	2	9
b)	child minder (outside your home)	1	2	9
c)	other (please describe)	1	2	9

....

SECTION G:SUPPORT AND HELP

The following statements are about the help and support you have. You may have already answered questions like this during your pregnancy. Please do so again.
This is so that we can see how having a baby may have changed the way you feel.

		This is exactly how I feel	This is often how I feel	This is how I sometimes feel	I never feel this way	Have no partner
G1.	I have no one to share my feelings with	1	2	3	4	
G2.	My partner provides the emotional support I need	1	2	3	4	7
G3.	There are other mothers with whom I can share my experiences	1	2	3	4	
G4.	I believe in moments of difficulty my neighbours would help me	1	2	3	4	
G5.	I'm worried that my partner might leave me	1	2	3	4	7
G6.	There is always someone with whom I can share my happiness and excitement about my baby	1	2	3	4	
G7.	If I feel tired I can rely on my partner to take over	1	2	3	4	7
G8.	If I was in financial difficulty I know my family would help if they could	1	2	3	4	
G9.	If I was in financial difficulty I know my friends would help if they could	1	2	3	4	
G10.	If all else fails I know the state will support and assist me	1	2	3	4	

G11. How much help would you say you have had with the following since having your b aby?

		A lot of help	Some help	Hardly any help	No help at all
a)	shopping	1	2	3	4
b)	cleaning the home	1	2	3	4
c)	preparing meals	1	2	3	4
d)	washing up	1	2	3	4
e)	changing nappies	1	2	3	4
f)	washing the clothes	1	2	3	4
g)	other tasks (please describe)	1	2	3	4

....

G12. Do you feel you received:

too much help	1	the right amount of help	2	too little help	3

G13. Who has helped with the housework or the baby since your baby was born?

		**Yes, helped a lot**	**Yes, helped a bit**	**No, help at all**	**Not able/ available to help**	**No such person**
a)	partner	1	2	3	4	7
b)	your mother	1	2	3	4	7
c)	other relative	1	2	3	4	7
d)	neighbour	1	2	3	4	7
e)	friend	1	2	3	4	7
f)	paid help	1	2	3	4	7
g)	other (please describe)	1	2	3	4	7

....

SECTION H

H1. Please put the date of completing this questionnaire:

day month year

199

H2. Please give the date of birth of:

a) Yourself

day month year

19

b) Your baby

day month year

199

If you smoke, please remember to send back an empty cigarette packet.

Space for any comments you might like to make:

VERY MANY THANKS FOR ALL YOUR HELP

Please remember, because this is strictly confidential, the people who look at this booklet will not know your name. They will be unable to give you any help or contact anyone after reading what you have written. If you feel you need advice, please feel free to contact our special hot line (Bristol 256260 during office hours).Alternatively your General Practitioner should be able to advise you. If you would like to talk to someone about how you are feeling, contact your health visitor, or Mothers for Mothers, Tel: (Bristol) 232360 between 9 30am and 2 30pm.

When completed, return the questionnaire to:

Dr. Jean Golding,
Children of the Nineties - ALSPAC,
Institute of Child Health,
24, Tyndall Avenue,
Bristol.
BS8 1BR.
