# Supplementary material for: Breastfeeding, prenatal depression and children’s IQ and behaviour: a test of a moderation model
Source: BMC Pregnancy Childbirth. 2021 Jan 18;21:62. doi: 10.1186/s12884-020-03520-8 (PMC7814604; doi:10.1186/s12884-020-03520-8)
Supplement: Supplementary file 2 — Additional file 2:. ques-m04-your-pregnancy. Questionnaire data collected at 32 gestational weeks including maternal anxiety and depression and socio-demographic variables such as maternal and paternal education used in this study. [file 12884_2020_3520_MOESM2_ESM.pdf]

© University of Bristol

YOUR PREGNANCY

This questionnaire asks about how you are now feeling and somequestions about your background, and about your plans and preparations for the baby.

Your answers are confidential. Your name will not be on the questionnaire and none of the doctors or nurses you see will know your answers.

Please answer all the questions you can. If there are any you cannot answer or do not wish to answer that is fine. Just leave them blank

THANK YOU VERY MUCH FOR YOUR HELP

06/02/92

Recycled Paper

FILLING IN THIS BOOKLET

Most of the questions can be answered by ticking the box beside the right answer

For example

How many times have you been to the supermarket in the past week?

None 1   1 2   2-6 3   7 or more 4

↓

This means you went to the supermarket once in the past week

Sometimes there are questions with if in front of them

For example

a) Have you been to the supermarket today?

Yes 1   No 2

↓

This means you didn't go to the supermarket and you don't need to answer the next question

b) **If yes**, did you buy any carrots?

Yes 1   No 2

In general, though, each question needs an answer

In some questions you may be asked to describe something
It would be helpful if you wrote as clearly as possible

The small numbers in the squares are for office use only

SECTION A:PLANS AND EXPECTATIONS

Information about pregnancy

A1 a) Before you became pregnant this time did you read a lot about pregnancy and becoming a parent?

yes, a lot	1
yes, some	2
yes, a little	3
no, I didn't want to	4
no, I didn't have time	5
no, I didn't need to	6

b) Do you have friends or relatives who have children with whom you can discuss your pregnancy?

yes, many	1
yes, some	2
no	3

A2 How would you describe the knowledge you have about having a baby?

		**I knew nothing**	**I knew a little**	**I knew quite a lot**
i)	before you became pregnant this time	1	2	3
		**I know nothing**	**I know a little**	**I know quite a lot**
ii)	now	1	2	3

A3 a) Have you attended childbirth preparation classes in this pregnancy?

yes	1
no, but intend to	2
no, and don't intend to	3
haven't decided	4

b) Did you attend classes in a previous pregnancy?

Yes 1	No 2	Never been pregnant before 7

A4 How much do you want to know about what might happen during labour?

		**Yes**	**No**
i)	I'd rather not know anything	1	2
ii)	I just want to know the basics	1	2
iii)	I want to know most things but not things that will upset or worry me	1	2
iv)	I'm happy to let the staff decide how much I ought to know	1	2
v)	I want to know as much as possible	1	2

A5 Which of these options would you prefer ideally?

- the most pain-free labour that drugs/ epidural can give me 1
- the minimum amount of drugs to keep the pain manageable 2
- no pain killers at all 3
- don't have any opinion 9
- other (please describe) 4

A6 Would you like someone you know (husband/partner/mother/friend) with you at all times throughout your labour?

- yes, I want this very much 1
- yes, I would quite like this 2
- I don't mind 3
- no, I would prefer not to have this 4
- no, I definitely do not want this 5

A7 Assuming that there are no complications, who do you think should make the decisions about your labour?

(tick one only)

- doctors 1
- midwives 2
- doctors and midwives 3
- doctors, midwives and me together 4
- me 5
- midwives and me together 6
- don't know 9

A8 How important is it to you that giving birth will be a wonderful experience?

- very important 1
- quite important 2
- not very important 3
- not at all important 4
- I don't know 9

A9 a) Do you intend to start work after you have the baby?

Yes 1 No 2 **If no** go to B1

If yes,

b) about how old do you expect the baby will be when you go back to work?

- less than 6 weeks 1
- 6 weeks - 5 months 2
- 6 months - 12 months 3
- over 12 months 4

c) Have you decided what sort of child care you will have?

Yes 1 No 2

d) **If <u>yes</u>**, what sort of child care do you expect to use?

		**Yes**	**No**	**Don't know**
i)	nanny/childminder in your home	1	2	9
ii)	childminder outside your home	1	2	9
iii)	partner	1	2	9
iv)	family	1	2	9
v)	nursery/creche	1	2	9
vi)	other (please describe)	1	2	9

SECTION B:YOUR PRESENT HEALTH

B1 How would you describe your health **in the last two weeks:**

always fit and well 1

usually fit and well 2

sometimes unwell 3

often unwell 4

always unwell 5

B2 **In the last 3 months** have you had any of the following:

		Yes, in last 3 months	No, not in last 3 months	Don't know
a)	nausea	1	2	9
b)	vomiting	1	2	9
c)	diarrhoea	1	2	9
d)	vaginal bleeding	1	2	9
e)	jaundice	1	2	9
f)	urinary infection	1	2	9
g)	a cold	1	2	9
h)	influenza (flu)	1	2	9
i)	rubella (german measles)	1	2	9
j)	thrush (candida)	1	2	9
k)	genital herpes	1	2	9
l)	other infection (please describe)	1	2	9
m)	injury or shock to you (please describe)	1	2	9
n)	sugar in urine	1	2	9
o)	x-ray	1	2	9
p)	amniocentesis (amnio)	1	2	9
q)	chorionic villus sampling (CVS)	1	2	9
r)	AFP test (spina bifida test)	1	2	9
s)	ultrasound scan	1	2	9
t)	headache	1	2	9
u)	backache	1	2	9
v)	varicose veins	1	2	9

B3 a) Have you been admitted to hospital in the last 3 months?

Yes 1 No 2 **If <u>no</u>**, go to B4

If <u>yes</u>, give reason for each admission:

b) Reason	Date admitted	Number of days stayed
i)	/ /199	
ii)	/ /199	
iii)	/ /199	
iv)	/ /199	
v)	/ /199	

B4 In the last 3 months have you used any medicines, pills or ointments for the following:

Medicine, pills, ointment for:	Yes, in last 3 months	No, not in last 3 months	Don't know
a) nausea	1	2	9
b) heartburn	1	2	9
c) vomiting	1	2	9
d) anxiety	1	2	9
e) infection	1	2	9
f) migraine	1	2	9
g) difficulty going to sleep	1	2	9
h) pain	1	2	9
i) allergies	1	2	9
j) skin condition	1	2	9
k) bleeding	1	2	9
l) depression	1	2	9
m) piles	1	2	9
n) constipation	1	2	9
o) cough	1	2	9
p) other reason (please describe)	1	2	9

B5 In the last three months have you been taking any of the following?

	Yes	No
a) iron	1	2
b) zinc	1	2
c) calcium	1	2
d) folic acid/folate	1	2
e) vitamins (please describe)	1	2

....

f) other supplements or diet foods (please describe) 1

....

B6 Do you ever take homeopathic medicines?

Yes often 1 Yes sometimes 2 No 3

If yes, please list any you have taken this pregnancy: ....

....

B7 Please indicate how often you have taken the following pills in the last three months

		Every day	Most days	Some- times	Not at all
i)	aspirin	1	2	3	4
ii)	paracetamol	1	2	3	4
iii)	codeine/anadin	1	2	3	4
iv)	mogadon, or other sleeping tablets	1	2	3	4
v)	valium, or other tranquillisers	1	2	3	4

B8 Please describe all pills, medicines and ointments you have taken or used **in the past 3 months**, including those listed above

	What did you take: (give exact name if you can)	About how many days did you take or use it?	How many weeks pregnant were you?
1			
2			
3			
4			
5			
6			
7			
8			
9			
10			

Check **Have you included the contraceptive pill, iron tablets, laxatives, vitamins, sleeping tablets, aspirin, cough mixture, pain killers, indigestion tablets, herbal medicine?**

If you need more room continue on extra page

SECTION C:YOUR DIET

C1 We are interested in your diet How many times nowadays do you eat:

`

		Never or rarely	Once in 2 weeks	1 - 3 times a week	4 - 7 times a week	More than once a day
a)	Sausages, Burgers	1	2	3	4	5
b)	Pies, Pasties (pork pie, steak/meat pie etc)	1	2	3	4	5
c)	Meat (beef, lamb, pork, ham, bacon etc)	1	2	3	4	5
d)	Poultry (chicken, turkey etc)	1	2	3	4	5
e)	Liver, liver pate, kidney, heart	1	2	3	4	5
f)	White fish (cod, haddock, plaice, fish fingers etc)	1	2	3	4	5
g)	Other fish (pilchards, sardines, mackerel, tuna, herring, kippers, trout, salmon etc)	1	2	3	4	5
h)	Shellfish (prawns, crab, cockles, mussels etc)	1	2	3	4	5
i)	Eggs, quiche	1	2	3	4	5
j)	Cheese	1	2	3	4	5
k)	Pizza	1	2	3	4	5
l)	Chips	1	2	3	4	5
m)	Roast potatoes (cooked in fat)	1	2	3	4	5
n)	Boiled, mashed, jacket potatoes	1	2	3	4	5
o)	Rice (boiled)	1	2	3	4	5
p)	Pasta (egspaghetti, Pot Noodles, lasagna)	1	2	3	4	5
q)	Crisps	1	2	3	4	5
r)	Fried foods (egfried fish, eggs, bacon, chops etc)	1	2	3	4	5

C2 Do you eat the fat on meat?

yes, all of it	1
yes, some of it	2
no	3
never eat meat	4

C3 How many times a week nowadays do you eat:

		Never or rarely	Once in 2 weeks	1 - 3 times a week	4 - 7 times a week	More than once a day
a)	Baked beans	1	2	3	4	5
b)	Peas, sweetcorn, broad beans	1	2	3	4	5
c)	Cabbage, brussel sprouts, kale and other green leafy vegetables	1	2	3	4	5
d)	Other green vegetables (cauliflower, runner beans, leeks etc.)	1	2	3	4	5
e)	Carrots	1	2	3	4	5
f)	Other root vegetables (turnip, swede, parsnip etc.)	1	2	3	4	5
g)	Salad (lettuce, tomato, cucumber etc.)	1	2	3	4	5
h)	Fresh fruit (apple, pear, banana, orange, bunch of grapes etc.)	1	2	3	4	5
i)	Tinned juice (including tomato juice)	1	2	3	4	5
j)	Pure juice not in tin	1	2	3	4	5
k)	Pudding (e.g. fruit pie, crumble, cheesecake, milk pudding, mousse, gateaux)	1	2	3	4	5
l)	Oat cereals (e.g. porridge, Ready Brek, muesli)	1	2	3	4	5
m)	Wholegrain or bran cereals (e.g. All Bran, Bran Flakes, Weetabix, Wheatflakes, Fruit & Fibre)	1	2	3	4	5
n)	Other cereals (e.g. Corn-flakes, Rice Krispies, Special K, Frosties)	1	2	3	4	5
o)	Cakes or buns (fruit cake, sponge, teacake, buns, doughnut, flapjack, scone, custard tart, cream cake etc.)	1	2	3	4	5
p)	Crispbreads (Ryvita, crackerbread etc.)	1	2	3	4	5
q)	Biscuits (digestive, shortcake, Hob Nobs, Rich Tea, Nice, Marie, chocolate biscuits, Penguin, Club, Kit Kat etc.)	1	2	3	4	5
r)	Chocolate bars (Mars, Twix, Wispa, Bounty, Creme Egg etc.)	1	2	3	4	5
s)	Pulses - dried peas, beans, lentils, chick peas	1	2	3	4	5
t)	Nuts, nut roast	1	2	3	4	5
u)	Bean Curd (e.g. Tofu, miso)	1	2	3	4	5
v)	Tahini	1	2	3	4	5
w)	Soya 'Meat', T V P , Vegeburgers	1	2	3	4	5

		Never or rarely	Once in 2 weeks	1 - 3 times a week	4 - 7 times a week	More than once a day
x)	Chocolate (dairy milk or plain, nut, fruit filled etc.)	1	2	3	4	5
y)	Sweets (peppermints, boiled sweets, toffees etc.)	1	2	3	4	5

C4 When you have a soft drink, how often do you choose low calorie or diet drinks?

always	1
sometimes	2
not at all	3
don't drink soft drinks	7

C5 How many pieces of bread, rolls or chappatis do you eat on a usual day ?

less than 1 1   1-2 2   3-4 3   5 or more 4

C6 How many times in a month do you eat take-away foods for your main meal?

never or rarely	1
1 - 2	2
3 - 4	3
5 - 9	4
10 or more	5

C7 What types of bread do you eat most days?

		Yes	No
a)	white bread	1	2
b)	brown/granary bread	1	2
c)	wholemeal bread	1	2
d)	chappatis, nan bread	1	2
e)	don't usually eat any bread	1	2

C8 What sort of fat do you mainly use:

		(i) On bread or vegetables		(ii) For frying	
		Yes	No	Yes	No
a)	Butter, Ghee, Dripping Lard, solid cooking fat	1	2	1	2
b)	Hard or soft margarine e.g. Blue Band, Stork, supermarket own brand	1	2	1	2
c)	Polyunsaturated margarine e.g. Flora, sunflower, Vitalite	1	2	1	2
d)	Low fat spread e g Outline, Delight, St Ivel Gold	1	2	1	2
e)	Sunflower, soya, corn, olive oil	1	2	1	2
f)	Other vegetable oil	1	2	1	2

g) Other (please describe) 1 2 1 2

C9 How many slices of bread (or rolls) spread with fat do you eat each day?(include bought sandwiches) slices

C10 What type(s) of milk do you use?

	Yes usually	Yes sometimes	No not at all
a) Full fat (silver or gold top)	1	2	3
b) Semi Skimmed (red stripe)	1	2	3
c) Skimmed (blue stripe)	1	2	3
d) Sterilised	1	2	3
e) Dried milk	1	2	3
f) Goat/sheep milk	1	2	3
g) Soya milk	1	2	3
h) Other (please describe)	1	2	3

C11 How often do you have milk:

	Yes usually	Yes sometimes	No not at all
a) In tea	1	2	3
b) In coffee	1	2	3
c) On breakfast cereal	1	2	3
d) As pudding (custard,rice)	1	2	3
e) To drink on its own	1	2	3
f) As a milky drink (Horlicks, cocoa, all milk coffee)	1	2	3

C12 a) How many cups of tea do you drink in a day? (do not include herbal teas) cups

b) How many spoons of sugar in each cup? spoons

c) How many of the cups of tea you drink each day are decaffeinated? cups

d) How many cups of coffee do you drink in a day? cups

e) How many spoons of sugar in each cup? spoons

f) How many of the cups of coffee you drink each day are decaffeinated? cups

g) How many of the cups of coffee you drink each day are made using real coffee (ienot instant)? cups

h) How many of these are decaffeinated? cups

C13 a) How many drinks of cola do you have **in a week**? drinks

b) How many of these drinks are decaffeinated? drinks

C14 a) Do you drink herbal teas at all?

yes, often 1 yes, occasionally 2 no, not at all 3

If no, go to C15

If yes,

b) how many cups/mugs of herbal teas have you drunk in the past week? cups/mugs

c) Please list the types of herbal teas you have drunk in the past 3 months:

C15 Do you buy organic foods?

		Yes, usually organic	Yes, sometimes organic	No, never organic
a)	fruit	1	2	3
b)	vegetables	1	2	3
c)	meat	1	2	3
d)	other (please describe)	1	2	3

C16 Apart from herbal teas, are there any other health foods (whether or not bought from a health food shop) that you often eat or drink?

Yes 1 No 2

If yes, please describe below:

C17 a) Have you been on a diet this pregnancy?

Yes 1 No 2

If yes, please describe the type of diet:

C17 b) Apart from this pregnancy have you ever gone on a diet to lose weight?

Yes 1 No 2

If yes,

c) how often?

1-2 1 3-5 2 6-10 3 more than 10 times 4

d) how long do your diets usually last?

under 1 month 1 1-3 months 2 more than 3 months 3

C18 a) Are you, or have you ever been a vegetarian?

yes, I am now 1 yes, in past not now 2 no, never 3

If yes,

b) how many years of your life have you been vegetarian?

years (If less than one year put 00)

C19 a) Are you, or have you ever been, a vegan (ie do not eat meat, poultry, fish, eggs, butter, milk or cheese)?

yes, I am now 1 yes, in past not now 2 no, never 3

If yes,

b) how many years of your life have you been vegan?

years (If less than one year put 00)

		**Yes, most of the time**	**Yes, occasionally**	**No, not at all**
C20	Do you now feel you've put on too much weight?	1	2	3
C21	Do you feel uncomfort-able seeing your body in the mirror?	1	2	3
C22	Have you had a strong desire to lose weight at any time during this pregnancy?	1	2	3
C23	Do you feel dissatisfied about your shape?	1	2	3
C24	Have you experienced any loss of control over eating during this pregnancy?	1	2	3
C25	Are you concerned about losing any extra weight you've gained in this pregnancy?	1	2	3

C26 How many days in the past month have you drunk the equivalent of 2 pints of beer, 4 glasses of wine or 4 pub measures of spirit?

everyday	5	more than 10 days	4
5-10 days	3	3-4 days	2
1-2 days	1	none	0

C27 At present how much of the following do you usually drink in a day:

**At present**	**Weekday**	**Weekend day**
a) beer or lager (half-pints)		
b) wine (glasses)		
c) spirits (pub-measures)		
d) other alcoholic drinks (pub measures)		

SECTION D:YOUR OWN CHILDHOOD

Please indicate if any of the following events happened to you before you were 17 and how much it affected you.

**Before you were 17:**		**Yes affected me a lot**	**Yes moderately affected**	**Yes mildly affected**	**Yes but did not affect me**	**No did not happen**
D1	Your parent died	1	2	3	4	5
D2	A brother or sister died	1	2	3	4	5
D3	A relative died	1	2	3	4	5
D4	A friend died	1	2	3	4	5
D5	A parent had a serious illness	1	2	3	4	5
D6	A parent was in hospital	1	2	3	4	5
D7	You had a serious physical illness	1	2	3	4	5
D8	You were in hospital	1	2	3	4	5
D9	Brother or sister had a serious illness	1	2	3	4	5
D10	Brother or sister was in hospital	1	2	3	4	5
D11	A parent had a serious accident	1	2	3	4	5
D12	You had a serious accident	1	2	3	4	5
D13	Brother or sister had a serious accident	1	2	3	4	5
D14	You acquired a physical deformity	1	2	3	4	5
D15	You became pregnant	1	2	3	4	5
D16	A parent was imprisoned	1	2	3	4	5
D17	A parent was physically cruel to you	1	2	3	4	5
D18	Your parents separated	1	2	3	4	5

**Before you were 17:**		**Yes affected me a lot**	**Yes moderately affected**	**Yes mildly affected**	**Yes but did not affect me**	**No did not happen**
D19	Your parents divorced	1	2	3	4	5
D20	A parent remarried	1	2	3	4	5
D21	A parent was emotionally cruel to you	1	2	3	4	5
D22	Your parents had serious arguments	1	2	3	4	5
D23	You were sexually abused	1	2	3	4	5
D24	A parent was mentally ill	1	2	3	4	5
D25	You discovered you	1	2	3	4	5

were adopted

Before you were 17:	Yes affected me a lot	Yes moderately affected	Yes mildly affected	Yes but did not affect me	No did not happen
D26 Your family moved to a new district	1	2	3	4	5
D27 You were in trouble with the police	1	2	3	4	5
D28 You were expelled or suspended from school	1	2	3	4	5
D29 You failed an important exam	1	2	3	4	5
D30 Your family's financial circumstances got worse	1	2	3	4	5
D31 You acquired a step-brother or stepsister	1	2	3	4	5
D32 Other important happening (please tick & describe)	1	2	3	4	5

D33 How many schools did you attend between the ages of 5 and 16?

D34 Looking back would you call your childhood happy? Please indicate for each age range:

	Yes very happy	Yes moderately happy	Not really happy	No quite unhappy	No very unhappy	Can't remember
i) 0-5 years	1	2	3	4	5	6
ii) 6-11 years	1	2	3	4	5	6
iii) 12-15 years	1	2	3	4	5	6

D35 How many brothers and sisters did you have:

Brothers Sisters

a) older than you

b) younger than you

c) did you have a twin?

yes, twin brother 1

yes, twin sister 2

no 3

If you had a twin sister:

i) were you identical twins?

yes 1 no 2 not sure 3

ii) did you usually dress alike?

yes, usually 1 yes, sometimes 2 no, not at all 3

SECTION E:YOUR ENVIRONMENT AND LIFESTYLE

E1 a) Are you living in the same home that you were in at the start of your pregnancy?

Yes 1 No 2

b) **If no**, how many times have you moved?

c) Have you been homeless at any time during this pregnancy?

Yes 1 No 2

d) Have we sent this questionnaire to your correct address?

Yes 1 No 2

If no, please telephone Bristol 256260 or send a card with your new address, quoting your contact number

e) Are you intending to move house in the near future?

Yes 1 No 2

If yes, please let us know your new address on the back cover

E2 Please indicate how often during the day you are in a room or enclosed place where other people are smoking:

	**(i) Weekdays**	**(ii) Weekends**
all the time	1	1
more than 5 hours	2	2
3-5 hours	3	3
1-2 hours	4	4
less than 1 hour	5	5
not at all	6	6

E3 How many cigarettes per day are you yourself smoking at the moment cigarettes

E4 a) Are you currently in paid work?

Yes 1 No 2

If yes, go to Question E5

b) Have you worked at all during this pregnancy?

Yes 1 No 2 **If no**, go to E6

c) What date did you stop work? / /19

d) What was the main reason?

ill health	1
tiredness	2
company rules	3
to prepare for the baby	4
other (please describe)	5

e) Are you now on paid maternity leave?

Yes 1 No 2

E5 a) If you are working, how many hours per week do you work? hours

b) Do you do shift work?

Yes 1 No 2

c) **If yes**, does this include night shift?

Yes 1 No 2

E6 Which of the following statements best applied to you, in the last 3 months and now:

	Very energetic	Quite energetic	Lacking in energy
a) in the last 3 months	1	2	3
b) nowadays	1	2	3

E6 c) Compared with other pregnant women of your age, would you consider yourself to be:

much more active	1
somewhat more active	2
about the same	3
somewhat less active	4
much less active	5

d) Nowadays, at least once a week do you engage in any regular activity like brisk walking, gardening, housework, jogging, cycling, etc long enough to work up a sweat?

Yes 1 No 2

e) **If yes,** how many hours a week: hours

E7 In a normal day now, whether at home or not, do you:

	Yes often	Yes sometimes	No not at all
a) lift and carry young children	1	2	3
b) lift and carry heavy objects (more than 10kg or 20lb)	1	2	3
c) bend and stoop	1	2	3
d) have rest periods	1	2	3
e) use vibrating machinery	1	2	3

E8 How difficult at the moment do you find it to afford these items:

	Very difficult	Fairly difficult	Slightly difficult	Not difficult
a) Food	1	2	3	4
b) Clothing	1	2	3	4
c) Heating	1	2	3	4
d) Rent or mortgage	1	2	3	4
e) Things you will	1	2	3	4

SECTION F:YOUR FEELINGS

The questions in this section ask you about your feelings and the way you behave nowadays Please indicate the way you feel

		Very often	Often	Not very often	Never
F1	Do you feel upset for no obvious reason?	1	2	3	4
F2	Do you get troubled by dizziness or shortness of breath?	1	2	3	4
F3	Have you felt as though you might faint?	1	2	3	4
F4	Do you feel sick or have indigestion?	1	2	3	4
F5	Do you feel that life is too much effort?	1	2	3	4
F6	Do you feel uneasy and restless?	1	2	3	4
F7	Do you feel tingling or prickling sensations in your body, arms or legs?	1	2	3	4
F8	Do you regret much of your past behaviour?	1	2	3	4
F9	Do you sometimes feel panicky?	1	2	3	4
F10	Do you find that you have little or no appetite?	1	2	3	4
F11	Do you wake unusually early in the morning?	1	2	3	4
F12	Do you worry a lot?	1	2	3	4
F13	Do you feel tired or exhausted?	1	2	3	4
F14	Do you experience long periods of sadness?	1	2	3	4
F15	Do you feel strung-up inside?	1	2	3	4
F16	Can you get off to sleep alright?	1	2	3	4
F17	Do you ever have the feeling you are going to pieces?	1	2	3	4
F18	Do you often have excessive sweating or fluttering of the heart?	1	2	3	4
F19	Do you find yourself needing to cry?	1	2	3	4
F20	Do you have bad dreams which upset you when you wake up?	1	2	3	4
F21	Do you lose the ability to feel sympathy for others?	1	2	3	4
F22	Can you think as quickly as you used	1	2	3	4

to?

		Very often	Often	Not very often	Never
F23	Do you have to make a special effort to face up to a crisis or difficulty?	1	2	3	4

Your feelings in the past week

F24 I have been able to laugh and see the funny side of things:

As much as I always could	1
Not quite so much now	2
Definitely not so much now	3
Not at all	4

F25 I have looked forward with enjoyment to things:

As much as I ever did	1
Rather less than I used to	2
Definitely less than I used to	3
Hardly at all	4

Your feelings in the past week

F26 I have blamed myself unnecessarily when things went wrong:

Yes, most of the time	1
Yes, some of the time	2
Not very often	3
No, never	4

F27 I have been anxious or worried for no good reason:

No, not at all	1
Hardly ever	2
Yes, sometimes	3
Yes, often	4

F28 I have felt scared or panicky for no very good reason:

Yes, quite a lot	1
Yes, sometimes	2
No, not much	3
No, not at all	4

F29 Things have been getting on top of me:

Yes, most of the time	1
Yes, sometimes	2
No, hardly ever	3
No, not at all	4

In the past week

F30 I have been so unhappy that I have had difficulty sleeping:

Yes, most of the time 1

Yes, sometimes 2

Not very often 3

No, not at all 4

F31 I have felt sad or miserable:

Yes, most of the time 1

Yes, quite often 2

Not very often 3

No, not at all 4

F32 I have been so unhappy that I have been crying:

Yes, most of the time 1

Yes, quite often 2

Only occasionally 3

No, never 4

F33 The thought of harming myself has occurred to me:

Yes, quite often 1

Sometimes 2

Hardly ever 3

Never 4

SECTION G:INFANT FEEDING

Below are some attitudes about infant feeding often expressed by mothers
What do you feel?

		Strongly agree	Agree	Unsure	Disagree	Strongly disagree
G1	Breast-feeding stops a mother from having the freedom to do what she wants	1	2	3	4	5
G2	Breast-feeding gives the mother a special relationship with her baby	1	2	3	4	5
G3	Bottle-feeding allows the father to share the child more	1	2	3	4	5
G4	Breast milk is better for the baby	1	2	3	4	5
G5	Bottle-feeding is more convenient for the mother	1	2	3	4	5
G6	A mother who does not breast feed is inferior	1	2	3	4	5
G7	Breast-feeding is difficult	1	2	3	4	5

G8 How are you going to feed your baby:

		Breast	Bottle	Both	Uncertain
a)	in the first week	1	2	3	4
b)	in the first month	1	2	3	4
c)	in the next 3 months	1	2	3	4

G9 How does your partner want you to feed the baby?

don't know 1

no strong feelings 2

undecided 3 don't have a partner 7

wants me to breast feed 4

wants me to bottle feed 5

G10 Were you breast fed as a baby?

Yes 1 No 2 Don't know 9

SECTION H:EDUCATION AND OCCUPATION

H1 What educational qualifications do you, your partner, your mother, and your father have? Please tick all that apply

		(i) Your self	(ii) Your partner	(iii) Your mother*	(iv) Your father*
a)	CSE or GCSE (D, E, F or G)	1	1	1	1
b)	O-level or GCSE (A, B or C)	1	1	1	1
c)	A-level	1	1	1	1
d)	Qualifications in shorthand/ typing/or other skills, e g hairdressing	1	1	1	1
e)	Apprenticeship	1	1	1	1
f)	State enrolled nurse	1	1	1	1
g)	State registered nurse	1	1	1	1
h)	City & Guilds intermediate technical	1	1	1	1
i)	City & Guilds final technical	1	1	1	1
j)	City & Guilds full technical	1	1	1	1
k)	Teaching qualification	1	1	1	1
l)	University degree	1	1	1	1
m)	No qualifications	1	1	1	1
n)	Qualifications not known	1	1	1	1
o)	Not applicable, no such person	1	1	1	1
p)	Other (please describe)	1	1	1	1

[* by this we mean the mother figure or father figure who was mostly responsible for bringing you up]

H2 What is the present employment situation of yourself and your partner? Please tick all that apply

		(i) Yourself	(ii) Your partner
a)	Working for an employer full-time (more than 30 hours a week)	1	1
b)	Working for an employer part-time (one hour or more a week)	1	1
c)	Self-employed, employing other people	1	1
d)	Self-employed, not employing other people	1	1
e)	On a government employment or training scheme	1	1
f)	Waiting to start a job already accepted	1	1
g)	Unemployed and looking for a job	1	1
h)	At school or in other full-time education	1	1
i)	Unable to work because of long-term sickness or disability	1	1

j) Retired from paid work 1 1

k) Looking after the home or family 1 1

l) Other (please describe) 1 1

H3 If your partner is not currently in paid employment w hen did his last job end?

Date your partner stopped working / /19

(If you are unsure, put an approximate date, e.g March 1988)

The questions below ask about your current occupation and that of your partner

H4 As far as you can, please describe the actual job, occupation, trade or profession (Use precise terms such as radio mechanic, woodworking machinist, tool-room foreman If the occupation is known by a special name, please use that name If in H M Forces, give the rank in addition to the actual job Please also describe the type of industry or service given: i.e. Give details of what is made, materials used, or services given)

a) **Your present job or last main job**

i) Actual job, occupation, trade or profession

ii) Hours worked per week:

iii) Please tick which of the following apply to you:

foreman 1

manager 2

supervisor 3

leading hand 4

self-employed 5

none of these 6

iv) Type of industry or service given (main things done in job):

……….

b) **Your partner** - present job or last main job

i) Do you currently have a partner?

Yes 1 No 2

If no, go to H5

ii) **If yes,** what is/was his actual job, occupation, trade or profession?

H4 b) ii) Hours worked per week:

iii) Please tick which of the following apply to him:

foreman 1

manager 2

supervisor 3

leading hand 4

self-employed 5

none of these 6

not known 9

iv) Type of industry or service given (main things done in job):

v) Is he in contact with particular fumes or chemicals in his job?

always 1	often 2	sometimes 3
rarely 4	never 5	don't know 9

If yes, please describe:

H5 a) **The main job your mother or mother figure did at around the time you left school** (Please put HW if she was a housewife)

i) Actual job, occupation, trade or profession:

……………………………………………………………..

ii) Type of industry or service given (main things done in job):

……………………………………………………………..

H5 b) How old was your natural mother when you were born? (If you don't know, put 99) years

	Yes	No	Don't know
c) Is your natural mother still alive?	1	2	9

H6 a) **The main job your father or father figure did at around the time you left school** (If not known put NK)

i) Actual job, occupation, trade or profession:

……………………………………………………………..

ii) Please tick which of the following applied to him:

foreman	1
manager	2
supervisor	3
leading hand	4
self-employed	5
none of these	6

iii) Type of Industry or service given (main things done in job):

……………………………………………………………..

b) How old was your natural father when you years were born? (If you don't know, put 99)

	Yes	No	Don't know
c) Is your natural father still alive?	1	2	9

Problems

H7 Do you think you have been unfairly/unjustly treated in the last 12 months because of:

	Yes often	Yes sometimes	No not at all
a) your sex	1	2	3
b) your skin colour	1	2	3
c) the way you dress	1	2	3

d)	your family background	1	2	3
e)	the way you speak	1	2	3
		**Yes often**	**Yes sometimes**	**No not at all**
f)	your religion	1	2	3
g)	other (please describe)	1	2	3

H8 How would you describe the race or ethnic group of yourself, your partner and your parents?

	**(i) Yourself**	**(ii) Partner**	**(iii) Your mother***	**(iv) Your father***
white	01	01	01	01
black/Caribbean	02	02	02	02
black/African	03	03	03	03
black/other (please describe below)	04	04	04	04
Indian	05	05	05	05
Pakistani	06	06	06	06
Bangladeshi	07	07	07	07
Chinese	08	08	08	08
any other ethnic group (please describe)	09	09	09	09

(*by this we mean the mother or father figure who was mostly responsible for bringing you up)

SECTION I:BEING A PARENT

Below are a number of statements about how some people think a parent should behave with a baby Please indicate how much you agree with them

		Yes, I agree	I'm unsure but probably agree	I'm unsure but probably disagree	No, I disagree
I1	Babies should be picked up whenever they cry	1	2	3	4
I2	It is important to develop a regular pattern of feeding and sleeping with a baby	1	2	3	4
I3	Babies should be fed whenever they are hungry	1	2	3	4
I4	Babies need to be stimulated if they are to develop well	1	2	3	4
I5	Babies need quiet secure surroundings and should not be disturbed too much	1	2	3	4
I6	Parents need to adapt their lives to the baby's demands	1	2	3	4
I7	A baby should fit into its parents routine	1	2	3	4
I8	Babies should be left to develop naturally	1	2	3	4
I9	Talking, to even a very young baby, is important	1	2	3	4
I10	Cuddling a baby is very important	1	2	3	4

I11 What is the youngest age at which you think it is alright for a mother to leave her child regularly in the care of another person during the day?

0 - 5 months 1 6 - 11 months 2 1 - 2 years 3

3 - 4 years 4 5 years or more 5 never 6

don't know 9

SECTION J

J1 Please put the date of completing this part of the questionnaire:

day month year

199

J2 Please give your date of birth:

day month year

19

Thank you for your help so far

These next pages are concerned with early sexual experience

IF YOU WOULD RATHER NOT ANSWER THEM, WE QUITE UNDERSTANDJUST STOP NOW AND SEND THE QUESTIONNAIRE BACK AS USUAL

But it is possible that whether or not such events have taken place they may be a vital clue in understanding some of the problems we are trying to solve - even though they may appear to be unconnected. If you feel you can help, we would be very grateful

SECTION K

As we are growing up we all have sexual experiences These are a normal part of development and learning Some people also have unwanted experiences to which they do not agree These experiences can be important and may affect how you feel about yourself, your partner and your baby Below are questions which ask about your sexual experiences from childhood until the present time

K1 Did anyone ever purposefully expose/flash themselves to you before you were 16?

Yes, happened once only 1

Yes, happened more than once 2

No, did not happen 3

If yes,

	(i) Who was involved?		(ii) **If yes**, did you want this to happen with this person?		
	**No**	**Yes**	**No**	**Yes**	**Unsure**
a) boy friend	1	2	1	2	9
b) girl friend	1	2	1	2	9
c) parent or parent figure	1	2	1	2	9
d) brother or sister	1	2	1	2	9
e) other relative	1	2	1	2	9
f) family friend	1	2	1	2	9
g) stranger	1	2	1	2	9
h) other person (please describe)	1	2	1	2	9

iii) how old were you when this first happened: years

K2 Did anyone masturbate in front of you before you were 16?

Yes, happened once only 1

Yes, happened more than once 2

No, did not happen 3

If yes,

	(i) Who was involved?		(ii) If yes, did you want this to happen with this person?		
	**No**	**Yes**	**No**	**Yes**	**Unsure**
a) boy friend	1	2	1	2	9
b) girl friend	1	2	1	2	9
c) parent or parent figure	1	2	1	2	9
d) brother or sister	1	2	1	2	9
e) other relative	1	2	1	2	9
f) family friend	1	2	1	2	9
g) stranger	1	2	1	2	9
h) other person (please describe)	1	2	1	2	9

iii) how old were you when this first happened: years

K3 Did anyone ever touch or fondle your body, including your breast or genitals, or attempt to arouse you sexually before you were 16?

Yes, happened once only 1

Yes, happened more than once 2

No, did not happen 3

If yes,

		(i) Who was involved?		(ii) If yes, did you want this to happen with this person?		
		**No**	**Yes**	**No**	**Yes**	**Unsure**
a)	boy friend	1	2	1	2	9
b)	girl friend	1	2	1	2	9
c)	parent or parent figure	1	2	1	2	9
d)	brother or sister	1	2	1	2	9
e)	other relative	1	2	1	2	9
f)	family friend	1	2	1	2	9
g)	stranger	1	2	1	2	9
h) (	other person please describe)	1	2	1	2	9

iii) how old were you when this first happened: years

K4 Did anyone try to have you arouse them, or touch their body in a sexual way before you were 16?

Yes, happened once only 1

Yes, happened more than once 2

No, did not happen 3

If yes,

		**(i) Who was involved?**		**(ii) If yes, did you want this to happen with this person?**		
		**No**	**Yes**	**No**	**Yes**	**Unsure**
a)	boy friend	1	2	1	2	9
b)	girl friend	1	2	1	2	9
c)	parent or parent figure	1	2	1	2	9
d)	brother or sister	1	2	1	2	9
e)	other relative	1	2	1	2	9
f)	family friend	1	2	1	2	9
g)	stranger	1	2	1	2	9
h)	other person please describe)	1	2	1	2	9

iii) how old were you when this first happened: years

K5 Did anybody rub their genitals against your body in a sexual way before you were 16?

Yes, happened once only 1

Yes, happened more than once 2

No, did not happen 3

If <u>yes</u>,

	(i) Who was involved?		(ii) If yes, did you want this to happen with this person?		
	**No**	**Yes**	**No**	**Yes**	**Unsure**
a) boy friend	1	2	1	2	9
b) girl friend	1	2	1	2	9
c) parent or parent figure	1	2	1	2	9
d) brother or sister	1	2	1	2	9
e) other relative	1	2	1	2	9
f) family friend	1	2	1	2	9
g) stranger	1	2	1	2	9
h) other person (please describe)	1	2	1	2	9

iii) how old were you when this first happened: years

K6 Did anyone have sexual intercourse with you before you were 16?

Yes, happened once only 1

Yes, happened more than once 2

No, did not happen 3

If <u>yes</u>,

	(i) Who was involved?		(ii) If yes, did you want this to happen with this person?		
	**No**	**Yes**	**No**	**Yes**	**Unsure**
a) boy friend	1	2	1	2	9
b) girl friend	1	2	1	2	9
c) parent or parent figure	1	2	1	2	9
d) brother or sister	1	2	1	2	9
e) other relative	1	2	1	2	9
f) family friend	1	2	1	2	9
g) stranger	1	2	1	2	9
h) other person (please describe)	1	2	1	2	9

iii) how old were you when this first happened: years

K7 Did anyone ever try to put their penis into your mouth before you were 16?

Yes, happened once only 1

Yes, happened more than once 2

No, did not happen 3

If yes,

		(i) Who was involved?		(ii) If yes, did you want this to happen with this person?		
		**No**	**Yes**	**No**	**Yes**	**Unsure**
a)	boy friend	1	2	1	2	9
b)	father or father figure	1	2	1	2	9
c)	brother	1	2	1	2	9
d)	other relative	1	2	1	2	9
e)	family friend	1	2	1	2	9
f)	stranger	1	2	1	2	9
g)	other person (please describe)	1	2	1	2	9

iii) how old were you when this first happened: years

Thank you for answering these questions which we realise may be difficult to answer If there are any comments you'd like to make please write them below

VERY MANY THANKS FOR ALL YOUR HELP

When completed, put in the envelope provided and either bring to the clinic or post to:

Dr Jean Golding,
Children of the Nineties - ALSPAC,
Institute of Child Health,
24 Tyndall Avenue,
Bristol
BS8 1BR

Please remember, because this is strictly confidential, the people who look at this booklet will not know your name They will be unable to give you any help or contact anyone after reading what you have written If you feel you need advice, please feel free to contact our special information line (Bristol 256260 during office hours) Alternatively your Midwife or General Practitioner should be able to advise you
