# Supplementary material for: Breastfeeding, prenatal depression and children’s IQ and behaviour: a test of a moderation model
Source: BMC Pregnancy Childbirth. 2021 Jan 18;21:62. doi: 10.1186/s12884-020-03520-8 (PMC7814604; doi:10.1186/s12884-020-03520-8)
Supplement: Supplementary file 3 — Additional file 3:. ques-m03-having a baby. Questionnaire data collected at 18 gestational weeks including sociodemographic variables used in this study such as smoking, quality of home environment (HOME) and maternal age. [file 12884_2020_3520_MOESM3_ESM.pdf]

© University of Bristol

HAVING A BABY

This questionnaire asks about how you have been feeling so far in your pregnancy.

Your answers are confidential. Your name will not be on the questionnaire and none of the doctors or nurses you see will know your answers.

Please answer all the questions you can. If there are any you cannot answer or do not wish to answer that is fine. Just leave them blank.

THANK YOU VERY MUCH FOR YOUR HELP

06/02/92

Recycled Paper

FILLING IN THIS BOOKLET

Most of the questions can be answered by ticking the box beside the right answer.

For example

How many times have you been to the supermarket in the past week?

None 1   1✓ 2   2-6 3   7 or more 4

↓

This means you went to the supermarket once in the past week

Sometimes there are questions with if in front of them.

For example

a) Have you been to the supermarket today?

Yes 1   No✓ 2

↓

This means you didn't go to the supermarket and you don't need to answer the next question

b) **If yes**, did you buy any carrots?

Yes 1   No 2

In general, though, each question needs an answer.

In some questions you may be asked to describe something.
It would be helpful if you wrote as clearly as possible.

The small numbers in the squares are for office use only.

SECTION A:YOUR PREVIOUS PREGNANCIES

A1. Have you ever been pregnant before?

Yes 1 No 2 **If no**, go to Section B

If yes,

A2. a) How many times have you been pregnant altogether before this time?

b) Is this the first pregnancy with your present partner?

Yes 1 No 2 Am not sure 9

A3. a) How many children still living, of your own do you have?

b) Do they all live with you?

Yes 1 No 2 Don't have children 7

A4. a) Have you ever had any miscarriages?

Yes 1 No 2

b) **If yes**, how many times have you miscarried?

A5. a) Have you ever had any abortions or terminations?

Yes 1 No 2

b) **If yes, how** many ?

A6. a) Have you ever had a stillborn baby ?

Yes 1 No 2

b) **If yes**, how many?

A7. a) Have you ever had any babies who were born alive but died later?

Yes 1 No 2

If yes, please describe:

b) how many?

c) what caused their death? ....

....

....

d) how old were they when they died? ....

A8. Were any of your babies under 5lb 8oz (2500 grammes) at birth?

Yes 1 No 2 Don't know 9

A9. a) Were any of your babies born more than 3 weeks early?

Yes 1 No 2 Don't know 9

A9. b) Have you ever had a caesarean section?

Yes 1 No 2 Don't know 9

A10. How old were you when you became pregnant for the very first time?

years

A11. a) What was the outcome of the last pregnancy before this pregnancy?

miscarriage 1

abortion or termination 2

stillbirth 3

liveborn baby that died 4

liveborn baby still alive 5

other (please describe) 6

....

b) Please give the date of your last birth/miscarriage/abortion or termination before this pregnancy:

day month year

19

c) Did you breast feed your last baby?

Yes 1 No 2 Have not had a baby 7

d) **If <u>yes</u>**, for how long?

under 1 month	1
1-3 months	2
more than 3 months	3

SECTION B:YOUR HEALTH

B1. How would you describe your health:

		Always fit & well	Usually fit & well	Sometimes unwell	Often unwell	Always unwell
a)	up to the time of your present pregnancy	1	2	3	4	5
b)	in the first months of this pregnancy	1	2	3	4	5
c)	in the last two weeks	1	2	3	4	5

B2. **During this pregnancy** so far have you had any of the following:

		Yes,in 1st 3 months	Yes in period 4th month to now	No, not at all	Don't know
a)	nausea	1	2	4	9
b)	vomiting	1	2	4	9
c)	diarrhoea	1	2	4	9
d)	vaginal bleeding	1	2	4	9
e)	jaundice	1	2	4	9
f)	urinary infection	1	2	4	9
g)	influenza	1	2	4	9
h)	rubella (german measles)	1	2	4	9
j)	thrush (candida)	1	2	4	9
k)	genital herpes	1	2	4	9
l)	other infection (please describe)	1	2	4	9

....

m) injury or shock to you (please describe)

....

n)	sugar in urine	1	2	4	9
o)	x-ray	1	2	4	9
p)	amniocentesis	1	2	4	9
q)	chorionic villus sampling (CVS)	1	2	4	9
r)	AFP test (spina bifida test)	1	2	4	9
s)	ultrasound scan	1	2	4	9

B3. a) Have you been admitted to hospital during this pregnancy?

Yes 1 No 2 **If no**, go to B4

b) **If yes**, give reason for each admission:

	Reason	Date admitted	Number of days stayed
i)	....	.../.../199...	....
ii)	....	.../.../199...	....
iii)	....	.../.../199...	....
iv)	....	.../.../199...	....
v)	....	.../.../199...	....

B4. During this pregnancy have you ever taken any medicines, pills or used ointment or suppositories for the following:

**Medicines, pills or ointments for:**		**Yes, taken in 1st 3 months of pregnancy**	**Yes, taken later in pregnancy**	**No, not at all**
a)	nausea	1	2	4
b)	heartburn	1	2	4
c)	vomiting	1	2	4
d)	anxiety	1	2	4
e)	infection	1	2	4
f)	migraine	1	2	4
g)	difficulty going to sleep	1	2	4
h)	pain	1	2	4
i)	allergies	1	2	4
j)	skin condition	1	2	4
k)	bleeding	1	2	4
l)	depression	1	2	4
m)	piles	1	2	4
n)	constipation	1	2	4
o)	cough	1	2	4
p)	other reason (please describe)	1	2	4

....

....

B5. During this pregnancy have you been taking any of the following?

		**Yes**	**No**
a)	iron	1	2
b)	zinc	1	2
c)	calcium	1	2
d)	folic acid/folate	1	2
e)	vitamins (please name brand)	1	2

....

f)	other supplements or diet foods (please describe)	1	2

....

B6. a) Do you ever take homeopathic medicines?

Yes often 1	Yes sometimes 2	No 3

b) **If <u>yes</u>**, please describe: ....

B7. a) Please indicate how often you have taken the following pills during this pregnancy.

		**Every day**	**Most days**	**Some-times**	**Not at all**
i)	aspirin	1	2	3	4
ii)	paracetamol	1	2	3	4
iii)	codeine/anadin	1	2	3	4
iv)	mogadon, or other sleeping tablets	1	2	3	4
v)	valium, or other tranquillisers	1	2	3	4

B7. b) Please name any pills, medicines and ointments you have taken or used **in the past 3 months**.

	**What did you take:**	**About how many days did you take or use it?**	**How many weeks pregnant were you?**
1.	....	....	....
2.	....	....	....
3.	....	....	....
4.	....	....	....
5.	....	....	....
6.	....	....	....
7.	....	....	....
8.	....	....	....
9.	....	....	....
10.	....	....	....
11.	....	....	....
12.	....	....	....
13.	....	....	....
14.	....	....	....
15.	....	....	....

Check **Have you included the contraceptive pill, iron tablets, laxatives, vitamins, sleeping tablets, aspirin, cough mixture, pain killers, herbal medicine?**

SECTION C:YOUR REACTIONS TO BECOMING A PARENT

C1. a) Were you deliberately trying to get pregnant this time?

Yes 1 No 2 **If no**, go to Question C2.

If yes,

b) for how long had you been trying?

under 6 months 1

6-11 months 2

1-2 years 3

3 years or more 4

C2. How would you describe your reaction when you **first found you were pregnant this time?**

(tick one only)

overjoyed 1

pleased 2

mixed feelings 3

not happy 4

very unhappy 5

no particular feelings 6

C3. a) Does being a mother mean giving up something that is important to you?

yes, a great deal 1

yes, quite a lot 2

not really 3

definitely not 4

don't know 9

Please add any extra comments you wish to make:

....

....

....

C3. b) Does becoming a mother give you new opportunities and interests?

yes, a great deal 1

yes, quite a lot 2

not really 3

definitely not 4

don't know 9

Please add any extra comments you wish to make:

....

....

....

C4. How do you feel about your pregnancy **now**?

overjoyed	1
pleased	2
mixed feelings	3
not happy	4
very unhappy	5
no particular feelings	6

C5. How do you think your partner feels about your pregnancy?

overjoyed	1
pleased	2
mixed feelings	3
have no partner	7
not happy	4
very unhappy	5
no particular feelings	6

C6. How has your partner reacted to you since you became pregnant this time?

	**When he first knew**	**Now**
supportive	1	1
indifferent	2	2
resentful	3	3
have no partner	7	7
other (please describe)	4	4

....

C7. Do you want a boy or girl?

boy	1
girl	2
don't mind	3

C8. Do you think your partner wants a boy or girl?

boy	1
girl	2
doesn't mind	3
he doesn't know I'm pregnant yet	4
I have no partner	7

SECTION D:YOUR FEELINGS

The questions in this section ask you about your feelings and behaviour.

Please indicate the way you feel at this stage in your pregnancy.

		Very often	Often	Not very often	Never
D1.	Do you feel upset for no obvious reason?	1	2	3	4
D2.	Do you get troubled by dizziness or shortness of breath?	1	2	3	4
D3 .	Have you felt as though you might faint?	1	2	3	4
D4 .	Do you feel sick or have indigestion?	1	2	3	4
D5.	Do you feel that life is too much effort?	1	2	3	4
D6.	Do you feel uneasy and restless?	1	2	3	4
D7.	Do you feel tingling or prickling sensations in your body, arms or legs?	1	2	3	4
D8.	Do you regret much of your past behaviour?	1	2	3	4
D9.	Do you sometimes feel panicky?	1	2	3	4
D10.	Do you find that you have little or no appetite?	1	2	3	4
D11.	Do you wake unusually early in the morning?	1	2	3	4
D12.	Do you worry a lot?	1	2	3	4
D13.	Do you feel tired or exhausted?	1	2	3	4
D14.	Do you experience long periods of sadness?	1	2	3	4
D15.	Do you feel strung-up inside?	1	2	3	4
D16.	Can you get off to sleep alright?	1	2	3	4
D17.	Do you ever have the feeling you are going to pieces?	1	2	3	4
D18.	Do you often have excessive sweating or fluttering of the heart?	1	2	3	4
D19.	Do you find yourself needing to cry?	1	2	3	4

	Very often	Often	Not very often	Never
D20. Do you have bad dreams which upset you when you wake up?	1	2	3	4
D21. Do you lose the ability to feel sympathy for others?	1	2	3	4
D22. Can you think quickly?	1	2	3	4
D23. Do you have to make a special effort to face up to a crisis or difficulty?	1	2	3	4

Your feelings in the past week.

D24. I have been able to laugh and see the funny side of things:

As much as I always could	1
Not quite so much now	2
Definitely not so much now	3
Not at all	4

D25. I have looked forward with enjoyment to things:

As much as I ever did	1
Rather less than I used to	2
Definitely less than I used to	3
Hardly at all	4

D26. I have blamed myself unnecessarily when things went wrong:

Yes, most of the time	1
Yes, some of the time	2
Not very often	3
No never	4

D27. I have been anxious or worried for no good reason:

No, not at all	1
Hardly ever	2
Yes, sometimes	3
Yes, often	4

D28. I have felt scared or panicky for no very good reason:

Yes, quite a lot	1
Yes, sometimes	2
No, not much	3
No, not at all	4

D29. Things have been getting on top of me:

Yes, most of the time	1
Yes, sometimes	2
No, hardly ever	3
No, not at all	4

D30. I have been so unhappy that I have had difficulty sleeping:

Yes, most of the time	1
Yes, sometimes	2
Not very often	3
No, not all	4

D31. I have felt sad or miserable:

Yes, most of the time	1
Yes, quite often	2
Not very often	3
No, not at all	4

D32. I have been so unhappy that I have been crying:

Yes, most of the time	1
Yes, quite often	2
Only occasionally	3
No, never	4

D33. The thought of harming myself has occurred to me:

Yes, quite often	1
Sometimes	2
Hardly ever	3
Never	4

D34. **Since you became pregnant** have you noticed any change in:

		**Yes, increased a lot**	**Yes, increased a little**	**No change a little**	**Yes decreased a lot**	**Yes decreased**
a)	How irritable you are	1	2	3	4	5
b)	How nervous you are	1	2	3	4	5
c)	How healthy you are	1	2	3	4	5
d)	How communicative you are	1	2	3	4	5
e)	How active you are	1	2	3	4	5
f)	How able you are to think and concentrate	1	2	3	4	5
g)	How physically	1	2	3	4	5

attractive you are

D35. How frequently **in the past few weeks** have you been:

		Always	Often	Sometimes	Rarely	Never
a)	Irritable	1	2	3	4	5
b)	Nervous	1	2	3	4	5
c)	Active	1	2	3	4	5
d)	In good health	1	2	3	4	5
e)	Communicative	1	2	3	4	5
f)	Able to think and concentrate	1	2	3	4	5
g)	Feeling attractive	1	2	3	4	5

IF YOU HAVE NO PARTNER, PLEASE PUT A LINE THROUGH QUESTIONS D36 AND D37, AND THEN GO TO QUESTION E1

D36. **Since becoming pregnant** have you noticed any change in **your partner** in any of the following?

		Yes, increased a lot	Yes, increased a little	No change a little	Yes decreased a lot	Yes decreased
a)	How irritable he is	1	2	3	4	5
b)	How nervous he is	1	2	3	4	5
c)	How healthy he is	1	2	3	4	5
d)	How communicative he is	1	2	3	4	5
e)	How active he is	1	2	3	4	5
f)	How able he is to think and concentrate	1	2	3	4	5
g)	How physically attractive he is	1	2	3	4	5

D37. How frequently **in the past few weeks** has he been:

		Always	Often	Sometimes	Rarely	Never
a)	Irritable	1	2	3	4	5
b)	Nervous	1	2	3	4	5
c)	Active	1	2	3	4	5
d)	In good health	1	2	3	4	5
e)	Communicative	1	2	3	4	5
f)	Able to think and concentrate	1	2	3	4	5

g)	Looking attractive	1	2	3	4	5

SECTION E:OCCUPATION

E1. a) Have you ever had a paid job?

Yes 1 No 2 → **If no**, go to E5

If yes,

b) Are you currently working? Yes 1 → **If yes**, go to E2.

No 2

If no,

c) What date did you stop work? ...../...../19.....

d) Why did you stop work at this time?

ill health/tiredness 1

looking after children/preparing for baby 2

made redundant 3

didn't like the job 4

moved house 5

other (please describe) 6 ....

e) Are you now on paid maternity leave?

Yes 1 No 2

E2. a) What is your present job? If you are not working, what was your most recent job?

....

b) Are/were you working:

full-time 1 part-time 2 casually 3

c) type of industry or service given:

....

E2. d) Do/did you do shift work?

Yes 1 No 2

e) **If yes**, does/did this include night shift

Yes 1 No 2

If you have not worked at all this pregnancy, go to E5.

E3. What is your job like: (If you are no longer working answer for your most recent job this pregnancy).

	**Yes, always**	**Yes, mostly**	**Some-times**	**Not very often**	**Never**
a) Do you enjoy your job?	1	2	3	4	5
b) Do you have problems at work?	1	2	3	4	5
c) Are the people at your work friendly?	1	2	3	4	5
d) Are the people at your work supportive?	1	2	3	4	5
e) Is it very noisy?	1	2	3	4	5

f) Do you work in a smoky atmosphere? 1 2 3 4 5

E4. a) About how long does/did it take you altogether to travel, to get to work each day?

hours minutes

E4. b) About how long does/did it take you altogether to travel, to get back from work each day?

hours minutes

E4. c) How do/did you travel to work?

By foot 1	By public transport 2	By bicycle 3
By car 4	Work at home 5	Other(please describe) 6

....

E5. In the year before this pregnancy, in the first months of this pregnancy, and now did/do you do any of the following (whether at home, at school, at work or elsewhere):

		**(i) In the year before this pregnancy**		**(ii) In the first 3 months of this pregnancy**		**(iii) From 4 months of this pregnancy until now**	
		**Yes**	**No**	**Yes**	**No**	**Yes**	**No**
a)	Did/do you use a VDU? television type screen)	1	2	1	2	1	2
b)	Are/were you mostly sitting	1	2	1	2	1	2
c)	Are/were you bending a lot	1	2	1	2	1	2
d)	Are/were you standing much of the time?	1	2	1	2	1	2
e)	Are/were you doing repetitive, boring tasks	1	2	1	2	1	2
f)	Did/does your job involve challenging and mentally demanding tasks?	1	2	1	2	1	2
g)	Are/were you using a lot of physical energy?	1	2	1	2	1	2
h)	In your job are/were you in contact with fumes or chemicals? please describe)	1	2	1	2	1	2

....
Please make sure you have answered each of the three columns]

SECTION F:RECENT EVENTS

Listed below are a number of events which may have brought changes in your life. Have any of these occurred **since you became pregnant**? If so, please assess how much effect it had on you.

**Since becoming pregnant:**		**Yes & affected me a lot**	**Yes, moderately affected**	**Yes, mildly affected**	**Yes, but did not affect me at all**	**No did not happen**
F1.	Your partner died	1	2	3	4	5
F2.	One of your children died	1	2	3	4	5
F3.	A friend or relative died	1	2	3	4	5
F4.	One of your children was ill	1	2	3	4	5
F5.	Your partner was ill	1	2	3	4	5
F6.	A friend or relative was ill	1	2	3	4	5
F7.	You were admitted to hospital	1	2	3	4	5
F8.	You were in trouble with the law	1	2	3	4	5
F9.	You were divorced	1	2	3	4	5
F10.	You found that your partner didn't want your child	1	2	3	4	5
F11.	You were very ill	1	2	3	4	5
F12.	Your partner lost his job	1	2	3	4	5
F13.	Your partner had problems at work	1	2	3	4	5
F14.	You had problems at work	1	2	3	4	5
F15.	You lost your job	1	2	3	4	5
F16.	Your partner went away	1	2	3	4	5
F17.	Your partner was in trouble with the law	1	2	3	4	5
F18.	You and your partner separated	1	2	3	4	5
F19.	Your income was reduced	1	2	3	4	5
F20.	You argued with your partner	1	2	3	4	5
F21.	You had arguments with your family or friends	1	2	3	4	5

Since becoming pregnant:		Yes & affected me a lot	Yes, moderately affected	Yes, mildly affected	Yes, but did not affect me at all	No did not happen
F22.	You moved house	1	2	3	4	5
F23.	Your partner hurt you physically	1	2	3	4	5
F24.	You became homeless	1	2	3	4	5
F25.	You had a major financial problem	1	2	3	4	5
F26.	You got married	1	2	3	4	5
F27.	Your partner hurt your children physically	1	2	3	4	5
F28.	You attempted suicide	1	2	3	4	5
F29.	You were convicted of an offence	1	2	3	4	5
F30.	You were bleeding and thought you might miscarry	1	2	3	4	5
F31.	You started a new job	1	2	3	4	5
F32.	You had a test to see if your baby was abnormal	1	2	3	4	5
F33.	You had a result on a test that suggested your baby might not be normal	1	2	3	4	5
F34.	You were told that you were going to have twins	1	2	3	4	5
F35.	You heard that something that had happened might be harmful to the baby	1	2	3	4	5
F36.	You tried to have an abortion	1	2	3	4	5
F37.	You took an examination	1	2	3	4	5
F38.	Your partner was emotionally cruel to you	1	2	3	4	5
F39.	Your partner was emotionally cruel to your children	1	2	3	4	5
F40.	Your house or car was burgled	1	2	3	4	5
F41.	You had an accident	1	2	3	4	5

F42. a) Is there anything else which is not on the list which has concerned you or required additional effort from you to cope since becoming pregnant?

Yes 1 No 2 → **If no** go to F43.

If yes, b) please describe :

....

F42. c) How did this affect you?

a lot 1 moderately 2 mildly 3 not at all 4

F43. Becoming pregnant is an important event. How did this affect you?

a lot 1 moderately 2 mildly 3 not at all 4

SECTION G:ACTIVITIES AND LIFESTYLE

G1. Which of the following statements best applied to you just before you became pregnant, in the early months and now.

		Very energetic	Quite energetic	Lacking in energy
a)	before you became pregnant	1	2	3
b)	in the first 3 months	1	2	3
c)	now	1	2	3

d) Compared with other pregnant women of your age, would you consider yourself to be:

much more active	1
somewhat more active	2
about the same	3
somewhat less active	4
much less active	5

e) Nowadays, at least once a week do you engage in any regular activity like brisk walking, gardening, housework, jogging, cycling, etc. long enough to work up a sweat?

Yes 1 No 2

If yes,

f) how many hours a week: hours

G2. In a normal day now, whether at home or not, how often do you:

		Often	Occasionally	Not at all
a)	lift and carry heavy objects (more than 10Kg.)	1	2	3
b)	lift and carry young children	1	2	3
c)	bend and stoop	1	2	3
d)	have rest periods	1	2	3
e)	use vibrating machinery	1	2	3

G3. a) Have you ever been a smoker?

Yes 1 No 2 → **If no**, please go to G4, on page 30.

If yes,

b) at what age did you start smoking regularly? years

G3. c) which of the following have you smoked regularly?

	Yes	No
cigarette	1	2
pipe	1	2
cigar	1	2
other	1	2

d) What was the maximum number of times a day you smoked?

30+ 30	25-29 25	20-24 20	15-19 15
10-14 10	5-9 05	1-4 01	0 00

G3. e) Have you now stopped smoking?

Yes 1 No 2

If <u>yes</u>, how long ago? years months

G3. f) Did you smoke regularly at any of the following times in the last 9 months?

	**Before pregnancy**	**First 3 months of pregnancy**	**Last 2 weeks**
No	1	1	1
Yes, cigarettes	2	2	2
Yes, cigars	3	3	3
Yes, pipe	4	4	4
Yes, other (please describe)	5	5	5

....

g) how many times per day did you smoke -

i) **just before you became pregnant**

per day

30+ 30	25-29 25	20-24 20	15-19 15
10-14 10	5-9 05	1-4 01	0 00

ii) **in the first 3 months of your pregnancy**

per day

30+ 30	25-29 25	20-24 20	15-19 15
10-14 10	5-9 05	1-4 01	0 00

iii) **in the last 2 weeks?**

per day

30+ 30	25-29 25	20-24 20	15-19 15
10-14 10	5-9 05	1-4 01	0 00

G3. h) What brand and type of cigarette or tobacco do/did you usually smoke?

(i) brand: ....

(ii) type: filtered 1 unfiltered 2 roll-your-own 3 pipe/cigar 4

Please send us an empty packet/carton of the brand you usually smoke.

G4. a) Did your mother ever smoke?

Yes 1 No 2 Don't know 9

i) **If yes**, did she smoke when she was expecting you?

Yes 1 No 2 Don't know 9

b) Did your father ever smoke?

Yes 1 No 2 Don't know 9

G5. a) Does your partner smoke?

No 1 → **If no**, or don't have a partner, go to G6 on page 31.

Yes, cigarettes 2

Yes, cigars 3

Yes, pipe 4

Yes, other (please describe) 5 ....

Don't have a partner 7 → Go to G6, on page 31.

If yes,

b) about how many times per day does your partner smoke at the moment?

30+ 30	25-29 25	20-24 20	15-19 15
10-14 10	5-9 05	1-4 01	don't know 99

c) what brand, and type of cigarette/tobacco does your partner smoke?

i) brand: ....

ii) type:filtered 1 unfiltered 2 roll-your-own 3 pipe/cigar 4

d) at what age did your partner start smoking? years don't know 99

G6. a) Apart from yourself and your partner, are there any other members of your household who smoke?

Yes 1 No 2

b) **If yes**, how many: ....

G7. How often did you smoke marijuana/grass/cannabis/ ganja -

	**Every day**	**2-4 times a week**	**Once a week**	**Less than once a week**	**Not at all**
a) In the 6 months before you conceived	1	2	3	4	5
b) In the first 3 months of pregnancy	1	2	3	4	5
c) Between 3 months and now	1	2	3	4	5

G8. How often have you taken the following during this pregnancy:

		Nearly every day	At least once a week	At least once a month	Not at all
a)	amphetamines	1	2	3	4
b)	barbiturates	1	2	3	4
c)	crack	1	2	3	4
d)	cocaine	1	2	3	4
e)	heroin	1	2	3	4
f)	methadone	1	2	3	4
g)	ecstasy	1	2	3	4
h)	other (please describe)	1	2	3	4

....

G9. How often have you drunk alcoholic drinks? Please indicate for each of the following times:

		Never	Less than 1 glass* a week	At least 1 glass* a week	1-2 glasses* every day	At least 3-9 glasses* every day	At least 10 glasses* every day
a)	Before this pregnancy	1	2	3	4	5	6
b)	1st 3 months of this pregnancy	1	2	3	4	5	6
c)	At around the time you first felt baby move	1	2	3	4	5	6

(If you haven't felt the baby move yet, please answer for the last two weeks)

* [By glass we mean a pub measure of spirits, half a pint of lager or cider, a wine glass of wine, etc]

G10. How many days in the past month have you drunk the equivalent of 2 pints of beer, 4 glasses of wine or 4 pub measures of spirit?

everyday	5	more than 10 days	4
5-10 days	3	3-4 days	2
1-2 days	1	None	0

G11. Which is the alcoholic drink you have most often drunk during this pregnancy? (tick one only)

wine 1
beer/lager 2
sherry/port 3
gin/whisky/vodka/brandy 4
other (please describe) 5 ....
don't drink at all 7

G12. How would you describe your partner's alcohol drinking? Which of the following statements best applies:

Never drinks alcohol 1
Very occasionally (less than once a week) 2
Occasionally (at least once a week) 3
Drinks 1-2 glasses nearly every day 4
Drinks 3-9 glasses every day 5
Drinks at least 10 glasses a day 6
Don't have a partner 7
Don't know 9

G13. At present how much of the following do you usually drink in a day:

At present		Weekday	Weekend day
a)	ordinary tea (cups)	....	....
b)	decaffeinated tea (cups)	....	....
c)	coffee (cups)	....	....
d)	decaffeinated coffee (cups)	....	....
e)	beer or lager (half-pints)	....	....
f)	wine (glasses)	....	....
g)	spirits (pub-measures)	....	....
h)	cola/pepsi (cans)	....	....
i)	decaffeinated cola/pepsi cans	.......….	....
j)	other alcoholic drinks (pub measures)	....	....
k)	milk (glasses)	....	....
l)	other drinks (please describe)	....	....

....

G14. a) Have you **ever** actually made yourself sick (vomit) because you wanted to lose weight or because you had eaten too much?

Yes 1 No 2

b) If yes, how often have you made yourself vomit **during this pregnancy**?

not at all 5 once 1 2-4 times 25-14 times 3 15 or more times 4

G15. a) Have you **ever** taken laxatives because you wanted to lose weight or because you had eaten too much?

Yes 1 No 2

G15. b) If yes, how often have you done so **during this pregnancy**?

not at all 5 once 1 2-4 times 25-14 times 3 15 or more times 4

OVER THE PAST FOUR WEEKS (28 DAYS)

**G16.**		**Not at all**	**Yes occasionally**	**Yes most of the time**
a)	Has thinking about your shape or weight interfered with your ability to concentrate on things?	1	2	3
b)	Have you been afraid that you may become fat?	1	2	3
c)	Have you felt fat?	1	2	3
d)	Have you had a strong desire to lose weight?	1	2	3
e)	Has your weight influenced how you think about yourself as a person?	1	2	3

G16.		Not at all	Yes occasionally	Yes most of the time
f)	Have you felt dissatisfied about your weight?	1	2	3
g)	Have you felt dissatisfied about your shape?	1	2	3
h)	Have you felt concerned about other people seeing you eat?	1	2	3
i)	Have you felt uncomfortable seeing your body in the mirror?	1	2	3
j)	Have you experienced a loss of control over eating?	1	2	3

IN THE THREE MONTHS BEFORE YOU BECAME PREGNANT

G17.		Not at all	Yes occasionally	Yes most of the time
a)	Did thinking about your shape or weight interfere with your ability to concentrate on things?	1	2	3
b)	Were you afraid that you might become fat?	1	2	3
c)	Did you feel fat?	1	2	3
d)	Did you have a strong desire to lose weight?	1	2	3
e)	Did your weight influence how you thought about yourself as a person?	1	2	3
f)	Did you feel dissatisfied about your weight?	1	2	3
g)	Did you feel dissatisfied about your shape?	1	2	3
h)	Did you feel concerned about other people seeing you eat?	1	2	3
i)	Did you feel uncomfortable seeing your body in the mirror?	1	2	3
j)	Did you experience a loss of control over eating?	1	2	3

G18. Which of the following statements describes best the way in which you travel nowadays?

usually walk everywhere	1
cycle mostly	2
usually get in a car	3
mostly use public transport	4

G19. How much do you do the following at present?

		More than 7 hours per week	2-6 hours per week	Less than one hour per week	Never
a)	jogging	1	2	3	4
b)	aerobics	1	2	3	4
c)	ante-natal exercises	1	2	3	4

		More than 7 hours per week	2-6 hours per week	Less than one hour per week	Never
d)	keep fit exercises	1	2	3	4
e)	yoga	1	2	3	4
f)	squash	1	2	3	4
g)	tennis/badminton	1	2	3	4
h)	swimming	1	2	3	4
i)	brisk walking	1	2	3	4
j)	weight training	1	2	3	4
k)	cycling	1	2	3	4
l)	other exercise (please describe)	1	2	3	4

....

....

SECTION H:ABOUT YOURSELF

In general:		Very like	Moderately like me	Moderately unlike me	Very unlike
H1.	I feel insecure when I say goodbye to people	1	2	3	4
H2.	I worry about the effect I have on other people	1	2	3	4
H3.	I avoid saying what I think for fear of being rejected	1	2	3	4
H4.	I feel uneasy meeting new people	1	2	3	4
H5.	If others knew the real me, they would not like me	1	2	3	4
H6.	I feel secure when I'm in a close relationship	1	2	3	4
H7.	I don't get angry with people for fear that I may hurt them	1	2	3	4
H8.	After a row with a friend, I feel uncomfortable until I have made peace	1	2	3	4
H9.	I am always aware of how other people feel	1	2	3	4
H10.	I worry about being criticised for things I have said or done	1	2	3	4
H11.	I always notice if someone doesn't respond to me	1	2	3	4
H12.	I worry about losing someone close to me	1	2	3	4
H13.	I feel that people generally like me	1	2	3	4
H14.	I will do something I don't want to do rather than offend or upset someone	1	2	3	4
H15.	I can only believe that some-thing I have done is good when someone tells me	1	2	3	4
H16.	I will go out of my way to please someone I am close to	1	2	3	4
H17.	I feel anxious when I say goodbye to people	1	2	3	4
H18.	I feel happy when someone compliments me	1	2	3	4
H19.	I fear that my feelings will overwhelm me	1	2	3	4
H20.	I can make other people feel happy	1	2	3	4
H21.	I find it hard to get angry with people	1	2	3	4
H22.	I worry about criticising people	1	2	3	4
H23.	If someone is critical of something I do, I feel bad	1	2	3	4

In general:	Very like	Moderately like me	Moderately unlike me	Very unlike
H24. If other people knew what I am really like, they would think less of me	1	2	3	4
H25. I always expect criticism	1	2	3	4
H26. I can never be really sure if someone is pleased with me	1	2	3	4
H27. I don't like people to really know me	1	2	3	4
H28. If someone upsets me, I am not able to put it easily out of my mind	1	2	3	4
H29. I feel others do not under-stand me	1	2	3	4
H30. I worry about what others think of me	1	2	3	4
H31. I don't feel happy unless people I know admire me	1	2	3	4
H32. I am never rude to anyone	1	2	3	4
H33. I worry about hurting the feelings of other people	1	2	3	4
H34. I feel hurt when someone is angry with me	1	2	3	4
H35. My value as a person depends enormously on what others think of me	1	2	3	4
H36. I care about what people feel about me	1	2	3	4

SECTION I

I1. Please put the date of completing this questionnaire:

day month year 199

I2. Please give your date of birth:

day month year 19

N.B. Have you remembered to enclose an empty cigarette packet?

Space for any comments you might like to make:

VERY MANY THANKS FOR ALL YOUR HELP

When completed, put in the envelope provided and either bring to the clinic or post to:

Dr. Jean Golding,
Children of the Nineties - ALSPAC,
Institute of Child Health,
24 Tyndall Avenue,
Bristol.
BS8 1BR.

Please remember, because this is strictly confidential, the people who look at this booklet will not know your name. They will be unable to give you any help or contact anyone after reading what you have written. If you feel you need advice, please feel free to contact our hotline (Bristol 256260, during office hours).Alternatively your General Practitioner should be able to advise you.
