# Supplementary material for: Breastfeeding, prenatal depression and children’s IQ and behaviour: a test of a moderation model
Source: BMC Pregnancy Childbirth. 2021 Jan 18;21:62. doi: 10.1186/s12884-020-03520-8 (PMC7814604; doi:10.1186/s12884-020-03520-8)
Supplement: Supplementary file 4 — Additional file 4:. ques-cb19-your-son-at-9. Questionnaire data collected when the child was 9 years old including the SDQ sub-scales used in this study. [file 12884_2020_3520_MOESM4_ESM.pdf]

Questionnaire No:

YOUR DAUGHTER AT 9

All answers are confidential

Chloe

Sophie and Robert

Marley and friends

25/01/01

This questionnaire is to be answered by the child's main carer.

To answer simply tick the box which is most accurate in your opinion.

Some questions are the same as those you have answered before. This is so that we can tell what changes have happened to you.

Please answer all questions if you can, even if they are similar.

If you do not want to answer a question or if it does not apply to you, put a line through it.

There are no good or bad answers. Just tell us what is true of your child as far as you know.

All answers are confidential

THANK YOU FOR YOUR HELP

SECTION A: SCHOOL

A1. a) Does she go to school?

Yes ☐ 1 No ☐ 2 →**If no, go to A17a on page 11**

b) How many different schools has she gone to?

☐☐ different schools

c) What types of school has she gone to?

		(i) 1st school	(ii) 2nd school	(iii) 3rd school
d)	infant school	1	1	1
e)	primary school	1	1	1
f)	private (fee paying)	1	1	1
g)	boarding school	1	1	1
h)	studies at home	1	1	1
i)	special school*	1	1	1
j)	not able to be taught	1	1	1

*If special school please describe type ....

....

DID YOU KNOW? ……

8,500 children spent half a day at Focus@7.
Brilliant, thank you. Help us to encourage 10,000
to come to Focus @ 9.

A1. k) Please describe reasons for child being at current school (tick all that apply):

i) It was the only available choice [ ] 1

ii) It was the best available [ ] 1

iii) There were medical reasons [ ] 1 → please describe ……………..

iv) There were psychological reasons [ ] 1 → please describe ……………..

v) Other (please tick and describe) [ ] 1

………………………………………………………………………………………

A2.

		24 hour clock hours	minutes	
a)	What time does school start ?	[ ][ ]	[ ][ ]	(e.g. 4 o'clock in the afternoon is 16.00)
b)	What time does school end ?	[ ][ ]	[ ][ ]	
c)	What time does the child leave school to go home?	[ ][ ]	[ ][ ]	

A3. Who takes her to and from school nowadays? (Tick all that apply)

		(i) Going every day	some days	(ii) Coming back every day	some days
a)	I take her	1	2	1	2
b)	My partner takes her	1	2	1	2
c)	A grandparent or adult relative	1	2	1	2
d)	An older child	1	2	1	2
e)	Childminder	1	2	1	2
f)	Other person	1	2	1	2
g)	Goes on her own	1	2	1	2

A4. How does she get to school?

		(i) Going		(ii) Coming back	
		**every day**	**some days**	**every day**	**some days**
a)	She walks	1	2	1	2
b)	She goes in a wheelchair	1	2	1	2
c)	By public transport	1	2	1	2
d)	School bus/ coach	1	2	1	2
e)	By car	1	2	1	2
f)	Rides bicycle	1	2	1	2
g)	Other (please tick & describe)	1	2	1	2

....

A5. a) How far away is the school?

less than ½ mile (1 km) away	1
½ - 1 mile (1-2 km) away	2
1 - 5 miles (2-8 km) away	3
more than 5 miles (8 km) away	4

b) How long does it take to get there in the morning? [ ][ ] minutes

A6. We would like to know what happens after school.

. a) Does she usually go straight home?

No 1 Yes 2 →**If yes, go to A6c on page 6**

A6. b) **If no**, where does she go?

		Every day	Some days	Never
i)	to a relative's home	1	2	3
ii)	to a friend's home	1	2	3
iii)	to a childminder	1	2	3
iv)	school club	1	2	3
v)	plays outside	1	2	3
vi)	other (please tick and describe)	1	2	3

....

c) If she goes straight home is an adult always there?

yes, always 1 yes, usually 2 yes, sometimes 3

no, hardly ever 4

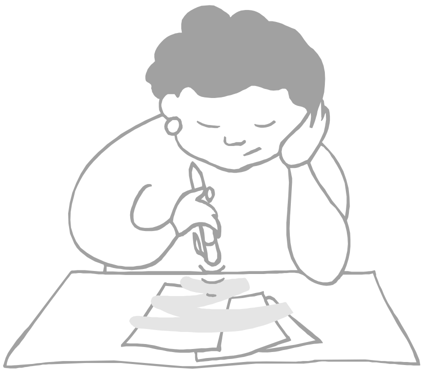

Some of these questions may seem familiar. We have asked similar ones before, but don't forget circumstances change for a lot of us over a period of time and your answers today may be different from last time!

A7. How does she feel about school?

		Always	Usually	Sometimes	Not at all
a)	She looks forward to seeing her teachers	1	2	3	4
b)	She enjoys school	1	2	3	4
c)	She is stimulated by it	1	2	3	4
d)	She is frightened by the teachers	1	2	3	4
e)	She is frightened by her school mates	1	2	3	4
f)	She is afraid of failure	1	2	3	4
g)	She seems bored by school	1	2	3	4
h)	She likes her school mates	1	2	3	4
i)	She looks forward to lessons	1	2	3	4

A8. At school how much do you think she likes:

		She likes it a lot	She quite likes it	She does not like it	Does not do this
a)	Science, natural history	1	2	3	4
b)	Maths	1	2	3	4
c)	English	1	2	3	4
d)	Games/PE	1	2	3	4
e)	Foreign language	1	2	3	4
f)	Art/painting	1	2	3	4
g)	Music	1	2	3	4
h)	Geography	1	2	3	4
i)	History	1	2	3	4

A8.

		She likes it a lot	She quite likes it	She does not like it
j)	Other topic (please tick & describe)	1	2	3

…………………………………………………………………………………..

A9. When she finishes school and returns home:

	**Does she:**	**Always**	**Usually**	**Sometimes**	**Never**
a)	Read, draw or play quietly by herself	1	2	3	4
b)	Watch TV or video	1	2	3	4
c)	Play with other children	1	2	3	4
d)	Use a computer	1	2	3	4
e)	Do schoolwork	1	2	3	4
f)	Listen to music	1	2	3	4
g)	Something else (please tick and describe)	1	2	3	4

…………………………………………………………………………………

A10. When you meet at home after school:

	**Does she:**	**Always**	**Usually**	**Sometimes**	**Never**
a)	Talk about the events of the day	1	2	3	4
b)	Ask about what has happened in your day	1	2	3	4

A11. a) Does she bring home things that she has done at school (e.g. painting)?

Yes [1] No [2] → **If no, go to A12 below**

b) **If yes**, how often are they put so that everyone can see them?

always [1] usually [2] sometimes [3] never [4]

A12. When she gets home from school how does she behave?

		Always	Usually	Sometimes	Never
a)	excited	1	2	3	4
b)	talkative	1	2	3	4
c)	withdrawn	1	2	3	4
d)	calm	1	2	3	4
e)	very active	1	2	3	4
f)	sleepy	1	2	3	4
g)	angry	1	2	3	4
h)	hungry	1	2	3	4
i)	tearful	1	2	3	4
j)	anxious	1	2	3	4
k)	affectionate	1	2	3	4
l)	tired	1	2	3	4
m)	in need of your support	1	2	3	4
n)	she avoids talking about school	1	2	3	4
o)	other (please tick and describe)	1	2	3	4

....

A13. a) Are you interested in what your child does at school?

Yes very [ ] 1 Yes mostly [ ] 2 No, not really [ ] 3

b) Are you happy with the teaching your daughter is getting at school?

Yes very [ ] 1 Yes mostly [ ] 2 No, not really [ ] 3

c) Are you happy with the progress your daughter is making at school?

Yes very [ ] 1 Yes mostly [ ] 2 No, not really [ ] 3

d) Are you happy with her behaviour at school?

Yes very [ ] 1 Yes mostly [ ] 2 No, not really [ ] 3

A14. Has she been identified as having any particular problems at school?

Yes [ ] 1 No [ ] 2 → **If no, go to A17 on page 11**

If yes, which problems? (tick all that apply)

a) disciplinary [ ] 1

b) learning [ ] 1

c) in relationships [ ] 1

d) emotional [ ] 1

e) other [ ] 1

f) Please describe each type of school problem:

…………………………………………………………………………………

…………………………………………………………………………………

…………………………………………………………………………………

A15. Have you been invited to the school to talk about any of these problems?

Yes [ ] 1 No [ ] 2

A16 a) Was the child investigated by a specialist for any of these problems?

Yes ☐1 No ☐2 → **If no, go to A17 below**

If yes, what was the result?

………………………………………………………………………………………..

………………………………………………………………………………………...

………………………………………………………………………………………...

b) Did this investigation result in extra help for the child?

Yes ☐1 No ☐2 → **If no, go to A17 below**

If yes, describe what:

…………………………………………………………………………………………

………………………………………………………………………………………...

………………………………………………………………………………………..

A17. a) Do you think that your study child has any particular talents?

Yes ☐1 No ☐2 → **If no, go to A18 below**

b) **If yes**, please describe …………………………………………………………

…………………………………………………………………………………………..

A18. Does your child show any interest in taking up any particular occupation when she is an adult?

Yes ☐1 No ☐2 → **If no, go to A19 below**

If yes, please describe…………………………………………………………………..

A19. Is there an occupation you really hope your child will choose?

Yes ☐1 No ☐2 → **If no, go to A20 on page 12**

If yes, please describe………………………………………………………………

A20. What sort of education do you hope your child will have? (tick just one)

- ☐ 1 the minimum – and leave school as soon as possible
- ☐ 2 to get some good GCSE's and then leave
- ☐ 3 to take at least one A level
- ☐ 4 to go to University
- ☐ 5 other (please tick and describe ……………………………………………)
- ☐ 6 don't really care

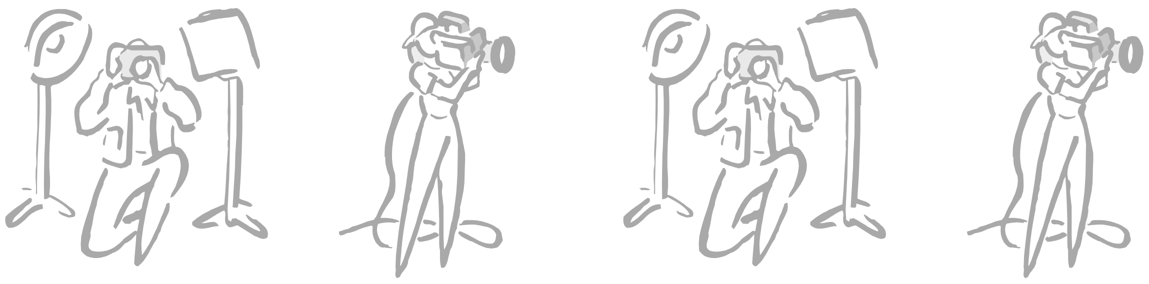

We try to use as many photographs as we can in your questionnaires, but everything you send us is used somewhere: in publicity material, in newsletters or to decorate our Focus clinics.

We love to receive pictures so please keep on sending them

SECTION B: DISCIPLINE & LIFESTYLE

B1. a) Are there rules in your home about what your study child can and cannot do?

No, not at all ☐1 | Yes for some things ☐2 | Yes, for many things ☐3

b) Does she refuse to do what she does not want to do?

Yes usually ☐1 | Yes sometimes ☐2 | No, not at all ☐3

	(i) I do	(ii) My husband/ partner does	(iii) Her teacher does	(iv) Someone else → please describe
c) Who has most control over your study child?	☐1	☐1	☐1	☐1 ………………..
d) Who usually tells her off?	☐1	☐1	☐1	☐1 ………………...
e) Who usually punishes her?	☐1	☐1	☐1	☐1 …………………

f) How often is she punished?

every day ☐1 | several times a week ☐2 | once or twice a week ☐3

once or twice a month ☐4 | rarely ☐5 | never ☐6

g) How often do you slap or hit her?

every day ☐1 | several times a week ☐2 | once or twice a week ☐3

once or twice a a month ☐4 | rarely ☐5 | never ☐6

B2. Has she ever run away from home?

Yes ☐1 | No, but has tried to ☐2 | No, but has thought of doing so ☐3

No, never ☐4 | Don't know ☐9

B3. Is there anyone that she is afraid of?

Yes 1 No 2 Don't know 9

If <u>no</u> or <u>don't know</u> go to B4 below

If <u>yes</u>, (please tick all that apply)

She is afraid of:

a) her father/step-father 1

b) her mother/step-mother 1

c) brother 1

d) sister 1

e) other relative 1

f) neighbour 1

g) teacher 1

h) other children 1

i) other, please describe 1

……………………………………………………………………………………………

B4. Is there anyone who particularly stands up for her?

Yes 1 No 2 Don't know 9

If <u>no</u> or <u>don't know</u> go to B5a on page 15

If <u>yes</u>, who is this? (please tick all that apply)

Stands up for her:

a) her father/step-father 1

b) her mother/step-mother 1

c) brother 1

Stands up for her:

B4.	d)	sister	1
	e)	other relative	1
	f)	neighbour	1
.	g)	teacher	1
	h)	other children	1
	i)	other, please tick & describe	1

…………………………………………………………………………………………

B5. a) On normal school days what time in the morning does your child usually wake up?

hours minutes

[ ][ ] [ ][ ] a.m.

b) On normal school days what time in the evening does your child usually go to sleep?

hours minutes

[ ][ ] [ ][ ] p.m.

c) On weekends what time in the morning does your child usually wake up?

hours minutes

[ ][ ] [ ][ ] a.m.

d) On weekends what time in the evening does your child usually go to sleep?

hours minutes

[ ][ ] [ ][ ] p.m.

B6. Have you ever been told that your child has:

		No	Yes	age at diagnosis	
a)	dyslexia	1	2 →		years
b)	dyspraxia	1	2 →		years
c)	dysgrafia	1	2 →		years
d)	dysortografia	1	2 →		years
e)	dyscalculia	1	2 →		years
f)	autism, Asperger's syndrome or autistic spectrum disorder	1	2 →		years
g)	other (please tick & describe)	1	2 →		years

…………………………………………………………………………..

B7. a) Does she understand the concept of right and wrong?

Yes 1 No 2

b) Can she determine herself what is proper behaviour?

Yes usually 1 Yes sometimes 2 No 3 Don't know 9

B8. What does she consider important in her life? (tick all that apply)

		Yes
a)	school results	1
b)	relationship between herself and her teacher	1
c)	relationship between herself and her friends	1
d)	family relationships	1
e)	hobbies, interests	1
f)	friends	1

			Yes
B8.	g)	clothes	1
	h)	money	1
	i)	material possessions	1
	j)	holidays, trips	1

B9. What does she really like to do best? (tick all that apply)

		Yes
a)	sports	1
b)	playing a musical instrument	1
c)	singing	1
d)	dancing	1
e)	reading	1
f)	drawing	1
g)	making things	1
h)	other	1

(please tick and describe) ……………………………………………………

B10. Does she understand the following abstract terms?

		Yes	No
a)	later	1	2
b)	not at all	1	2
c)	far away	1	2
d)	infinite	1	2

B11. Does she understand the concept of death as an irreversible event with all its emotional consequences?

Yes, understands ☐ 1   Yes, more or less understands ☐ 2

Not really ☐ 3   Not at all ☐ 4

B12. Does she take an interest?

	**Yes, very interested**	**Yes, somewhat interested**	**No, not interested**	**Not sure**
a) in nationalism	☐ 1	☐ 2	☐ 3	☐ 4
b) in politics	☐ 1	☐ 2	☐ 3	☐ 4
c) in the meaning of life	☐ 1	☐ 2	☐ 3	☐ 4
d) in law and order	☐ 1	☐ 2	☐ 3	☐ 4
e) in religion	☐ 1	☐ 2	☐ 3	☐ 4

f) Does she attend a place of worship (church, mosque, etc)?

Yes often ☐ 1   Yes sometimes ☐ 2   No, not at all ☐ 3

g) Does she pray?

Yes often ☐ 1   Yes sometimes ☐ 2   No, not at all ☐ 3

Don't know ☐ 9

B13. a) What friends does she prefer?

boys ☐ 1

girls ☐ 2

she doesn't mind ☐ 3

she doesn't have friends ☐ 4

b) Does she have a favourite friend of the other sex?

Yes ☐ 1   No ☐ 2

B14. Is she at ease with children of her own age?

	Yes
yes, fully	1
prefers younger children	2
prefers older children	3

B15. How much is she influenced by her friends/mates?

very strongly	1
fairly strongly	2
sometimes	3
rarely	4
never	5

B16. Has she ever been offered:

	**Yes and I know about it**	**Probably**	**Possibly**	**I do not think so**
a) alcohol	1	2	3	4
b) cigarettes	1	2	3	4
c) drugs	1	2	3	4

B17. (i) Has she ever tried:

	**Yes and I know about it**	**Probably**	**Possibly**	**I do not think so**
a) alcohol	1	2	3	4
b) cigarettes	1	2	3	4
c) drugs	1	2	3	4

If yes to any of the above,

B17. (ii) At what age was she when she tried them? (put 99 if you don't know and 88 if she never tried them)

a)	alcohol	☐☐	years
b)	cigarettes	☐☐	years
c)	drugs	☐☐	years

The Children of the 90s study is in the forefront of new knowledge about children's health!

Over 100 scientific papers have been published or are in press in medical journals.

None of this would have been possible without your valuable help.

SECTION C: TALKING

Below are a number of statements about speech. Please indicate which are true for your study child.

		Certainly true	Somewhat true	Not true
C1.	People can understand almost everything she says	1	2	3
C2.	People have trouble in understanding much of what she says	1	2	3
C3.	She seldom makes any errors in producing speech sounds	1	2	3
C4.	She mispronounces one or two speech sounds but is not difficult to understand; e.g. may say “th” for “s” or “w” for “r”	1	2	3
C5.	Her production of speech sounds seems immature, like that of a younger child, e.g. she says “tat” for “cat”, or “chimbley” for “chimney”, or “bokkle” for “bottle”	1	2	3
C6.	She seems unable to produce several sounds; e.g. “k” or “s”, so that “cat” and “sat” are both pronounced as “tat”	1	2	3
C7.	She leaves off beginnings or ends of words, or omits entire syllables e.g. “bella” for “umbrella”	1	2	3
C8.	It is much harder to understand her when she is talking in sentences, rather than just producing single words	1	2	3
C9.	Her speech is extremely rapid	1	2	3
C10.	She seems to have difficulty in constructing the whole of what she wants to say: makes false starts, and repeats whole words and phrases; e.g. might say “can I-can-I-can-can I have an-have an ice cream”	1	2	3
C11.	Her words are clearly spoken and they flow one from another	1	2	3

		Certainly true	Somewhat true	Not true
C12.	Speech is mostly just 2 or 3 word phrases such as “me got ball” or “give dolly”	1	2	3
C13.	She can produce long and complicated sentences such as: “When we went to the park I had a go on the swings”; “I saw this man standing on the corner”	1	2	3
C14.	She tends to leave out words and grammatical endings, such as: “I find two dog”, “John go there yesterday”	1	2	3
C15.	Sometimes she makes mistakes with pronouns, e.g. saying “she” rather than “he” or vice versa	1	2	3
C16.	She talks to anyone and everyone	1	2	3
C17.	She talks too much	1	2	3
C18.	She keeps telling people things that they know already	1	2	3
C19.	She talks to herself	1	2	3
C20.	She talks repetitively about things that no-one is interested in	1	2	3
C21.	She asks questions although she knows the answers	1	2	3
C22.	It is sometimes hard to make sense of what she is saying because it seems illogical or disconnected	1	2	3
C23.	Conversation with her can be enjoyable and interesting	1	2	3
C24.	She can give an easy-to-follow account of a past event such as a birthday party or holiday	1	2	3

	Certainly true	Somewhat true	Not true
C25. She can talk clearly about what she plans to do in the future e.g. tomorrow or next week	1	2	3
C26. She would have difficulty in explaining to a younger child how to play a simple game such as “snap”	1	2	3
C27. She has difficulty in telling a story, or describing what she has done in a sequence of events	1	2	3
C28. She uses terms like “he” or “it” without making it clear what she is talking about	1	2	3
C29. She doesn’t seem to realise the need to explain what she is talking about to some-one who doesn’t share her experiences; e.g. might talk about “Johnny” without explaining who he is	1	2	3
C30. She pronounces words in an over-precise manner: accent may sound rather affected or “put-on”, as if child is mimicking a TV personality rather than talking like those around her	1	2	3
C31. She makes frequent use of expressions such as “by the way”, “actually”, “you know what?”, “as a matter of fact”, “well you know” or “of course”	1	2	3
C32. She will suddenly change the topic of conversation	1	2	3
C33. She often turns the conversation to a favourite theme, rather than following what the other person wants to talk about	1	2	3
C34. Conversation with her tends to go off in unexpected directions	1	2	3

		Certainly true	Somewhat true	Not true
C35.	She includes over-precise information in her talk, e.g. will give the exact time or date of an event. e.g. when asked “when did you go on holiday?” may say “13th July 1995” rather than “in the summer”	1	2	3
C36.	She has favourite phrases, sentences or longer sequences which she will use a great deal, sometimes in inappropriate situations	1	2	3
C37.	She sometimes seems to say things that she does not fully understand	1	2	3
C38.	She tends to repeat back what others have just said	1	2	3
C39.	Her ability to communicate clearly seems to vary a great deal from one situation to another	1	2	3
C40.	She takes in just one or two words in a sentence, and so often misinterprets what has been said	1	2	3
C41.	She can understand sarcasm e.g. will be amused rather than confused when some-one says “isn’t it a lovely day!” when it is pouring with rain	1	2	3
C42.	She tends to be over-literal, sometimes with (unintentionally) funny results. e.g. a child who was asked “Do you find it hard to get up in the morning” replied “No, you just put one leg out of the bed and then the other and stand up”	1	2	3
C43.	She gets into trouble, because she doesn’t always understand the rules for polite behaviour, and is regarded by others as rude or strange	1	2	3
C44.	She may say things which are tactless or socially inappropriate	1	2	3

	Certainly true	Somewhat true	Not true
C45. She treats everyone the same way, regardless of social status: e.g. she might talk to the head teacher the same way as to another child	1	2	3
C46. She ignores conversational overtures from others e.g. if asked "what are you making?" the child just continues working as if nothing had happened	1	2	3
C47. She hardly ever starts up a conversation; does not volunteer information about what has happened	1	2	3
C48. She doesn't seem to read facial expressions or tone of voice and may not realise when other people are upset or angry	1	2	3
C49. She is poor at using facial expressions or gestures to convey her feelings; she may look blank when angry, or smile when anxious	1	2	3
C50. She makes good use of gestures to get her meaning across	1	2	3
C51. She seldom or never looks at the person she is talking to: seems to actively avoid eye contact	1	2	3
C52. She tends to look away from the person she is talking to: seems inattentive or preoccupied	1	2	3
C53. She smiles appropriately when talking to people	1	2	3

Michelle and her sister

SECTION D: THE CHILD'S ACTIVITIES

D1. About how often does your child do the following:

How often does she:		Nearly every day	2-5 times a week	Once a week	1-3 times a month	Less than once per month	Not at all
a)	Go swimming	1	2	3	4	5	6
b)	Play a musical instrument (e.g. piano, recorder)	1	2	3	4	5	6
c)	Go to special groups (such as Scouts)	1	2	3	4	5	6
	Please tick and describe..						
d)	Go to Sunday School	1	2	3	4	5	6
e)	Go to special classes or clubs for some activity (e.g. dancing, judo, sports)	1	2	3	4	5	6
	Please tick and describe..						
f)	Go to special classes because of learning difficulty	1	2	3	4	5	6
	Please tick and describe..						
g)	Classes for foreign languages	1	2	3	4	5	6
	Please tick and describe..						
h)	Singing group	1	2	3	4	5	6
	Please tick and describe..						
i)	Other type of classes or group	1	2	3	4	5	6
	Please tick and describe..						

D1. **How often does she:**		**Nearly every day**	**2-5 times a week**	**Once a week**	**1-3 times a month**	**Less than once per month**	**Not at all**
j)	See her grandparents	1	2	3	4	5	6
k)	Play computer games	1	2	3	4	5	6
l)	Help in the house	1	2	3	4	5	6

D2. How often does her mother or other adult female do these activities with her?

	**Adult female:**	**Nearly every day**	**2-5 times a week**	**Once a week**	**Less than once a week**	**Never** ↓
a)	bath (or showers) her	1	2	3	4	5
b)	makes things with her	1	2	3	4	5
c)	sings with her	1	2	3	4	5
d)	reads to/with her	1	2	3	4	4
e)	plays with toys	1	2	3	4	5
f)	cuddles her	1	2	3	4	5
g)	active play (e.g. ball games, wrestling, hide and seek)	1	2	3	4	5
h)	takes her to the park or playground	1	2	3	4	5
i)	puts her to bed	1	2	3	4	5
j)	takes her swimming, fishing or other activity	1	2	3	4	5
k)	draws or paints with her	1	2	3	4	5
l)	prepares food with her	1	2	3	4	5

D2.

	Adult female:	Nearly every day	2-5 times a week	Once a week	Less than once a week	Never
m)	takes her to classes	1	2	3	4	5
n)	takes her shopping	1	2	3	4	5
o)	takes her to watch sports/ football	1	2	3	4	5
p)	does homework with her	1	2	3	4	5
q)	has conversations with her	1	2	3	4	5
r)	helps her prepare things for school	1	2	3	4	5
s)	other (please tick & describe)	1	2	3	4	5

....

t) Who are the women involved in any of these activities with the study child? (tick all that apply)

i)	Her mother	1
ii)	Her stepmother/father's partner	1
iii)	Her grandmother	1
iv)	Her grown-up sister	1
v)	Another relative	1
vi)	A family friend	1
vii)	A lodger	1
viii)	A baby sitter/nanny	1
ix)	Other (please tick and describe)	1

....

D3. How often does a male adult (e.g. her father/mother's husband or partner) do these activities with your child?

**Adult male:**	**Nearly every day**	**2-5 times a week**	**Once a week**	**Less than once a week**	**Never** ↓
a) bath (or showers) her	1	2	3	4	5
b) makes things with her	1	2	3	4	5
c) sings with her	1	2	3	4	5
d) reads to/with her	1	2	3	4	5
e) plays with toys	1	2	3	4	5
f) cuddles her	1	2	3	4	5
g) active play (e.g. ball games, wrestling, hide and seek)	1	2	3	4	5
h) takes her to the park or playground	1	2	3	4	5
i) puts her to bed	1	2	3	4	5
j) takes her swimming, fishing or similar activity	1	2	3	4	5
k) draws or paints with her	1	2	3	4	5
l) prepares food with her	1	2	3	4	5
m) takes her to classes	1	2	3	4	5
n) takes her shopping	1	2	3	4	5
o) take her to watch sports/ football	1	2	3	4	5
p) does homework with her	1	2	3	4	5
q) has conversations with her	1	2	3	4	5
r) helps her prepare things for school	1	2	3	4	5

D3. Adult male:	Nearly every day	2 - 5 times a week	Once a week	Less than once a week	Never ↓
s) other (please tick and describe)	1	2	3	4	5

....

t) Who are the men involved in any of these activities with the study child? (tick all that apply)

i)	Her father	1
ii)	Her stepfather/mother's partner	1
iii)	Her grandfather	1
iv)	Her grown-up brother	1
v)	Another relative	1
vi)	A family friend	1
vii)	A lodger	1
viii)	A baby sitter/nanny	1
ix)	Other (please tick and describe)	1

....

D4. Help in the house:

a) Does your daughter help in the home (cleaning, washing dishes etc.)?

Yes, but only if made to	1
Yes, sometimes offers to and sometimes is made to	2
Yes, always offers to	3
No, refuses to help	4
No, is not allowed to help	5

D4. b) If **not allowed**, why is this?……………………………………………………

………………………………………………………………………………………..

D5. Does she have a space in which she can do things on her own?

Yes, her own bedroom ☐ 1

A corner of a room ☐ 2

Her own table ☐ 3

No, there is no room for this ☐ 4

Something else
(please tick and describe) ☐ 5

……………………………………………………………………………..

D6. a) Does she have brothers and/or sisters living at home (include step and half-brothers and sisters)?

Yes ☐ 1 No ☐ 2 ⟶ **If no, go to D7 on page 33**

If yes,

b) How many?

i) older brothers ☐☐ older sisters ☐☐

ii) younger brothers ☐☐ younger sisters ☐☐

iii) twin brother ☐ twin sister ☐

Please give the age of: (Put 99 if no such person. Put 00 if less than 1 year old)

iv) oldest brother ☐☐ years old

v) oldest sister ☐☐ years old

vi) youngest brother ☐☐ years old

vii) youngest sister ☐☐ years old

D6. c) How often does she do the following with them?

**With her brothers and sisters**	**Nearly every day**	**2-5 times a week**	**Once a week**	**Less than once a week**	**Never**
(i) Play with toys	1	2	3	4	5
(ii) Read together	1	2	3	4	5
(iii) Sing together	1	2	3	4	5
(iv) Make things, draw or paint	1	2	3	4	5
(v) Go out together	1	2	3	4	5
(vi) Talk together	1	2	3	4	5
(vii) Eat together	1	2	3	4	5
(viii) Argue with one another	1	2	3	4	5
(ix) Do sports together	1	2	3	4	5

D7. a) Does she wear clothes that have been handed down free from others? (tick all that apply)

i)	yes, from older brothers & sisters	1
ii)	yes, from other relatives	1
iii)	yes, from friends	1

b) Does she ever have clothes bought second hand for her?

Yes 1 No 2

SECTION E: MOODS AND FEELINGS

These questions are about how your child may have been feeling or acting recently.
For each question, please say how much she has felt or acted this way in the past two weeks.

		True	Sometimes true	Not true
E1.	She felt miserable or unhappy	1	2	3
E2.	She didn't enjoy anything at all	1	2	3
E3.	She felt so tired that she just sat around and did nothing	1	2	3
E4.	She was very restless	1	2	3
E5.	She felt she was no good any more	1	2	3
E6.	She cried a lot	1	2	3
E7.	She found it hard to think properly or concentrate	1	2	3
E8.	She hated herself	1	2	3
E9.	She felt she was a bad person	1	2	3
E10.	She felt lonely	1	2	3
E11.	She thought nobody really loved her	1	2	3
E12.	She thought she could never be as good as other kids	1	2	3
E13.	She felt she did everything wrong	1	2	3

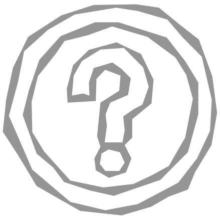

DID YOU KNOW? ……

There are Children of the 90s families in 40 different countries!

SECTION F: STRENGTHS AND DIFFICULTIES

Please think how your child has been in the past 6 months

	**In the last six months:**	**Not true**	**Somewhat true**	**Certainly true**	**Don't know**
F1.	She has been considerate of other people's feelings	1	2	3	4
F2.	She has been restless, overactive, cannot stay still for long	1	2	3	4
F3.	She has often complained of headaches, stomach aches or sickness	1	2	3	4
F4.	She has shared readily with other children (treats, toys, pencils etc.)	1	2	3	4
F5.	She has often had temper trantrums or hot tempers	1	2	3	4
F6.	She is rather solitary, tends to play alone	1	2	3	4
F7.	She is generally obedient, usually does what adults request	1	2	3	4
F8.	She has many worries, often seems worried	1	2	3	4
F9.	She is helpful if someone is hurt, upset or feeling ill	1	2	3	4
F10.	She is constantly fidgeting or squirming	1	2	3	4
F11.	She has at least one good friend	1	2	3	4
F12.	She often fights with other children or bullies them	1	2	3	4
F13.	She is often unhappy, down-hearted or tearful	1	2	3	4
F14.	She is generally liked by other children	1	2	3	4

	In the last six months:	Not true	Somewhat true	Certainly true	Don't know
F15.	She is easily distracted, her concentration wanders	1	2	3	4
F16.	She is nervous or clingy in new situations, easily loses confidence	1	2	3	4
F17.	She is kind to younger children	1	2	3	4
F18.	She often lies or cheats	1	2	3	4
F19.	She is picked on or bullied by other children	1	2	3	4
F20.	She often volunteers to help others (parents, teachers, other children)	1	2	3	4
F21.	She thinks things out before acting	1	2	3	4
F22.	She steals from home, school or elsewhere	1	2	3	4
F23.	She gets on better with adults than with other children	1	2	3	4
F24.	She has many fears, is easily scared	1	2	3	4
F25.	She sees tasks through to the end, has good attention span	1	2	3	4

Sam Lauren

SECTION G : SLEEPING

G1. a) Does your child have a regular sleeping routine?

Yes ☐ 1 No ☐ 2

b) How often during the night does she usually wake? ☐☐ times

G2. a) Where does she usually sleep?

	(i) When she goes to bed at night	(ii) When she wakes in the morning
in her own room on her own	☐ 1	☐ 1
in a room with other children	☐ 2	☐ 2
in your bedroom	☐ 3	☐ 3
in a room with other adults	☐ 4	☐ 4
other place (please tick & describe)	☐ 5	☐ 5

....

b) Does she sleep on her own most nights or does she share a bed?

	(i) When she goes to bed at night	(ii) When she wakes in the morning
in her own bed	☐ 1	☐ 1
in a bed with other children	☐ 2	☐ 2
in your bed with you	☐ 3	☐ 3
in a bed with other adult	☐ 4	☐ 4
other place (please tick & describe)	☐ 5	☐ 5

....

G2. c) How often does she sleep:

	Always	Usually	Sometimes	Hardly ever
i) on her back	1	2	3	4
ii) on her side	1	2	3	4
iii) on her front	1	2	3	4

d) Does your child seem to grind her teeth :

	Yes, often	Yes, sometimes	No
i) when she's asleep?	1	2	3
ii) at other times?	1	2	3

e) In the room where the child sleeps most of the night:

	In Winter			In Summer		
	**Yes always**	**Yes some-times**	**No not at all**	**Yes always**	**Yes some-times**	**No not at all**
i) is the heating on all night?	1	2	3	1	2	3
ii) is the heating on part of the night?	1	2	3	1	2	3
iii) is there a window open at night?	1	2	3	1	2	3
iv) does she sleep with a duvet?	1	2	3	1	2	3
v) does she have an electric blanket?	1	2	3	1	2	3
vi) does she sleep with a pillow?	1	2	3	1	2	3

G3. **In the past year** has your child regularly:

		Yes, but did not worry me	Yes, worried me a bit	Yes, worried me greatly	No, did not happen	Don't know
a)	refused to go to bed	1	2	3	4	9
b)	woken very early	1	2	3	4	9
c)	had difficulty going to sleep	1	2	3	4	9
d)	had nightmares	1	2	3	4	9
e)	continued to get up after being put to bed	1	2	3	4	9
f)	refused to go to bed	1	2	3	4	9
g)	got up after only a few hours sleep	1	2	3	4	9

Samuel Paige

SECTION H: BITS AND PIECES

Please list the dates on which your child was weighed or measured since she was 7 years old and add her height and weight. If you don't know, please write DK and go to H2 below.

		Date	Weight	Height
H1.	a)	...../....../….....	....	....
	b)	..../....../….....	....	....
	c)	..../....../….....	....	....
	d)	..../....../….....	....	....

H2. What size shoes does she take? ....

H3. How often nowadays does she wear the following footwear:

		(i) Out of doors			(ii) Indoors		
		Usually	Sometimes	Never	Usually	Sometimes	Never
a)	sandals	1	2	3	1	2	3
b)	trainers/ plimsolls	1	2	3	1	2	3
c)	slippers	1	2	3	1	2	3
d)	shoes	1	2	3	1	2	3
e)	other (please tick and describe)	1	2	3	1	2	3

....

H4. How long do you usually let her grow her hair before cutting it?

less than 1 inch 1 | more than 1 inch but not shoulder length 2 | shoulder length 3

longer than shoulder 4 | never has it cut 5 | other, please describe 6

....

H5. How many of the following immunisations has she had since the age of 7?
(If you don't know put 9)

	**Number**
a) BCG (for tuberculosis)	☐
b) DTP or Triple (includes whooping cough)	☐
c) DT (without whooping cough)	☐
d) Polio	☐
e) MMR (measles, mumps and rubella)	☐
f) Hib (Haemophylus influenzae B - for meningitis)	☐
g) Other (please tick and describe)	☐

…………………………………………………………………………

h) Did she have a temperature or was she unwell after any of these immunisations?

Yes ☐ 1 No ☐ 2 → **If no, go to I1 on page 42**

If yes, please describe:

i) which immunisation: …………………………………………………………..

ii) how old was she? ☐ years

iii) how long after the immunisation did this start?

under 3 hours ☐ 1	3-24 hours ☐ 2	1-2 days ☐ 3
3-6 days ☐ 4	1 week or more ☐ 5	don't know ☐ 9

iv) how was she affected? ………………………………………………………………

……………………………………………………………………………………………..

SECTION I: USING THE TOILET

I1. When she needs to go to the toilet to urinate (pass water):

a) How often does she show signs (e.g. fidgets) that she needs to go?

never ☐1 sometimes ☐2 often ☐3

b) When she needs to, how often does she go to the toilet without you having to remind her?

never ☐1 sometimes ☐2 often ☐3 always ☐4

c) Does she have to dash to the toilet quickly when she realises she needs to go?

yes, has to go straight away ☐1

can hold for a short time (less than 5 minutes) ☐2

can hold for longer than 5 minutes ☐3

d) How often does she usually go to the toilet to pass water during the day?

less than 5 times a day ☐1 5-9 times a day ☐2

10 or more times a day ☐3 don't know ☐9

e) How often does she usually get up to go to the toilet at night?

not at all ☐1 once ☐2 twice ☐3

3 or more times ☐4 don't know ☐9

I1. How often usually does your child:

	Never	Occasional accident but less than once a week	About once a week	2-5 times a week	Nearly every day	More than once a day
f) dirty her pants during the day	1	2	3	4	5	6
g) dirty herself at night	1	2	3	4	5	6
h) wet herself during the day	1	2	3	4	5	6
i) wet the bed at night	1	2	3	4	5	6

j) If she wets at night, how often does she:

	never	sometimes	often	always
(i) wake up after wetting	1	2	3	4
(ii) seem to wet soon after going to sleep	1	2	3	4
(iii) seem upset when the bed is wet	1	2	3	4

I2. Which of the following methods have you tried in the past or are using now to try and help your child stop wetting <u>during the day</u>? (Please note: this is <u>not</u> a list of ways which are necessarily effective in helping children to stop wetting during the day).

**Tick all that apply:**	**(i) Have used in the past**	**(ii) Using now**
a) taking/sending her to the toilet regularly	1	1
b) encouraging child to 'hold on' before using the toilet	1	1
c) drinking more fluids	1	1
d) drinking less fluids	1	1
e) medication	1	1

Tick all that apply:	(i) Have used in the past	(ii) Using now
I2. f) praising for dry pants	☐ 1	☐ 1
g) showing your displeasure	☐ 1	☐ 1
h) advice from a health worker (e.g. health visitor, school nurse, GP, hospital doctor, social worker, psychologist)	☐ 1	☐ 1

Please tick and describe ……………………………………………………………

I3. Which of the following methods have you tried in the past or are you using now to try and help your child stop <u>wetting the bed</u>? (Please note: this is <u>not</u> a list of ways which are necessarily effective in helping children to stop wetting the bed).

Tick all that apply:	(i) Have used in the past	(ii) Using now
a) lifting (taking your child out of bed to the toilet)	☐ 1	☐ 1
b) restricting drinks before bed	☐ 1	☐ 1
c) getting her to toilet regularly in the day	☐ 1	☐ 1
d) rewarding for being dry	☐ 1	☐ 1
e) medication	☐ 1	☐ 1
f) homeopathy	☐ 1	☐ 1
g) hypnosis	☐ 1	☐ 1
h) an alarm that wakes the child when she wets	☐ 1	☐ 1
i) showing your displeasure	☐ 1	☐ 1
j) using night-time protection pants or nappies	☐ 1	☐ 1
k) advice from a health worker (e.g. health visitor, school nurse, GP, hospital doctor, social worker, psychologist)	☐ 1	☐ 1

Please tick and describe ……………………………………………………………

David

Charlotte

Leo

SECTION J: EATING

J1. In the past year have you had difficulties getting her to eat what you wanted her to?

Yes, great difficulty	1	→ **If yes, please describe the problems:**
Yes, some difficulty	2	……………………………………………
Yes, occasional difficulty	3	……………………………………………
No, no difficulty	4	

J2. In the past year has she at any time:

		**Yes, worried me greatly**	**Yes, worried me a bit**	**Yes, but did not worry me**	**No did not happen**
a)	deliberately not eaten enough food	1	2	3	4
b)	refused to eat the food you think she should eat	1	2	3	4
c)	been choosy with food	1	2	3	4
d)	over-eaten	1	2	3	4
e)	been difficult to get into an eating routine	1	2	3	4

J3. **On school days** how often does your study child usually eat at each of the following times of day?

	**Never**	**Once in 2 weeks**	**Once a week**	**2-4 times a week**	**5 times a week**	**Don't know**
a) Before school	1	2	3	4	5	9
b) Mid-morning	1	2	3	4	5	9
c) Midday	1	2	3	4	5	9

	Never	Once in 2 weeks	Once a week	2-4 times a week	5 times a week	Don't know
J3. d) Mid-afternoon before 4.30 p.m.	1	2	3	4	5	9
e) Late afternoon, between 4.30 & 6.00 p.m.	1	2	3	4	5	9
f) Early evening between 6.00 & 7.30 p.m.	1	2	3	4	5	9
g) Mid-evening between 7.30 & 9.00 p.m.	1	2	3	4	5	9
h) Late evening after 9.00 p.m.	1	2	3	4	5	9

J4. How many times a week on school days does your study child have the following foods or drinks before school?

**Before school:**	**Never**	**Once in 2 weeks**	**Once a week**	**2-4 times a week**	**5 times a week**	**Don't know**
a) Nothing to eat or drink	1	2	3	4	5	9
b) Has a drink but nothing to eat	1	2	3	4	5	9
c) Has cereal without milk	1	2	3	4	5	9
d) Has cereal with milk	1	2	3	4	5	9
e) Has bread or toast	1	2	3	4	5	9
f) Has bacon, egg, sausage, or cheese	1	2	3	4	5	9
g) Has crisps, corn snack or other savoury snack	1	2	3	4	5	9
h) Has sweet biscuits, sweets or chocolates	1	2	3	4	5	9
i) Has fruit, yoghurt or fromage frais	1	2	3	4	5	9

	Never	Once in 2 weeks	Once a week	2-4 times a week	5 times a week	Don't know
**Before school:**						
J4. j) Has a milk drink	1	2	3	4	5	9
k) Has other food (Please tick & describe)	1	2	3	4	5	9

……………………………………………………………………………………………..

J5. How many times a week on school days does your study child have as her midday meal?

	Never	Once in 2 weeks	Once a week	2-4 times a week	5 times a week	Don't know
a) Cooked meal at school	1	2	3	4	5	9
b) Packed lunch provided by school	1	2	3	4	5	9
c) Packed lunch provided from home	1	2	3	4	5	9
d) Comes home for a snack lunch	1	2	3	4	5	9
e) Comes home for a main meal at midday	1	2	3	4	5	9

J6. How often do you ask your study child about the food she has eaten at school?

Never	Occasionally	Quite often	Most days	Does not eat at school
1	2	3	4	5

J7. How many times a week on **school days** does your study child have for her **tea/evening meal**

**Tea/evening meal**	**Never**	**Once in 2 weeks**	**Once a week**	**2-4 times a week**	**5 times a week**	**Don't know**
a) Cooked meal with no vegetables or salad	1	2	3	4	5	9
b) Cooked meal with fresh vegetables or salad	1	2	3	4	5	9
c) Cooked meal with frozen vegetables	1	2	3	4	5	9
d) Cooked meal with tinned vegetables	1	2	3	4	5	9
e) Sandwich or snack meal e.g. Baked beans on toast, pot noodles	1	2	3	4	5	9
f) Sitting up at a table	1	2	3	4	5	9
g) Eaten from a tray/plate on her lap	1	2	3	4	5	9
h) Eaten using a knife and fork	1	2	3	4	5	9
i) Eaten using a spoon and/or fingers	1	2	3	4	5	9
j) Adult(s) eating with her	1	2	3	4	5	9
k) Other children eating with her but not adults	1	2	3	4	5	9
l) Eating on her own	1	2	3	4	5	9

J8. a) Does your child usually drink milk on a school day?

Yes ☐1 No ☐2 → **If no, go to J9 below**

If yes,

	**Yes**	**No**
b) Does she have milk at school?	☐1	☐2
c) Does she have milk at home?	☐1	☐2

d) If milk is drunk at home about how much does she have in total in drinks and on cereal at home?

Up to ½ pint	½-1 pint	1-1½ pints	1½-2 pints	more than 2 pints
☐1	☐2	☐3	☐4	☐5

J9. How many normal size pots (100-150g) of yoghurt or fromage frais does your child eat each week in total (2 tiny pots equals one normal pot)?

None	one or less	2	3-4	5-6	one per day	more than one per day
☐1	☐2	☐3	☐4	☐5	☐6	☐7

J10. Has your child had any of the following supplements in the last 12 months?

		**Never**	**Yes, takes them most days`**	**Yes takes them occasionally**	**If yes, please give full name**
a)	Multivitamins	☐1	☐2	☐3	………………..
b)	Multiminerals	☐1	☐2	☐3	………………..
c)	Calcium	☐1	☐2	☐3	………………..
d)	Iron	☐1	☐2	☐3	………………..
e)	Vitamin D	☐1	☐2	☐3	………………..
f)	Vitamin C	☐1	☐2	☐3	………………..
g)	Cod liver oil	☐1	☐2	☐3	………………..
h)	Other		☐2	☐3	………………..

J11. Is your child at present a vegetarian?

Yes ☐1 No ☐2

J12. Is your child at present a vegan (i.e. does not eat meat, poultry, fish, eggs, butter, milk or cheese)?

Yes ☐1 No ☐2

J13. Is your child at present on any other kind of special diet?

Yes ☐1 No ☐2 → **If no, go to J14 below**

If yes, please describe below.

....

....

J14. Does your child have definite likes and dislikes as far as food is concerned?

no, will eat almost anything ☐1

yes, quite choosy ☐2

yes, very choosy ☐3

J15. Does she eat coal, soil, dirt or other non-food substances?

yes, every day ☐1

yes, at least once a week ☐2

yes, less than once a week ☐3

→ **If yes, please describe what she eats:** ....

no, not at all ☐4

SECTION K:

K1. This questionnaire was completed by (please tick all that apply):

	Yes		Yes
a) child's biological mother	1	d) study child	1
b) child's mother-figure	1	e) someone else (please describe) …………	1
c) child's biological father	1		

K2. Please give the date on which you completed this questionnaire:

day ☐☐ month ☐☐ year 2 0 0 ☐

K3. Please give your date of birth:

day ☐☐ month ☐☐ year 19 ☐☐

K4. Please give your study child's date of birth:

day ☐☐ month ☐☐ year 199 ☐

THANK YOU VERY MUCH FOR YOUR HELP

Space for any additional comments you would like to make

NB Please remember we cannot reply to any comment unless you sign it.

When completed, please return the questionnaire to:

Professor Jean Golding
Children of the Nineties - ALSPAC
Institute of Child Health
24 Tyndall Avenue
Bristol BS8 1BR
Tel: Bristol 9285007

For office use only
Coder ☐☐ *Int* ☐☐

© University of Bristol
