# Supplementary material for: Breastfeeding, prenatal depression and children’s IQ and behaviour: a test of a moderation model
Source: BMC Pregnancy Childbirth. 2021 Jan 18;21:62. doi: 10.1186/s12884-020-03520-8 (PMC7814604; doi:10.1186/s12884-020-03520-8)
Supplement: Supplementary file 5 — Additional file 5:. ques-cb10-development-and-health-of-my-son. Questionnaire data collected when the child was 57 months old including the SDQ sub-scales used in this study. [file 12884_2020_3520_MOESM5_ESM.pdf]

© University of Bristol

DEVELOPMENT AND HEALTH OF MY DAUGHTER

This questionnaire asks some more questions about your study child. We are interested to know about her health and behaviour and how she gets on with other children.

This questionnaire is like the other questionnaires you have received. To answer simply tick the box which best describes your child or your child's situation. Please answer all questions that you can. If you cannot answer any question or if they do not apply to you please put a line through them. There are no right or wrong answers. Please just describe what happens in your situation. Your may make additional comments at the end. All answers are confidential.

Many of the questions ask you to describe what has happened since your child was 3 - by this we mean since her 3rd birthday.

THANK YOU FOR YOUR HELP

08/12/95

SECTION A: YOUR CHILD'S HEALTH

A1. How would you assess the health of your child now?

	(i) in the past month	(ii) in the past year
very healthy, no problems	1	1
healthy, but a few minor problems	2	2
sometimes quite ill	3	3
almost always unwell	4	4

A2. Has she had fluoride supplements (tablets or drops) in the past 2 years?

Yes 1 No 2 → **If no or not known go to A3a below** ← Not known 3

If yes, (i) for how long did she have them?

less than 1 month 1 1-2 months 2 3-5 months 3 6-11 months 4

more than 12 months 5 don't know 9

ii) How old was she when she last had fluoride supplements? (put 6 if she still has them) ☐ years old

A3. a) Since your child was 3 years old, has the doctor been called to your home because she was unwell?

Yes 1 No 2 → **If no, go to A4 on page 3**

If yes,

b) how many times?

once	1	twice	2	3-4 times	3	5 or more times	4

A4. Has she had any of the following since she was 3 years old?

**Since 3 years old**		**Yes and saw a doctor**	**Yes but did not see doctor**	**No did not have**
a)	diarrhoea	1	2	3
b)	blood in the stools	1	2	3
c)	vomiting	1	2	3
d)	cough	1	2	3
e)	high temperature	1	2	3
f)	snuffles/cold	1	2	3
g)	ear ache	1	2	3
h)	ear discharge (pus not wax)	1	2	3
i)	convulsions/fits	1	2	3
j)	stomach ache(s)	1	2	3
k)	rash	1	2	3
l)	wheezing	1	2	3
m)	breathlessness	1	2	3
n)	episodes of stopping breathing	1	2	3
o)	an accident	1	2	3
p)	urinary infection	1	2	3

**Since 3 years old**		**Yes and saw a doctor**	**Yes but did not see doctor**	**No did not have**
q)	headache(s)	1	2	3
r)	constipation	1	2	3
s)	worm infections	1	2	3
t)	lice or scabies	1	2	3
u)	other (please tick and describe)	1	2	3

....

A5. a) Has your child been admitted to hospital since she was 3 years old?

Yes [1] No [2] → **If <u>no</u>, go to A6 on page 5**

If <u>yes</u>,

b) how many times? [ ][ ]

. c) please describe for each admission:

	**Age of child (years)**	**Reason for admission**	**No. of nights child stayed in hospital**
1.	[ ]	....	[ ][ ]
2.	[ ]	....	[ ][ ]
3.	[ ]	....	[ ][ ]

A5. d) How often did you see her while she was in hospital?

	1st admission	2nd admission	3rd admission
Not at all	1	1	1
Quite often	2	2	2
Every day	3	3	3
Stayed in the hospital with her	4	4	4

A6. Has she **ever** had any of the following operations?

		Yes	No
a)	hernia repair	1	2
b)	tonsils out	1	2
c)	adenoids out	1	2
d)	appendicectomy (appendix out)	1	2
e)	tubes (grommets) put in her ears	1	2
f)	squint repair (to put eyes straight)	1	2
g)	teeth pulled out	1	2
h)	other operations (please tick and describe)	1	2

....

....

A7. a) Since she was 3 years old has she had any periods when there was wheezing with whistling on her chest when she breathed?

Yes 1 No 2 → **If no, go to A7g on page 7**

If yes,

A7. b) How many separate times has this happened since she was 3 years old?

once [1] twice [2] 3-4 times [3] 5 or more times [4] don't know [9]

c) How many days altogether would you say she had wheezed since she was 3 years old?

1 day [1] 2-3 days [2] 4-9 days [3] 10-19 days [4] 20 or more days [5] don't know [9]

d) Was she breathless during any of these times?

Yes for all [1] Yes for some [2] No not at all [3]

e) Did she have a fever during any of these times?

Yes for all [1] Yes for some [2] No not at all [3]

f) What do you think brings the wheezing attacks on?

		Yes	No
i)	chest infection or bronchitis	1	2
ii)	being in a smoky room	1	2
iii)	cold weather	1	2
iv)	I don't know	1	2
v)	other (please tick and describe)	1	2

....

....

A7. g) Have any of your other children had spells of wheezing with whistling on the chest?

Yes ☐ 1   No ☐ 2   have no other children ☐ 7

A8. a) Has your child had any itchy, dry skin rash in the joints and creases of her body (e.g. behind the knees, elbows, under the arms) since she was 3 years old?

Yes ☐ 1   No ☐ 2   → **If no, go to A9a below**

If yes,

b) how bad was this?

very bad ☐ 1   quite bad ☐ 2   mild ☐ 3   no problem ☐ 4

c) does she have this sort of rash now?

Yes ☐ 1   No ☐ 2

		**Yes**	**No**
d)	did the rash ever become sore and oozy?	☐ 1	☐ 2
e)	was it made worse by irritants such as bubble bath, soap, wool or nylon clothing?	☐ 1	☐ 2

A9. a) Has she had an itchy, dry, rash on her hands since she was 3 years old?

Yes ☐ 1   No ☐ 2

b) Has she had an itchy, dry rash on her feet since she was 3 years old?

Yes ☐ 1   No ☐ 2   → **If no, go to A9c on page 8**

If yes, please describe which parts of her feet ....

A9. c) Does her skin get itchy when she gets sweaty? (e.g. in a hot room or when she has been playing)

Yes ☐1 No ☐2

A10. Has she ever had a skin reaction (e.g. redness or itching) which you thought was due to some food that she had eaten?

Yes ☐1 No ☐2 → **If <u>no</u>, go to A11a below**

If <u>yes</u>,

i) please describe the food(s) ....

ii) how long after the food was eaten did the reaction appear?...............

iii) where was the reaction? mouth ☐1

other part ☐2 → please say where ....

....

A11. a) Has she had vomiting spells since she was 3 years old?

Yes ☐1 No ☐2 → **If <u>no</u>, go to A12a on page 9**

If <u>yes</u>,

b) How many times?

once ☐1 twice ☐2 3-9 times ☐3 10 or more times ☐4

A11. c) With the vomiting attacks, did she also have:

	**Always**	**Frequently**	**Sometimes**	**Rarely**	**Never**
i) diarrhoea	1	2	3	4	5
ii) chestiness (wheezing or coughing or grunting)	1	2	3	4	5

A12. a) Since she was 3 years old has she had diarrhoea or gastro-enteritis?

Yes 1 No 2 → **If no go to A13a on page 10**

If yes,

b) how many times? ☐☐

c) how many days did the worst attack last? ☐☐

d) Did you:

	**Yes**	**No**
i) call the doctor to come to your home?	1	2
ii) go to your doctor?	1	2
iii) treat it yourself?	1	2
iv) do something else? (please tick and describe)	1	2

....

e) Did she continue to eat as usual?

Yes 1 → **If yes, go to A12f on page 10**

No 2

If <u>no</u>, i) how long was normal feeding disturbed?

less than 1 day [1] 1 day [2] 2 days [3]

3-4 days [4] 5 or more days [5]

A12. f) Was she given an oral rehydration solution?

Yes [1] No [2] Don't know [9]

↓

If <u>no</u> or <u>don't know,</u> go to A12g below

If yes, i) give type if known: ....

ii) how long was the solution given?

less than 1 day [1] 1 day [2] 2 days [3]

3-4 days [4] 5 or more days [5]

A12. g) What other treatment was given?

....

....

A13. a) Since she was 3 years old has your child ever had a time when she has coughed off and on for at least 2 days?

Yes [1] No [2] → **If <u>no</u>, go to A14a on page 11**

If <u>yes</u>,

b) how many times has this happened since she was 3 years old?

once [1] twice [2] 3-9 times [3] 10 or more times [4]

A13. c) Did she have a fever at any of these times?

Yes for all [1] Yes for some [2] No, not at all [3]

d) Did she have a runny nose during any of these spells?

Yes for all [1] Yes for some [2] No, not at all [3]

A14. The following questions are about your child's ears or hearing.

a) Nowadays, does your child listen to people or to things that happen nearby:

Yes always	1	Yes often	2
Sometimes	3	Usually not	4
Never	5	Child unable to hear at all	7

b) Does she turn her head towards sounds?

yes usually	1
yes sometimes	2
only to very loud sounds	3
never turns towards sounds	4
don't know	9

c) <u>During or after a cold,</u> is her hearing worse than usual?

yes, much worse	1	yes, a little worse	2
no, about the same	3	don't know	9
has never had a cold	7		

A14. d) During recent colds, is the dripping (discharge) from her nose:

		**Yes usually**	**Yes sometimes**	**No never**	**Don't know**	**Hasn't had a cold**
i)	clear	1	2	3	9	7 → **Go to A14e below**
ii)	slightly white in colour	1	2	3	9	
iii)	thick heavy yellow and/or green in colour (catarrh)	1	2	3	9	
iv)	very little discharge occurs at all	1	2	3	9	

e) Has pus or sticky mucus (not ear wax) leaked out of her ear since she was 3 years old?

never	1
once	2
more than once	3
don't know	9

f) Does she breathe through her mouth rather than through her nose?

	**(i) when asleep**	**(ii) when awake**
all the time	1	1
much of the time	2	2
sometimes	3	3
rarely	4	4
never	5	5
don't know	9	9

A14. g) Does she snore for more than a few minutes at a time?

most nights	1
quite often	2
sometimes	3
only rarely	4
never	5
don't know	9

h) When she is asleep, does she seem to stop breathing or hold her breath for several seconds at a time?

yes, often	1
yes, sometimes	2
no	3
don't know	9

A15. a) Have there been times since she was 3 years old when she has had a pain in her stomach?

Yes 1 No 2 → **If <u>no</u>, go to A16a on page 14**

If <u>yes</u>,

b) How many separate times has this happened since she was 3 years old?

once 1 twice 2 3-4 times 3 5 or more times 4 don't know 9

c) Did she have vomiting or diarrhoea at the same time as the pain?

yes every time 1 yes, for some of the the times 2 no, not at all 3

A15. d) What do you think are the causes of her stomach pains (tick all that apply)?

i) something she ate [ ] 1

ii) an infection [ ] 1

iii) constipation [ ] 1

iv) other (please describe) [ ] 1

....

v) don't know [ ] 1

A16. a) Does she often have aches and pains in her arms or legs?

yes arm(s) [ ] 1 yes leg(s) [ ] 2 yes both [ ] 3 no, not often [ ] 4

↓

If no, go to A17a on page 15

b) **If yes,**

i) does this happen especially when she is tired? Yes [ ] 1 No [ ] 2

ii) what do you think is the cause ?

....

iii) do you find any particular treatment helps ?

Yes [ ] 1 No [ ] 2

If yes, please describe...

A17. a) Since she was 3 years old has she had any form of convulsion, fit, seizure or other turn in which consciousness was lost or any part of the body made an abnormal movement?

Yes [ ]1 No [ ]2 Not known [ ]9

If no, or <u>not known</u>, go to A18 on page 17

If <u>yes</u>,

b) Please describe the first attack since her 3rd birthday:

....

....

c) Did she have a high temperature at the time?

Yes [ ]1 No [ ]2 Not known [ ]9

d) How old was she at the time?

3 years [ ]1 4 years [ ]2 5 years old [ ]3

e) How many attacks has she had since her 3rd birthday?

one [ ]1 two [ ]2 3-4 [ ]3 5 or more [ ]4

A17. f) By whom was she seen for these attack(s)? (tick all that apply)

		Yes
i)	general practitioner (GP) at home	1
ii)	GP at surgery	1
iii)	hospital outpatients	1
iv)	admitted to hospital	1

g) What investigations, if any, have been carried out?

....

....

h) Did later attacks differ from the first one ?

Yes 1 No 2 → **If no, go to (j) below**

If yes, please describe ....

....

j) What were these thought to be due to? (Tick all that apply)

i)	febrile convulsions	1
ii)	fainting and blackouts	1
iii)	epilepsy	1
iv)	breath holding	1
v)	reaction to immunisation	1
vi)	other (please specify)	1 ....
vii)	don't know	1

A18. Since she was 3 years old, has she had the following infections?

		Yes	No
a)	measles	1	2
b)	chicken pox	1	2
c)	mumps	1	2
d)	meningitis	1	2
e)	cold sores	1	2
f)	whooping cough	1	2
g)	urinary infection	1	2
h)	eye infection	1	2
i)	ear infection	1	2
j)	chest infection	1	2
k)	other infection (please tick and describe)	1	2

....

A19. Approximately how many times **in the last 12 months** has:

a) the family doctor come to your home because your study child was ill [ ][ ] times (put 00 if not at all)

b) the family doctor seen your study child in his surgery because she was unwell? [ ][ ] put 00 if not at all)

c) a doctor seen your study child for a routine check? [ ][ ] times (put 00 if not at all)

SECTION B: SLEEPING

B1. Does your child have a regular sleeping routine?

Yes ☐1 No ☐2

B2. a) How many hours sleep does she usually have during the day time?

none ☐1 less than 1 hour ☐2 [illegible] hours ☐3 mor[illegible] 2 hours ☐4 don't know ☐9

b) Normally what time in the evening does your child go to sleep?

hours ☐☐ minutes ☐☐ p.m.

B3. a) What time does she normally wake up in the morning?

hours ☐☐ minutes ☐☐ a.m.

b) How often during the night does she usually wake? ☐☐ times

c) How often during a normal day does she have a sleep? ☐ times

B4. a) Where does the child usually sleep?

	**(i) When she goes to bed at night**	**(ii) When she wakes in the morning**
in her own room on her own	☐1	☐1
in a room with other children	☐2	☐2
in your bedroom	☐3	☐3
in a room with other adults	☐4	☐4
other place (please tick and describe)	☐5	☐5

....
.

B4.b) Does the child sleep on her own most nights or does she share a bed or cot?

	**(i) When she goes to bed at night**	**(ii) When she wakes in the morning**
in her own bed	1	1
in a bed with other children	2	2
in your bed with you	3	3
in a bed with other adult	4	4
other place (please tick and describe)	5	5

....
.

c) How often does she sleep?

		**Always**	**Usually**	**Sometimes**	**Hardly ever**
i)	on her back	1	2	3	4
ii)	on her side	1	2	3	4
iii)	on her front	1	2	3	4

B4. d) In the room where the child sleeps most of the night:

		In Winter			In Summer		
		**Yes always**	**Yes some-times**	**No not at all**	**Yes always**	**Yes some-times**	**No not at all**
i)	is the heating on all night?	1	2	3	1	2	3
ii)	is the heating on part of the night?	1	2	3	1	2	3
iii)	is there a window open at night?	1	2	3	1	2	3
iv)	does she sleep with a duvet?	1	2	3	1	2	3
v)	does she have an electric blanket?	1	2	3	1	2	3
vi)	does she sleep with a pillow?	1	2	3	1	2	3

B5. Do you feel her sleep pattern is :

better than other children of the same age	1
same as other children of the same age	2
worse than other children of the same age	3
don’t know	9

B6. **In the past year** has your child regularly:

	**Yes, but did not worry me**	**Yes, worried me a bit**	**Yes, worried me greatly**	**No, did not happen**	**Don't know**
a) refused to go to bed	1	2	3	4	9
b) woken very early	1	2	3	4	9
c) had difficulty going to sleep	1	2	3	4	9
d) had nightmares	1	2	3	4	9
e) continued to get up after being put to bed	1	2	3	4	9
f) woken in the night	1	2	3	4	9
g) got up after only a few hours sleep	1	2	3	4	9

B7. What time in the evening do you (first) put your child to bed? hours ☐☐ minutes ☐☐ p.m.

SECTION C: YOU AND YOUR CHILD

C1. a) Do you ever have a battle of wills with your child?

never	1	→ **If never, go to C2 below**
rarely	2	
sometimes	3	
frequently	4	

b) What are they usually about:

....

....

c) Who most often wins?

me	1
she does	2
about even	3
neither of us	4

C2. How often does she refuse to go to bed?

most of the time	1
often	2
at times	3
rarely	4
never	5

C3. a) How often does she have temper tantrums?

more than once a day	1
most days	2
at least once a week	3
less than once a week	4
never	5 → **If never, go to C4 on page 24**

If she has temper tantrums:

b) Why do you think they happen? (please tick all that apply)

i)	failure to get what she wants	1
ii)	failure to make herself understood	1
iii)	reaction to being corrected	1
iv)	no particular reason	1
v)	other (please tick and describe)	1

....

c) When she has temper tantrums how often do you:

		**Often**	**Sometimes**	**Never**
i)	ignore it, let her get it out of her system	1	2	3
ii)	send her away for 'time out' eg. send her to her bedroom	1	2	3
iii)	try to hold and cuddle her	1	2	3
iv)	try to reason with her	1	2	3

C3. c)

	Often	Sometimes	Never
v) leave it for someone else to cope with	1	2	3
vi) slap or hit her	1	2	3
vii) try to distract her	1	2	3
viii) shout at her	1	2	3
ix) other (please tick and describe)	1	2	3

....

C4. How often does she do the following:

	Often	Sometimes	Never
a) repeatedly rocks her head or body for no reason	1	2	3
b) has a tic or twitch	1	2	3
c) has other unusual behaviour (please tick and describe)	1	2	3

....

C5.

**About how often does she go to:**	**Nearly every day**	**2 - 5 times a week**	**Once a week**	**Once a month**	**A few times per year**	**Never** ↓
a) local shops	1	2	3	4	5	6
b) department store	1	2	3	4	5	6
c) supermarket	1	2	3	4	5	6
d) park or playground	1	2	3	4	5	6

		Nearly every day	2 - 5 times a week	Once a week	Once a month	A few times per year	Never
C5.e)	visits to friends	1	2	3	4	5	6
f)	visits to relatives	1	2	3	4	5	6
g)	library	1	2	3	4	5	6
h)	places of interest (e.g. Zoo, museum)	1	2	3	4	5	6
i)	places of entertainment (e.g. funfair)	1	2	3	4	5	6
j)	swimming pool or other sporting area	1	2	3	4	5	6

C6. How much choice do you allow her in deciding what foods she eats at meals?

	Main meal	Snacks
she can choose from any food available	1	1
she is given a choice from a few alternatives that an adult chooses	2	2
an adult decides what she will eat	3	3

C7. Do you allow her to choose what clothes she will wear?

she always takes part in choosing	1
she has some choice	2
she has no choice in what she will wear	3

C8. Does your child have:

		Yes	No
a)	cuddly toys	1	2
b)	construction toys (e.g. lego)	1	2
c)	co-ordination toys (eg. set of blocks, shape posting box, stacking cups)	1	2
d)	jigsaw puzzles	1	2
e)	action dolls (e.g. Barbie, Power Ranger)	1	2
f)	computer games	1	2
g)	toy cars	1	2

C9. About how many books does she have of her own or that she shares with brothers or sisters?

none	1
1 - 2 books	2
3 - 9 books	3
10 or more	4

C10. How often do you talk to her while you do housework or are occupied in some other way?

never 1 rarely 2 sometimes 3

often 4 almost always 5

C11. a) Do you have a television set?

Yes 1 No 2 → **If <u>no</u>, go to C12 on page 28**

If yes,

C11. b) How often is your child allowed to watch the television or a video?

whenever she wants	1	
when I decide it is suitable	2	
hardly ever	3	→**If hardly ever, go to C12 on page 28**

c) When do you normally have the television on?

	Weekdays	Weekends
all day	1	1
most of the day	2	2
½ the day	3	3
less than ½ the day	4	4
not at all	5	5

d) Does your child watch television or a video when it is on?

		**Always**	**Sometimes**	**Never**
i)	yes, but plays at the same time	1	2	3
ii)	yes, and pays attention	1	2	3
iii)	no, she ignores it	1	2	3

If she does watch TV,

C11. e) what programmes does she see? (tick all that apply)

			**Don't know**
i)	children's programmes	1	9
ii)	other programmes	1	
iii)	children's videos	1	
iv)	other videos	1	

C11. f) About how close to the TV does she usually sit?

less than 1 metre [1] about 1 metre [2] more than 1 metre [3]

varies [4]

C12. How often does she play with other children (other than brothers or sisters)?

every day [1]

2 - 6 times a week [2]

once a week [3]

less than once a week [4]

never [5]

C13. When you and your child meet again after being apart for an hour or more, does she tell you what she's been doing?

yes, always [1] yes, sometimes [2] hardly ever [3]

never [4] we are never apart [7]

C14. a) Many children have particular types of activities that they prefer or toys they play with.
How often has your daughter played with the following in the past month:

	Plays with:	Never	Hardly ever	Some-times	Often	Very often
i)	Guns (or objects used as guns)	1	2	3	4	5
ii)	Jewellery	1	2	3	4	5
iii)	Tool set	1	2	3	4	5
iv)	Dolls	1	2	3	4	5

C14.a) **Plays with:**		**Never**	**Hardly ever**	**Some-times**	**Often**	**Very often**
v)	Trains, cars or aeroplanes	1	2	3	4	5
vi)	Swords (or objects used as swords)	1	2	3	4	5
vii)	Tea set	1	2	3	4	5

C14. b) How often in the past month has she done the following:

		**Never**	**Hardly ever**	**Some-times**	**Often**	**Very often**
i)	Played house (e.g. cleaning, cooking)	1	2	3	4	5
ii)	Played with girls	1	2	3	4	5
iii)	Pretended to be a female person (e.g. a princess)	1	2	3	4	5
iv)	Pretended to be a male character (e.g. a soldier)	1	2	3	4	5
v)	Played at fighting	1	2	3	4	5
vi)	Played at being a mother or father	1	2	3	4	5
vii)	Played ball games	1	2	3	4	5
viii)	Climbed (fence, tree, climbing frame)	1	2	3	4	5
ix)	Played at looking after babies	1	2	3	4	5

C14. b) How often in the past month has she done the following:

		Never	Hardly ever	Some-times	Often	Very often
x)	Showed interest in real cars, trains and aeroplanes	1	2	3	4	5
xi)	Dressed up in girlish clothes	1	2	3	4	5
xii)	Played with boys	1	2	3	4	5

C14. c) How often does she:

		Never	Hardly ever	Some-times	Often	Very often
i)	Like to explore new surroundings	1	2	3	4	5
ii)	Enjoy rough and tumble play	1	2	3	4	5
iii)	Show interest in spiders, insects or snakes	1	2	3	4	5
iv)	Avoid getting dirty	1	2	3	4	5
v)	Like pretty things	1	2	3	4	5
vi)	Avoid taking risks	1	2	3	4	5

C15. Do you feel that she dominates the household?

Yes, usually	Yes, sometimes	No, not at all
1	2	3

C16. Do you start by being firm but then give way?

Yes, usually	Yes, sometimes	No, not at all
1	2	3

C17. Space for comments:

....

....

C18. How often does your partner do these activities with your child?

**Partner:**	**Often**	**Sometimes**	**Rarely**	**Never**	**Have no partner**
a) gives her a bath or shower	1	2	3	4	7 → **Go to C19 on page 32**
b) makes things with her	1	2	3	4	
c) sings to her	1	2	3	4	
d) reads to her	1	2	3	4	
e) plays with toys	1	2	3	4	
f) cuddles her	1	2	3	4	
g) active play (eg. ball games, hide and seek)	1	2	3	4	
h) takes her to a park or playground	1	2	3	4	
i) puts her to bed	1	2	3	4	
j) takes her swimming	1	2	3	4	
k) draws or paints with her	1	2	3	4	
l) prepares food for her	1	2	3	4	
m) other (please tick and describe)	1	2	3	4	....

C19. How often do you do these activities with your child?

**You:**	**Often**	**Sometimes**	**Rarely**	**Never**
a) bath (or shower) her	1	2	3	4
b) make things with her	1	2	3	4
c) sing to her	1	2	3	4
d) read to her	1	2	3	4
e) play with toys	1	2	3	4
f) cuddle her	1	2	3	4
g) active play (eg. ball games, hide and seek)	1	2	3	4
h) take her to the park or playground	1	2	3	4
i) put her to bed	1	2	3	4
j) take her swimming or other activity	1	2	3	4
k) draw or paint with her	1	2	3	4
l) prepare food for her	1	2	3	4
m) other (please describe)	1	2	3	4

....

C20. About how often does your child do the following:

		Every day	2-6 times a week	once a week	once a month	not at all
a)	Go to a park or playground	1	2	3	4	5
b)	Go swimming	1	2	3	4	5
c)	Play a musical instrument (e.g. piano, recorder)	1	2	3	4	5
d)	Go to a library	1	2	3	4	5
e)	Go to special groups (such as Beavers or Rainbows)	1	2	3	4	5

Please tick and describe ....

		Every day	2-6 times a week	once a week	once a month	not at all
f)	Go to Sunday school	1	2	3	4	5
g)	Go to special classes or clubs for some activity (e.g. dancing, judo, sports)	1	2	3	4	5

Please tick and describe...

		Every day	2-6 times a week	once a week	once a month	not at all
h)	Go to special classes because of learning difficulty	1	2	3	4	5

Please tick and describe...

		Every day	2-6 times a week	once a week	once a month	not at all
i)	Have physiotherapy	1	2	3	4	5
j)	See her grandparents	1	2	3	4	5
k)	Play computer games	1	2	3	4	5
l)	Help in the house	1	2	3	4	5

C21. Has she had her ears pierced ? Yes 1 No 2

SECTION D: UPSETTING EVENTS

Below are listed some events that might upset some children. Please state whether any of these has happened since she was 3 years old.

Since her 3rd birthday:		Yes and she was very upset	Yes and she was quite upset	Yes and she was a bit upset	Yes but she wasn't upset	No did not happen
D1.	She was taken into care*	1	2	3	4	5
D2.	A pet died	1	2	3	4	5
D3.	She moved home	1	2	3	4	5
D4.	She had a shock or fright*	1	2	3	4	5
D5.	She was physically hurt by someone*	1	2	3	4	5
D6.	She was sexually abused*	1	2	3	4	5
D7.	She was separated from her mother*	1	2	3	4	5
D8.	She was separated from her father*	1	2	3	4	5
D9.	She acquired a new mother or father*	1	2	3	4	5
D10.	She had a new brother or sister	1	2	3	4	5
D11.	She was admitted to hospital	1	2	3	4	5
D12.	She changed care taker (i.e. the person mostly looking after her)	1	2	3	4	5

**Since her 3rd birthday:**	**Yes and she was very upset**	**Yes and she was quite upset**	**Yes and she was a bit upset**	**Yes but she wasn't upset**	**No did not happen**
D13. She was separated from someone else*	1	2	3	4	5
D14. She started a new nursery or kindergarten	1	2	3	4	5
D15. She started school	1	2	3	4	5
D16. Something else*	1	2	3	4	5

If yes, to any marked *, please give details below:

....

....

SECTION E: ABILITIES AND DISABILITIES

Children in this study have a range of skills and abilities and some have a number of disabilities. These questions will enable us to get a picture of your child. Please answer each question: If you don't know the answer ask your child to do the task.

E1.		**Yes, can do well**	**Yes, does but not very well**	**Has not yet done**	**Is unable to try this**
a)	Is she is able to walk?	1	2	3	7
b)	Is she able to stoop down and pick up something from the floor?	1	2	3	7
c)	Is she able to run?	1	2	3	7
d)	Can she jump forward with both feet together?	1	2	3	7
e)	Can she walk on tiptoe?	1	2	3	7
f)	Can she run on tiptoe?	1	2	3	7
g)	Can she hop on one foot for 3 steps?	1	2	3	7
h)	Can she walk backwards for 4 steps?	1	2	3	7
i)	Can she stand on 1 foot for at least 8 seconds?	1	2	3	7
j)	Can she walk upstairs, putting both feet on each step?	1	2	3	7
k)	Can she walk upstairs, putting one foot on each step?	1	2	3	7
l)	Can she walk downstairs, putting both feet on each step?	1	2	3	7
m)	Can she walk downstairs, putting one foot on each step?	1	2	3	7

E1.		Yes, can do well	Yes, does but not very well	Has not yet done	Is unable to try this
n)	Can she run upstairs?	1	2	3	7
o)	Can she ride a tricycle?	1	2	3	7
p)	Can she ride a bicycle?	1	2	3	7
q)	Can she swim with waterwings?	1	2	3	7
r)	Can she swim without waterwings?	1	2	3	7
s)	Can she do a handstand against the wall?	1	2	3	7
t)	Can she skip with a skipping rope?	1	2	3	7
u)	Can she stand on her head?	1	2	3	7

E2.

a)	Can she hold a pencil and scribble?	1	2	3	7
b)	Can she copy a vertical line with a pencil?	1	2	3	7
c)	Can she wiggle her thumb?	1	2	3	7
d)	Can she draw a circle (more or less)?	1	2	3	7
e)	Can she bang together two objects that she is holding?	1	2	3	7
f)	Can she draw (or copy) a cross?	1	2	3	7

E2.		Yes, can do well	Yes, does but not very well	Has not yet done	Is unable to try this
g)	Can she draw (or copy) a square?	1	2	3	7
h)	Can she write her name?	1	2	3	7
i)	Can she write any numbers?	1	2	3	7

j) If you ask her to draw a man, what is the result likely to be?

just a scribble 1 a head and not much else 2 a head and body 3

a head, body and legs 4 head, body, arms, legs 5

other (please describe) 6 ....

E3.		Yes, can do well	Yes, does but not very well	Has not yet done	Is unable to try this
a)	Can she pick up a small object using finger and thumb only?	1	2	3	7
b)	Can she turn the pages of a book?	1	2	3	7
c)	Can she build a tower putting one object on top of another?	1	2	3	7
d)	Can she build a tower of 4 bricks?	1	2	3	7
e)	Can she build a tower of 6 bricks?	1	2	3	7
f)	Can she build a tower of 8 bricks?	1	2	3	7
g)	Can she put bricks together to make a bridge?	1	2	3	7

E4.		Yes, can do well	Yes, does but not very well	Has not yet done	Is unable to try this
a)	Does she show interest in pictures in books?	1	2	3	7
b)	Does she notice details in pictures and photographs?	1	2	3	7
c)	Can she recognise the colours red, yellow and blue?	1	2	3	7
d)	Can she recognise orange, brown and purple?	1	2	3	7
e)	Can she recognise her name when written?	1	2	3	7
f)	Does she know at least 3 letters of the alphabet?	1	2	3	7
g)	Does she know at least 10 letters of the alphabet?	1	2	3	7
h)	Can she read simple words ?	1	2	3	7
i)	Can she read a story with less than 10 words a page?	1	2	3	7
j)	Can she read a story with more than 10 words a page?	1	2	3	7
k)	Does she understand numbers 1 and 2?	1	2	3	7
l)	Does she understand numbers 3 and 4?	1	2	3	7
m)	Does she understand numbers 5 to 10?	1	2	3	7
n)	Can she count up to 20?	1	2	3	7
o)	Can she count up to 100?	1	2	3	7

E5.		Yes, can do well	Yes, does but not very well	Has not yet done	Is unable to try this
a)	Does she share her toys with other children?	1	2	3	7
b)	Does she share the toys of other children, understanding that they are not hers?	1	2	3	7
c)	Does she feel sympathy for someone if they are hurt?	1	2	3	7
d)	Does she think of things to do to please you?	1	2	3	7
e)	Can she kick a large ball?	1	2	3	7
f)	Can she throw a small ball underarm?	1	2	3	7
g)	Can she throw a small ball overarm?	1	2	3	7
h)	Can she throw a ball against a wall and catch it?	1	2	3	7
i)	Does she take turns in a game without fuss?	1	2	3	7
j)	Can she play card games (e.g. snap) ?	1	2	3	7
k)	Can she play any board games?	1	2	3	7

E6.		Yes, can do well	Yes, does but not very well	Has not yet done	Is unable to try this
a)	Does she drink from a cup or mug?	1	2	3	7
b)	Does she eat skilfully with a spoon?	1	2	3	7
c)	Does she eat with fork and spoon in each hand?	1	2	3	7
d)	Does she cut her food with a knife?	1	2	3	7
e)	Can she sit at table and cope with a whole meal without help?	1	2	3	7
f)	Can she wash and dry her hands on her own?	1	2	3	7
g)	Can she brush her teeth on her own?	1	2	3	7
h)	Can she get dressed without help?	1	2	3	7
i)	Can she get undressed without help?	1	2	3	7
j)	Can she do up buttons?	1	2	3	7
k)	Can she tie a bow?	1	2	3	7
l)	Can she brush and comb her hair?	1	2	3	7

E7.		Yes, can do well	Yes, does but not very well	Has not yet done	Is unable to try this
a)	Can she listen to a short story from start to finish?	1	2	3	7
b)	Can she understand instructions such as: 'Find the jumper that Granny gave you'?	1	2	3	7
c)	Does she talk clearly?	1	2	3	7
d)	Does she sing songs (even if the words are not clear)?	1	2	3	7
e)	Does she ask sensible questions?	1	2	3	7
f)	Can she carry on a conversation?	1	2	3	7
g)	Can she say at least 3 nursery rhymes?	1	2	3	7
h)	Can she sing at least 3 songs?	1	2	3	7
i)	Can she hum a tune?	1	2	3	7
j)	Can she beat a rhythm by clapping hands in time to the music?	1	2	3	7

E8.		Never	Sometimes	Often	Always
a)	Does she stumble or get stuck on words, or repeat them many times? (e.g. I I I I want a sweet)	1	2	3	7
b)	Is her voice hoarse or husky?	1	2	3	7
c)	Can you understand what she says?	1	2	3	7
d)	Can your family understand what she says?	1	2	3	7
e)	Can visitors to your house understand what she says?	1	2	3	7

E9.a) Does she prefer to use gestures (pointing or pulling) to get what she wants instead of asking?

Yes, still does 1

Yes, did in past, not now 2

No, never did 3

b) When she talks nowadays, what is the most words she can put together (e.g. "I want juice" would be 3).

one word 1 two words 2 3 or 4 words 3 5 -8 words 4 9 or more words 5

does not talk at all 6

E10. Some children enjoy talking and others do not.

**Does your child:**	**Always**	**Sometimes**	**Never**
a) talk a lot	1	2	3
b) stay mainly silent	1	2	3
c) seem to avoid looking at people's faces when she talks	1	2	3
d) echo what has just been said to her (e.g. you say; 'we are going out now' she says: 'going out now'.)	1	2	3

E11. a) Does your daughter have difficulty in pronouncing certain sounds (e.g. th, sss, t)?

Yes 1 No 2

b) **If <u>yes</u>,** please describe ....

E12. a) Are there any other languages apart from English spoken in your household?

Yes 1 No 2 → **If <u>no</u>, go to E13 on page 45**

If <u>yes</u>, please say which ....

....

E12. b) Is English the main language spoken?

	(i) By mother	(ii) By study child	(iii) By partner	(iv) By other children
English is the main language spoken	1	1	1	1
both English and other language used equally	2	2	2	2
other is the main language spoken	3	3	3	3
no such person at home	7		7	7

Space for comments.

E13. Are you worried about any aspects of your child's growth and development?

		Yes I am worried	No I am not worried
a)	her speech	1	2
b)	her weight	1	2
c)	her height	1	2
d)	her behaviour	1	2
e)	her general development	1	2
f)	other	1	2

If yes, to any of these, please describe what worries you:

....

....

....

This is confidential information. We cannot make any response to what you put. If you are worried about your child's development we suggest you contact your family doctor or your health visitor.

SECTION F: TEMPERAMENT AND BEHAVIOUR

How often is your child's behaviour like that given below:

		Never	Rarely	Some-times	Often	Always
F1.	She tends to be shy	1	2	3	4	5
F2.	She cries easily	1	2	3	4	5
F3.	She likes to be with people	1	2	3	4	5
F4.	She is always on the go	1	2	3	4	5
F5.	She prefers playing with others rather than alone	1	2	3	4	5
F6.	She is somewhat emotional	1	2	3	4	5
F7.	When she moves about she moves slowly	1	2	3	4	5
F8.	She makes friends easily	1	2	3	4	5
F9.	She is off and running as soon as she wakes up in the morning	1	2	3	4	5
F10.	She finds people more stimulating than anything else	1	2	3	4	5
F11.	She fusses and cries	1	2	3	4	5

		Never	Rarely	Some-times	Often	Always
F12.	She is very sociable	1	2	3	4	5
F13.	She is very energetic	1	2	3	4	5
F14.	She takes a long time to warm to strangers	1	2	3	4	5
F15.	She gets upset easily	1	2	3	4	5
F16.	She is something of a loner	1	2	3	4	5
F17.	She prefers quiet inactive games to more active ones	1	2	3	4	5
F18.	When alone she feels isolated	1	2	3	4	5
F19.	She reacts intensely when upset	1	2	3	4	5
F20.	She is very friendly with strangers	1	2	3	4	5
F21.	She bullies other children	1	2	3	4	5
F22.	She is very restless, hardly ever still.	1	2	3	4	5
F23.	She is squirmy or fidgety	1	2	3	4	5
F24.	She destroys her own things or those belonging to others	1	2	3	4	5

		Never	Rarely	Some-times	Often	Always
F25.	She fights with other children	1	2	3	4	5
F26.	She is not much liked by other children	1	2	3	4	5
F27.	She worries about many things	1	2	3	4	5
F28.	She does things on her own. She is rather solitary	1	2	3	4	5
F29.	She is irritable. Is quick to fly off the handle	1	2	3	4	5
F30.	She appears miserable, unhappy, tearful or distressed	1	2	3	4	5
` F31.	She takes things belonging to others	1	2	3	4	5
F32.	She bites her nails or fingers	1	2	3	4	5
F33.	She is disobedient	1	2	3	4	5
F34.	She cannot settle to do anything for more than a few moments	1	2	3	4	5
F35.	She is afraid of new things or new situations	1	2	3	4	5

		Never	Rarely	Some-times	Often	Always
F36.	She is fussy or over- particular	1	2	3	4	5
F37.	She tells lies	1	2	3	4	5
F38.	She likes to sit and watch TV rather than play active games	1	2	3	4	5
F39.	She laughs a lot	1	2	3	4	5
F40.	She smiles when she sees her parent(s)	1	2	3	4	5
F41.	She likes a cuddle	1	2	3	4	5
F42.	She really enjoys life	1	2	3	4	5

F43. How often does she join in with others?

Never 1 Often 2 Sometimes 3

F44. How often does she find it hard to wait for her turn in a game?

Never 1 Sometimes 2 Often 3 Always 4

F45. How many children choose to play with her?

None 1 1 or 2 2 3 or more 3

F46. How upset does she get over quite small things? (e.g. breaking things, getting dirty hands, falling over?)

Not at all 1 Sometimes 2 Often 3 Nearly always 4

F47. How easy is it to comfort her when she is upset?

Very easy [1] Quite easy [2] Quite hard [3] Very hard [4]

F48. How often will she comfort another child who is upset, or get someone else to help?

Never [1] Sometimes [2] Often [3] Always [4]

F49. How often does she tell you things about others that **you know are not true** to get **them into trouble**.

Never [1] Sometimes [2] Often [3] Always [4]

F50. How often, when you ask her to do something does she do it straight away?.

Never [1] Sometimes [2] Often [3] Always [4]

F51. How good would you describe her at sitting still (for a meal or story)?

Is very active - doesn’t sit still when she should [1]

Can usually sit still when she should [2]

Can sit still for a long time [3]

Is very inactive [4]

F52. How long can she concentrate on a game or task you have given her to do?

Up to 2 minutes [1] 2-5 minutes [2] 5-10 minutes [3]

10-15 minutes [4] More than 15 minutes [5]

F53. How often is she too demanding of you? (e.g. asking for help for a task she can do herself)

Less than once a month [1] Once a month [2] Once a week [3]

Once a day [4] More than once a day [5]

F54. How often, does she whine or moan with little reason?

Less than once a month [1] Once a month [2] Once a week [3]

Once a day [4] Two or three times a day [5]

F55. How often, is she unhappy for no apparent reason?

Less than once a month [1] Once a month [2] Once a week [3]

Once a day [4] Two or three times a day [5]

F56. How often, if you leave the room does she want to follow you?

Never [1] Sometimes [2] Often [3] Always [4]

		**No not at all**	**Yes sometimes**	**Yes often**	**Yes always**
F57.	Does she ever refuse to do as she's told by other adults (not her parents)?	1	2	3	4
F58.	Does she interrupt, butt in, when others are talking	1	2	3	4
F59.	Does she talk far too much?	1	2	3	4

F60. How often do you understand what she says?

Never [1] Occasionally [2] About half the time [3]

Most of the time [4] Always [5]

F61. When you tell her something how often does she try to understand?

Never [1] Sometimes [2] Often [3] Always [4]

F62. a) Does your study child have brothers or sisters?

Yes [1] No [2] → **If no, go to F63a on page 54**

If yes:

**How often:**	**Never**	**Sometimes**	**Often**	**Not sure**
b) Is she teased by them?	1	2	3	9
c) Does she tease them?	1	2	3	9
d) Is she deliberately provoked by them?	1	2	3	9
e) Does she deliberately provoke them?	1	2	3	9
f) Is she willing to share things with them?	1	2	3	9
g) Is she interrupted by them when she is working or playing?	1	2	3	9

F63. a) Does your study child have contact with other children?

Yes [1] No [2] → **If no, go to F64 below**

If yes:

**How often:**	**Never**	**Sometimes**	**Often**	**Not sure**
b) Is she teased by them?	1	2	3	9
c) Does she tease them	1	2	3	9
d) Is she deliberately provoked by them?	1	2	3	9
e) Does she deliberately provoke them?	1	2	3	9
f) Will she share things with them?	1	2	3	9
g) Is she interrupted by them when she is doing somthing?	1	2	3	9

F64. Please tick any of the following activities your child willingly undertakes.

a) Handicraft (e.g. painting, drawing, making things) [1]

b) Playing on mobile toys (e.g. cycle, scooter, ride on trucks [1]

c) Music (e.g. listening, singing, dancing) [1]

d) Climbing on things (e.g. climbing frame, low walls) [1]

e) Constructive toys (e.g. lego) [1]

f) Imaginative play (pretend games) [1]

F64. g) Messy play (e.g. sand, water, mud) ☐1

h) Investigative play (finding out how things work) ☐1

i) Other (please tick and describe) ☐1

....

F65. How often does she speak freely when she is with adults?

	Never	Sometimes	Often	Nearly always	Not sure
a) Adults who are family	1	2	3	4	9
b) Other adults	1	2	3	4	9

F66. How often does she speak freely when she is with children?

	Never	Sometimes	Often	Nearly always	Not sure	No such children
a) Children who are family	1	2	3	4	9	7
b) Other children	1	2	3	4	9	

F67. How often does she:

	Never	Sometimes	Often	Don't know
a) Deliberately tear things (e.g wallpaper, books)	1	2	3	9
b) Deliberately break toys	1	2	3	9
c) Wander about aimlessly	1	2	3	9
d) Stare into space	1	2	3	9
e) Seem to have nothing to do	1	2	3	9

F68. When you **take her out somewhere**, how often does she behave in the ways listed below? (Please put a tick against EACH listed behaviour)

	**Never**	**Sometimes**	**Often**	**Always**
a) Does what *you* ask	1	2	3	4
b) Runs off	1	2	3	4
c) Has a tantrum	1	2	3	4
d) Is fun to be with	1	2	3	4
e) Is interested in what is going on	1	2	3	4
f) Is aware of the reactions of others	1	2	3	4

F69. When trying to tell you something, does she:

		**Yes**	**No**
a)	Have a stutter or stammer ?	1	2
b)	Have a lisp?	1	2
c)	Say things that do not make sense?	1	2

F70. How often does she do the things listed below to **family members**?

	FAMILY					
	(i) ADULTS			(ii) CHILDREN		
**Does she:**	**Never**	**Sometimes**	**Often**	**Never**	**Sometimes**	**Often**
a) Kick them	1	2	3	1	2	3
b) Hit them	1	2	3	1	2	3
c) Fight them	1	2	3	1	2	3
d) Swear at them	1	2	3	1	2	3
e) Use angry words	1	2	3	1	2	3

F71. As far as you know, how often does she do the things below to people who are **not family members**?

	NOT FAMILY					
	(i) ADULTS			(ii) CHILDREN		
	**Never**	**Sometimes**	**Often**	**Never**	**Sometimes**	**Often**
a) Kick them	1	2	3	1	2	3
b) Hit them	1	2	3	1	2	3
c) Fight them	1	2	3	1	2	3
d) Swear at them	1	2	3	1	2	3
e) Use angry words	1	2	3	1	2	3

F72. Most children have some fears. Is your child ever afraid of:

		Yes	No	Has not experienced this
a)	Haircut	1	2	7
b)	Using the toilet	1	2	7
c)	Doctors	1	2	7
d)	Hospital	1	2	7
e)	TV programmes	1	2	7
f)	Vacuum cleaner	1	2	7
g)	Being alone	1	2	7
h)	Stories	1	2	7
i)	Dogs	1	2	7
j)	Cats	1	2	7
k)	Other animals	1	2	7
l)	Insects	1	2	7
m)	Thunder/loud noises	1	2	7
n)	The dark	1	2	7
o)	Strangers	1	2	7
p)	Going out	1	2	7
q)	Car, bus, train, tube train	1	2	7
r)	Lift, escalator	1	2	7
s)	Water, the bath	1	2	7
t)	Getting dirty	1	2	7
u)	Other (please tick and describe)	1	2	7

....

F73. How often does she do the following things?

	Never	Sometimes	Often
a) Wet the bed when she is ill	1	2	3
b) Wet the bed when she is nervous or excited about something	1	2	3
c) Wet the bed when there is no apparent reason	1	2	3

F74. As far as you know, how often does she take things and keep them without permission?

	Never	Sometimes	Often	Not sure
a) Takes from children in the family	1	2	3	9
b) Takes from other children	1	2	3	9
c) Takes from adults in the family	1	2	3	9
d) Takes from other adults	1	2	3	9

F75. Does your child show any other behaviours that cause problems?

Yes 1 No 1

If <u>yes</u>, please describe ....

....

....

F76. Space for you to describe the strong points of her behaviour.

....

....

SECTION G

G1. This questionnaire was completed by: (tick all that apply)

a) mother [ ] 1

b) father [ ] 1

c) other (please describe) [ ] 1 ....

G2. Please give the date on which you completed this questionnaire:

day		month		year			
				1	9	9	

G3. Please give the date of birth of your child:

day		month		year			
				1	9	9	

THANK YOU VERY MUCH FOR YOUR HELP

Space for any additional comments you would like to make

NB. Please remember that we cannot respond personally to your comments unless they are signed.

When completed, please return the questionnaire to:

Professor Jean Golding
Children of the Nineties - ALSPAC
Institute of Child Health
24 Tyndall Avenue
Bristol
BS8 1BR Tel: Bristol 9285007

For office use only:

Coder		Int	
