# Supplementary material for: Breastfeeding, prenatal depression and children’s IQ and behaviour: a test of a moderation model
Source: BMC Pregnancy Childbirth. 2021 Jan 18;21:62. doi: 10.1186/s12884-020-03520-8 (PMC7814604; doi:10.1186/s12884-020-03520-8)
Supplement: Supplementary file 6 — Additional file 6:. ques-cb01-my-young-baby-girl. Questionnaire data collected at 4 weeks postpartum including infant feeding information used in this study. [file 12884_2020_3520_MOESM6_ESM.pdf]

© University of Bristol

M Y Y O U N G B A B Y

G I R L

This questionnaire is all about your baby. It asks about any problems with feeding and sleeping, waking and crying. Remember, there are no right or wrong answers. We are interested in her health and how she behaves.

We look forward to hearing from you.

THANK YOU VERY MUCH FOR YOUR HELP

31/01/92

Recycled Paper

FILLING IN THIS BOOKLET

Most of the questions can be answered by ticking the box beside the right answer.

For example

How many times have you been to the supermarket in the past week?

None $_1$ 1$_2$ 2-6$_3$ 7 or more $_4$

This means you went to the supermarket once in the past week

Sometimes there are questions with if in front of them.

For example

a) Have you been to the supermarket today?

Yes $_1$ No $_2$

This means you didn't go to the supermarket and you don't need to answer the next question

b) If yes, did you buy any carrots?

Yes $_1$ No $_2$

In general, though, each question needs an answer.

In some questions you may be asked to describe something.
It would be helpful if you wrote as clearly as possible.

The small numbers in the squares are for office use only.

SECTION A:YOU AND YOUR BABY

A1. As soon as the baby was born:

I wanted to touch and feel her	1
I didn't feel strongly about her	2
I wanted to see her later and enjoy her when I had recovered	3
I felt I didn't want to see her	4
None of these	5

A2. How soon after delivery was the baby put to your breast? (please tick one box)

immediately	1	within an hour	2
1 - 3 hours	3	4 - 11 hours	4
12 hours or more	5	did not choose to put to breast	6
was not able to put baby to breast	7	don't remember	9

A3. During the first 2 days after she was born, was your baby next to you?

	**(i) during day**	**(ii) during night**
yes, all of the time	1	1
yes, most of the time	2	2
yes, some of the time	3	3
no, not at all	4	4

A4. a) Was your baby admitted to a special care baby unit or neonatal intensive care unit or other hospital ward?

Yes 1 No 2 Unsure 3

If yes please give reasons:.................................

....

....

A5. a) After coming home was your baby admitted to hospital at all?

Yes 1 No 2 Has never left hospital 7

If no, or has never left hospital, go to B1.

If yes,

b) What was the reason: ....

....

....

c) What was the name of the hospital?

....

d) How long did she stay in hospital? ....

less than $_1$<br>1 day	1-2 days$_2$	3-6 days$_3$	more than$_4$<br>6 days

e) Did you also stay in hospital at the same time?

Yes $_1$ No $_2$

f) What treatment was given? ....

....

....

SECTION B:FEEDING

B1. How have you fed your baby since she was born? Please indicate for each of the times given.

		Breast only	Bottle only	Breast & bottle	Other (please describe below)
a)	First 24 hours	1	2	3	4
b)	Rest of 1st week	1	2	3	4
c)	2nd week	1	2	3	4
d)	3rd week	1	2	3	4
e)	4th week	1	2	3	4

....

If you have never fed by bottle, go to B3.

B2. Which types of bottle milk have you used? Please indicate the brands and how long you used them for this baby.

		No	Yes	If yes, for how long:
a)	SMA Gold	1	2	....
b)	SMA White	1	2	....
c)	Cow & Gate Plus	1	2	....
d)	Cow & Gate Premium	1	2	....
e)	Farley's Oster Milk	1	2	....
f)	Oster Milk 2	1	2	....
g)	Farley's Junior	1	2	....
h)	Other (please describe)	1	2	....

....

B3. Is your baby fed (either by breast or bottle) on a regular schedule (e.g. every 4 hours)?

yes always	1
yes try to	2
no, fed on demand	3

B4. a) How is your baby being fed at the moment?

breast	1
Bottle	2
breast and bottle	3
other (please describe)	4

....

....

b) Does she have any of the following now?

	No	Yes	If yes give make(s):
i) fruit juice	1	2	....
ii) vitamins	1	2	....
iii) glucose solution	1	2	....
iv) cereals	1	2	....
v) other	1	2	....

B4. c) How often do you give her a bottle of:

	Every day	Sometimes	Never
i) tea	1	2	3
ii) coffee	1	2	3
iii) water	1	2	3

B5. Please indicate if your baby has had the following feeding behaviours.

	Yes always	Yes sometimes	Yes only once or twice	No not at all	Don't know
a) weak sucking	1	2	3	4	9
b) choking	1	2	3	4	9
c) dribbling	1	2	3	4	9
d) drinking too fast	1	2	3	4	9
e) becoming very tired/ exhausted with feeding	1	2	3	4	9
f) slow feeding	1	2	3	4	9
g) taking only small quantities at each feed	1	2	3	4	9
h) hungry/not satisfied	1	2	3	4	9
i) refusing to take milk	1	2	3	4	9
j) has a lot of wind	1	2	3	4	9

B6. Do you feel your baby is difficult to feed?

yes, very difficult	1
yes, quite difficult	2
no, not difficult	3

B7. How often is your baby fed in the following ways:

		Always	Often	Some-times	Never	Don't know
a)	lying with bottle propped up (eg with a pillow)	1	2	3	4	9
b)	baby lying down with bottle held by you or someone else	1	2	3	4	9
c)	fed with a bottle while held in someone's arms	1	2	3	4	9
d)	breast fed	1	2	3	4	9

B8. Does your baby have a dummy or comforter?

		Usually	Often	Some-times	Never	Don't know
a)	at night	1	2	3	4	9
b)	during day	1	2	3	4	9

B9. Does your partner ever feed the baby during the night?

no	1
yes sometimes	2
yes often	3
yes always	4
have no partner	7

SECTION C:SLEEPING

C1. How often does your baby **usually** wake at night?

Never	1	
occasionally	2	
most nights	3	
once every night	4	
more than once per night	5	How many times per night...........
don't know	9	

C2. In what position is your baby: (tick all that apply)

		**(i) Lying on her back**	**(ii) Lying on her side**	**(iii) Lying on her front**
a)	when she goes down for the night?	1	1	1
b)	when she wakes up?	1	1	1

C3. When your baby wakes at night what do you do?

		**Always**	**Usually**	**Some-times**	**Never**	**Hasn't come home yet**
a)	feed her	1	2	3	4	7
b)	give drink of water	1	2	3	4	7
c)	rock or cuddle her	1	2	3	4	7
d)	give her a dummy	1	2	3	4	7
e)	bring her into your bed	1	2	3	4	7
f)	change her nappy	1	2	3	4	7
g)	other (please describe)	1	2	3	4	7

....

C4. Do you ever wake your baby for a feed?

Yes 1 No 2

C5. a) Where does the baby sleep?

	**(i) When she goes down at night**	**(ii) When she wakes at the end of the night**
in her own room on her own	1	1
with other children	2	2
in your bedroom	3	3
other place (please describe)	4	4

....

b) In what does she sleep:

	(i) When she goes down at night	(ii) When she wakes at the end of the night
cradle	1	1
carry cot	2	2
your bed	3	3
pram	4	4
cot	5	5
moses basket	6	6
something else (please describe)	7	7

....

C5. c) In the room where the baby sleeps most of the night:

	Yes always	Yes sometimes	No not at all
i) is the heating on at night?	1	2	3
ii) is there a window open at night?	1	2	3
iii) does she sleep with a duvet?	1	2	3
iv) does she have an electric blanket on?	1	2	3
v) does she sleep with a pillow?	1	2	3
vi) does she sleep in a baby nest?	1	2	3

C6. During a normal night, how many layers .... of blanket would she have?

C7. How often at night does she wear:

	Yes always	Yes sometimes	No not at all
i) vest	1	2	3
ii) babygro	1	2	3
iii) nightie	1	2	3
iv) pyjamas	1	2	3
v) cardigan or jumper	1	2	3
vi) sleepsuit	1	2	3
vii) bonnet	1	2	3
viii) mittens/gloves	1	2	3
ix) bootees	1	2	3
x) other (describe)	1	2	3

....

SECTION D:CRYING

D1. We are interested in the pattern of your baby's crying during a day.
How much does your baby cry at the following times:

		Yes always	Yes often	Yes some-times	Hardly ever	Don't know
a)	mornings	1	2	3	4	9
b)	afternoon (before 5pm)	1	2	3	4	9
c)	in the late afternoon/ evenings (5 pm onwards)	1	2	3	4	9
d)	during the night	1	2	3	4	9
e)	other (please describe)	1	2	3	4	9

....

D2. a) Does your baby ever have times when she appears to be in agony, screams, draws her legs up to her body and can't be calmed?

yes often 1 yes sometimes 2 yes once only 3

no 4 don't know 9

If no, or don't know, go to D3.

If yes,

b) does this tend to happen at a particular time of day?

yes 1 no 2 can't say 3

If yes,

i) at which time of day? ....

c) Have you noticed whether anything brings these attacks on?

yes 1 no 2 can't say 3

If yes,

i) please describe: ....

d) How long do these attacks usually last?

few minutes 1 less than 1 hour 2

1 - 2 hours 3 more than 2 hours 4

D3. How much do you feel that your baby cries in comparison with other babies of her age?

she cries more than other babies 1

she is the same as other babies 2

she cries less than other babies 3

don't know 9

D4. a) Do you feel that your child's crying is a problem?

Yes 1 No 2

b) If she cries do you:

pick her up immediately 1

let her cry for a while, then, if she doesn't stop, pick her up 2

never pick her up until you are ready to do so 3

D5. Can you usually calm your child when she cries?

no 1

yes, usually fairly easily 2

yes, but it takes a while 3

yes, after much effort 4

SECTION E:VOMITING AND STOOLS

E1. Does she ever posset (bring up small vomits)?

yes often 1 | yes some-times 2 | no 3 | don't know 9

E2. Has she ever vomited (brought up most or all of her meal)?

yes often 1 | yes sometimes 2 | don't know 9

yes once 3 | no not at all 4

E3. How many times a day (24 hours) does she usually dirty her nappy nowaday s?

4 or more imes a day 1 | 2 - 3 times a day 2 | once a day 3

once in 2-4 days 4 | once a week 5 | can't say 9

E4. How often are her stools:

		Always	Sometimes	Occasionally	Never
a)	hard	1	2	3	4
b)	soft	1	2	3	4
c)	curdy	1	2	3	4
d)	liquid	1	2	3	4
e)	brown	1	2	3	4
f)	green	1	2	3	4
g)	yellow	1	2	3	4
h)	other (please describe)	1	2	3	4

....

E5. a) Has she ever been ill with diarrhoea or gastro-enteritis?

Yes 1 No 2 **If no, go to Section F.**

If yes,

b) how many times?

c) how many days did the worst bout last?

d) Did you:

		Yes	No
i)	call the GP out	1	2
ii)	go to your GP	1	2
iii)	contact health visitor	1	2
iv)	ask chemist	1	2
v)	other (please describe)	1	2

....

e) Did you continue feeding as usual?

Yes 1 **If yes, go to E5.f**

No 2

If no, i) how long was normal feeding disturbed?

less than 1 day 1

1 day 2

2 days 3

3-4 days 4

5 or more days 5

E5. f) Was the baby treated with an oral rehydration solution?

Yes 1

No 2 **If no, go to F1.**

Don't know 9

If yes, i) give type if known: ....

ii) how long was the solution given?

less than 1 day 1

1 day 2

2 days 3

3-4 days 4

5 or more days 5

g) What other treatment was given?

....

....

SECTION F:YOUR BABY'S HEALTH

F1. Has your baby had any of the following since she was born:

		Yes	No
a)	jaundice	1	2
b)	sticky or crusty eye(s)	1	2
c)	high temperature	1	2
d)	jittery or twitching	1	2
e)	snuffles	1	2
f)	cough	1	2

F2. a) Have you asked the doctor to come to your home because of a problem with the baby?

Yes 1 No 2

If yes, i) how many times?

ii)what was wrong: ....

b) Have you consulted the doctor about any other problems with your child?

Yes 1 No 2

If yes, i) how many times?

ii)what was wrong: ....

F3. How would you describe the health of your baby now?

very healthy	1
healthy, but a few minor problems	2
sometimes quite ill	3
almost always unwell	4

F4. a) Has the baby had a rash in the joints and creases of her body (e.g. behind the knees, under the arms)?

Yes 1 No 2 Don't know 9

If yes,

b) how bad was this?

very bad 1 quite bad 2 mild 3 no problem 4

c) does she have this sort of rash now?

Yes 1 No 2 Don't know 9

F 5 a) Has she had an itchy, dry, oozing or crusted rash on the face, forearms or shins?

Yes 1 No 2

If <u>yes</u>,

b) how bad was this?

very bad 1 quite bad 2 mild 3 no problem 4

c) does she have this sort of rash now?

Yes 1 No 2

F 6 a) Has she had a nappy rash?

Yes 1 No 2 Don't know 9

If <u>yes</u>,

b) how bad was this?

very bad 1 quite bad 2 mild 3 no problem 4

c) does she have this sort of rash now?

Yes 1 No 2

F 7 a) Has she had cradle cap (scaly or crusty scalp)?

Yes 1 No 2 Don't know 9

If <u>yes</u>,

b) how bad was this?

very bad 1 quite bad 2 mild 3 no problem 4

c) was there redness with it?

Yes 1 No 2

d) was there itching with it?

Yes 1 No 2 Don't know 9

e) is there any cradle cap now?

Yes 1 No 2

F8. Please list all the ointments, pills and medicines that have been given to your baby **while she has been at home**:

1. ....
2. ....
3. ....
4. ....
5. ....
6. ....
7. ....
8. ....
9. ....
10. ....

Check: have you included ointments to prevent nappy rash, eyedrops, herbal remedies, etc.

F9. Have you taken your baby to the child health clinic?

Yes 1 No 2

F10. Has the health visitor visited you at home?

Yes 1 No 2

F11. Do you intend to immunise your baby?

yes, immunisation already begun 1 yes, but have not commenced yet 2 no 3

F12. Did the baby have vitamin K when she was born?

yes, injection 1 yes, by mouth 2 no 3 don't know 9

SECTION G: LOOKING AFTER YOUR BABY

G1. What sort of nappies do you use?

		Always	Sometimes	Never
a)	terry towelling	1	2	3
b)	disposable	1	2	3
c)	other type (please describe)	1	2	3

....

G2. Where did you get the things you use for your new baby?

		Bought new	Bought 2nd hand	Already had this	Given new	Given 2nd hand	On loan	Don't have
a)	pram	1	2	3	4	5	6	7
b)	carry cot	1	2	3	4	5	6	7
c)	cradle	1	2	3	4	5	6	7
d)	cot	1	2	3	4	5	6	7
e)	baby bath	1	2	3	4	5	6	7
f)	changing mat	1	2	3	4	5	6	7
g)	blankets	1	2	3	4	5	6	7
h)	car seat	1	2	3	4	5	6	7

G3. Have you applied for money from social services to help you buy any of this?

Yes 1 No 2

G4. Were you given money by social services to help you buy any of these?

Yes 1 No 2

SECTION H:ABOUT YOUR BABY

H1. Babies vary a lot in how soon they do things. Nowadays, how often does your baby:

		Often	Some-times	Rarely	Never
a)	look at your face when you feed her	1	2	3	4
b)	follow you with her eyes	1	2	3	4
c)	smile	1	2	3	4
d)	laugh	1	2	3	4
e)	squeal	1	2	3	4
f)	lift her head when on her tummy	1	2	3	4
g)	touch her hands together	1	2	3	4
h)	startle when she hears a sound	1	2	3	4

H2. Do you feel your baby knows you?

Yes 1 No 2 Not sure 3

H3. Do you feel your baby prefers you to other people?

Yes 1 No 2 Not sure 3

Below are some words used to describe babies. Please indicate how much your baby is like these descriptions.

H4.

		Very like my baby	Like my baby	Unlike my baby	Very unlike my baby	Can't say
a)	placid	1	2	3	4	5
b)	communicative	1	2	3	4	5
c)	grizzly	1	2	3	4	5
d)	fretful	1	2	3	4	5
e)	demanding	1	2	3	4	5
f)	angry	1	2	3	4	5
g)	cuddly	1	2	3	4	5
h)	active	1	2	3	4	5
i)	sociable	1	2	3	4	5
j)	withdrawn	1	2	3	4	5
k)	stubborn	1	2	3	4	5
l)	unresponsive	1	2	3	4	5
m)	happy	1	2	3	4	5
n)	alert	1	2	3	4	5

H5. Often mothers are surprised how long it takes to love their babies. How long has it taken you?

I loved her immediately 1 | it took a little while 2

it took over a week 3 | I still do not love her fully 4

can't remember 9

Space for any comments:

I1. This questionnaire was completed by:(tick all that were involved)

a) mother 1

b) father 1

c) other 1
(please describe).......................................

I2. Please give the date on which you completed this questionnaire:

day month year

199

I3. Please give the date of birth of your baby:

day month year

199

THANK YOU VERY MUCH FOR YOUR HELP

Please remember, because this is strictly confidential, the people who look at this booklet will not know your name. They will be unable to give you any help or contact anyone after reading what you have written. If you feel you need advice your General Practitioner or Health Visitor should be able to help you.

When completed, please return the questionnaire to:

Dr. Jean Golding,
Children of the Nineties - ALSPAC,
Institute of Child Health,
24, Tyndall Avenue,
Bristol.
BS8 1BR. Tel: (0272) 256260
